# High-resolution autosomal radiation hybrid maps of the pig genome and their contribution to the genome sequence assembly

**DOI:** 10.1186/1471-2164-13-585

**Published:** 2012-11-15

**Authors:** Bertrand Servin, Thomas Faraut, Nathalie Iannuccelli, Diana Zelenika, Denis Milan

**Affiliations:** 1 Laboratoire de Génétique Cellulaire, Animal Genetics Division, INRA, Chemin de Borde-Rouge Auzeville, 31326 Castanet-Tolosan, France; 2, Centre National de Génotypage, 91057 Evry, France

## Abstract

**Background:**

The release of the porcine genome sequence offers great perspectives for Pig genetics and genomics, and more generally will contribute to the understanding of mammalian genome biology and evolution. The process of producing a complete genome sequence of high quality, while facilitated by high-throughput sequencing technologies, remains a difficult task. The porcine genome was sequenced using a combination of a hierarchical shotgun strategy and data generated with whole genome shotgun. In addition to the BAC contig map used for the clone-by-clone approach, genomic mapping resources for the pig include two radiation hybrid (RH) panels at two different resolutions. These two panels have been used extensively for the physical mapping of pig genes and markers prior to the availability of the pig genome sequence.

**Results:**

In order to contribute to the assembly of the pig genome, we genotyped the two radiation hybrid (RH) panels with a SNP array (the Illumina porcineSNP60 array) and produced high density physical RH maps for each pig autosome. We first present the methods developed to obtain high density RH maps with 38,379 SNPs from the SNP array genotyping. We then show how they were useful to identify problems in a draft of the pig genome assembly, and how the RH maps enabled the problems to be corrected in the porcine genome sequence. Finally, we used the RH maps to predict the position of 2,703 SNPs and 1,328 scaffolds currently unplaced on the porcine genome assembly.

**Conclusions:**

A complete process, from genotyping of a high density SNP array on RH panels, to the construction of genome-wide high density RH maps, and finally their exploitation for validating and improving a genome assembly is presented here. The study includes the cross-validation of RH based findings with independent information from genetic data and comparative mapping with the Human genome. Several additional resources are also provided, in particular the predicted genomic location of currently unplaced SNPs and associated scaffolds summing up to a total of 72 megabases, that can be useful for the exploitation of the pig genome assembly.

## Background

With the important reduction in cost of sequencing, whole genome sequencing data are becoming much easier to produce. However sequencing projects still need medium to long range information to anchor scaffolds on chromosomes and produce high quality reference maps [1]. The Pig (*Sus scrofa domestica*) genome sequencing project used high resolution physical maps based on a combination of restriction fingerprint maps and BAC end sequencing together with state-of-the-art Human-Pig comparative maps [2,3]. Although these maps enabled coverage of over 98% of the 18 pig autosomes with 139 contigs [3], their internal ordering and orientation needed validation from another independent source. RH mapping is a widespread physical mapping approach that has been frequently used to assist genome assembly [4-11]. In the pig, two radiation hybrid panels are available, at different radiation doses: 7,000 rads [12] and 12,000 rads [13], with estimated resolutions of 35.4 Kb/cR and 12.5 Kb/cR respectively each of which was produced from multiple animals [13]. These panels were used to construct whole genome [14-16] as well as localized (*e.g.*[17-23]) radiation hybrid maps.

Recently, a high density SNP array was produced for the pig [24], allowing the simultaneous genotyping of 64,232 SNPs in one individual. Compared to previous RH mapping strategies focusing on ESTs or microsatellites, the use of high density SNP arrays has several advantages, in particular in the context of genome assembly. First the number of genotyped loci is large, close to if not above the number that the resolution power of the RH panels allows to order. Second, these SNPs correspond to sequences that are, by design, known to be unique on the pig genome. Finally, the distribution on the genome of the SNPs is roughly homogeneous, covering equally gene rich and gene poor non-repetitive regions.

We describe here genome-wide high resolution RH maps of the pig autosomes constructed by genotyping the two pig RH panels with the Illumina porcineSNP60 array. The construction of RH maps in this context presented two challenges: the accurate genotype calling from the raw intensity data and the construction of chromosome maps with thousands of markers. Because answering these questions is error prone, we validated the map ordering using genotyping data in families. Once confident in the validity of our RH maps, they were compared to a draft version of the pig genome assembly (build9) which led to propose improvements that were included in the build10 assembly. Finally we show the added value of RH maps for the mapping of unassigned sequences: we propose likely positions for 1328 unplaced scaffolds totaling 72 megabases of genome sequence.

## Results

### Radiation Hybrid Maps of the Illumina PorcineSNP60 array

The two pig radiation hybrid panels [12,13], making up a total of 180 hybrid clones (90 clones in each panel), were genotyped using the Illumina porcineSNP60 array. RH vectors were constructed for more than 50,000 SNPs out of which 42,299 could be assigned a position on the autosomes of the build9 assembly of the pig genome (M. Groenen, personal communication). Details on the genotyping procedure are provided in the Methods section.

The main objective of this study was to compare the SNP order as defined by the RH maps to the one defined by a preliminary version of the pig genome assembly in an attempt to identify discrepancies that could pinpoints some assembly problems. We provide details on the methods used to build RH maps in the Methods section.

Briefly, for each autosome, the analysis was done in three separate steps:

1.We confirmed the linkage between markers, using RH data alone, and remove unlinked markers. This step led to the removal of 570 SNPs.

2.We built comparative RH maps [25] with all remaining markers, using prior information from the build9 assembly. This resulted in 41,729 SNPs being positioned on 18 chromosomal maps.

3.We extracted a subset of markers from the initial RH maps for which the order was strongly supported by RH data. This procedure removed about 3,000 SNPs that could not be confidently ordered and led to RH *robust maps*[26] comprising 38,379 SNPs (Additional file 1).

**Figure 1:**
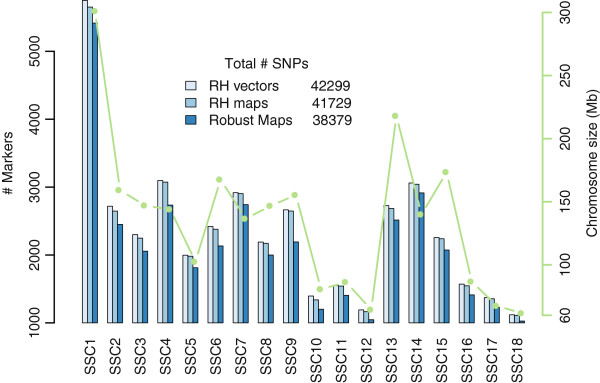
**Number of markers on RH maps and chromosome size.** Blue bars: Number of Single Nucleotide Polymorphisms mapped on the pig chromosomes at different steps of the mapping process (left axis). Green line: estimated chromosome size [3] (right axis).

The construction of robust maps follows the same rationale as the construction of framework maps (see Methods). Figure 1 recapitulates the number of SNPs kept at each step for each chromosome. Although the number of SNPs varies across chromosomes, the number of SNPs removed in the process of constructing robust maps was relatively constant across chromosomes, with the notable exception of chromosome 9 for which a large portion in the middle of the chromosome could not be kept in the robust map. The 38,379 SNPs are assigned to 36,165 positions (94%) that are distinct in one of the panels. For SNPs that cannot be separated by either panel the order given by our map reflects only the assembly information. Figure 1 jointly shows the size of pig chromosomes in centi-Rays as inferred by the robust maps and the estimated size in megabase [3]. Note that on the pig karyotype, chromosomes are ordered by morphology first and then approximate size: chromosome 1 to 7 are submetacentric, 8 to 12 metacentric and 13 to 18 acrocentric. Generally, the number of SNPs on a chromosome varies with the size with the notable exceptions of chromosomes 6, 13 and 15 that seem to carry fewer SNPs than expected given their size. Detailed characteristics of the final RH maps are provided in Additional file 2.

### Validation of RH maps with genetic data and analysis of assembly build9

**Figure 2:**
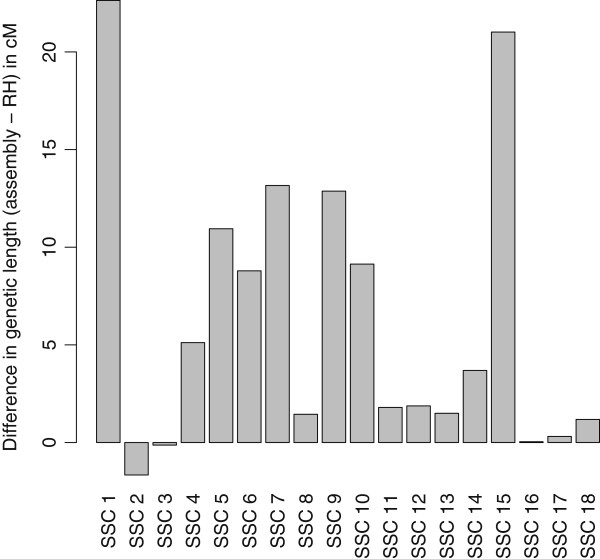
**Difference in genetic length between assembly build9 and RH maps.** Difference between the genetic lengths (in centiMorgans) of pig autosomes when using the build9 assembly and the RH maps. A positive (resp. negative) value indicates an increased genetic length with the assembly (resp. RH) order.

z To validate the orders of the RH maps, we used genetic data from 263 small half-sibs families, forming a total of 728 meioses (Table 1). Each family consisted of a male parent and at least 2 male offspring, each having a different mother, except for the Meishan breed for which families were nuclear families. We estimated the genetic lengths of chromosomes using either the robust RH maps order or the assembly order, with the same SNPs. We used a procedure for detecting crossing-overs [27] adapted to half-sib families [28]. Given the structure of our data, estimates of recombination rates pertain only to male recombination and are averaged across multiple breeds. We found that the genetic lengths of all chromosomes but chromosome 2 and 3 were greater for the build9 assembly (Figure 2), *i.e.* RH maps were generally more parsimonious than the assembly map in the number of recombination events needed to explain the genetic data. This suggested that the build9 assembly contained errors that could possibly be corrected using our RH maps.

**Table 1:** Breed of origin of families used for recombination rate estimates

**Breed**	**Number of sires**	**Number of meioses**
Large White	71	198
Pietrain	53	113
Sino-European line	41	122
Landrace	32	91
Duroc	29	84
Meishan^ † ^	13	18
Other	24	102
Total	263	728

^†^Meishan families are nuclear families.

**Figure 3:**
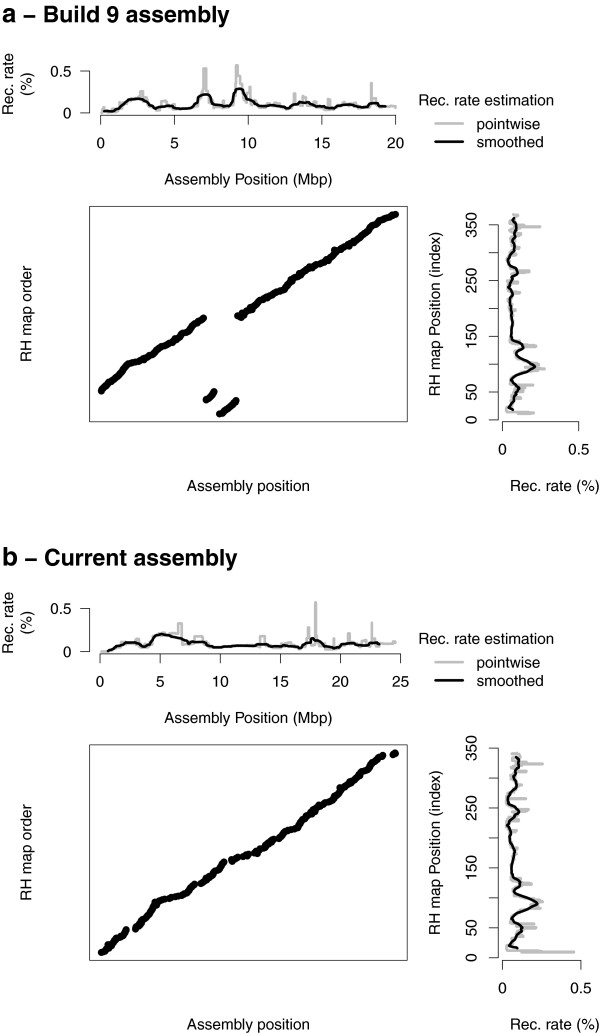
**Comparison of two pig genome assemblies and the RH map at the beginning of SSC15.a**) Assembly build9 and **b**) current assembly. For each assembly comparison, the top (resp. right) plot presents the recombination rate along the assembly (resp. RH map). The middle dotplot compares the positions of SNPs on the two maps.

Our analysis of recombination on genetic data allows us to estimate the probability that a crossing over occurs in a particular interval on a map. We used this information to examine in more details regions involved in the differences in genetic length between the assembly and the RH maps: we focused on intervals exhibiting both a discrepancy (a breakpoint) between the assembly and the RH map and a high recombination rate. We distinguished several types of differences. First, we observed cases where large differences in placement were found for a single SNP or a small number of SNPs. This was frequently seen on chromosome 1, the largest chromosome by far, and accounts for 9 cM of the 22 cM difference between genetic lengths of the assembly and the RH order for this chromosome. Because this problem affects typically a single SNP at a time, it can be due to an error in the mapping of the SNP sequence to the chromosome rather than an error in the assembly. Second, we observed high recombination rates for some regions located at the end of the chromosome while they were typically placed within the chromosome on the RH map. We know that in the assembly process sequences assigned to a chromosome but with no strong information on the localization were placed at the end by default. Finally, we observed some differences for large chromosome segments probably indicating problems in the BAC-based physical map. This is the case for example on chromosome 15 where the large discrepancies come together with large increases in recombination rates in the corresponding region of build9 (Figure 3.a).

**Figure 4:**
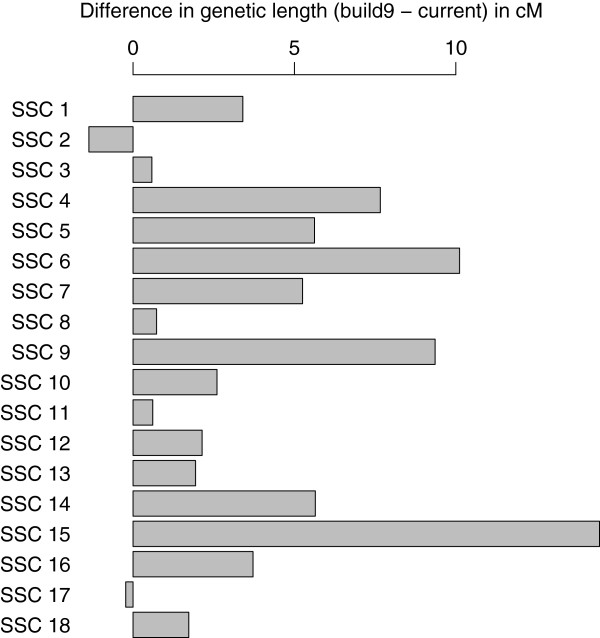
**Difference in genetic length between current assembly and** **build9 assembly.** Difference between the genetic lengths (in centiMorgans) of pig autosomes when using the current pig genome assembly and the build9 assembly. A positive (resp. negative) value indicates an increased genetic length with the build9 (resp. current) assembly order.

Based on these comparisons we listed a set of proposed improvements to the pig genome assembly. Most of these improvements were the basis of changes in the pig genome assembly, when they were compatible with sequencing data (*e.g.* they did not involve breaking contigs or scaffolds). As an illustration of such an improvement, Figure 3 represents a region of chromosome 15 both in the build9 (a) and build10 (b) assembly order. For all autosomes, we provide detailed pictures illustrating comparisons of RH maps with assembly build9 and with assembly build10 (Additional file 3). Most chromosomes have a much smaller genetic length with the build10 assembly order than with the build9 assembly order (Figure 4). Only two chromosomes, 2 and 17, exhibit an increased genetic length of about 1 cM with the build10 assembly order, but because of the limited resolution of our genetic data, we do not think those differences necessarily imply new errors in the current assembly compared to build9.

### Mapping of unplaced SNPs

**Figure 5:**
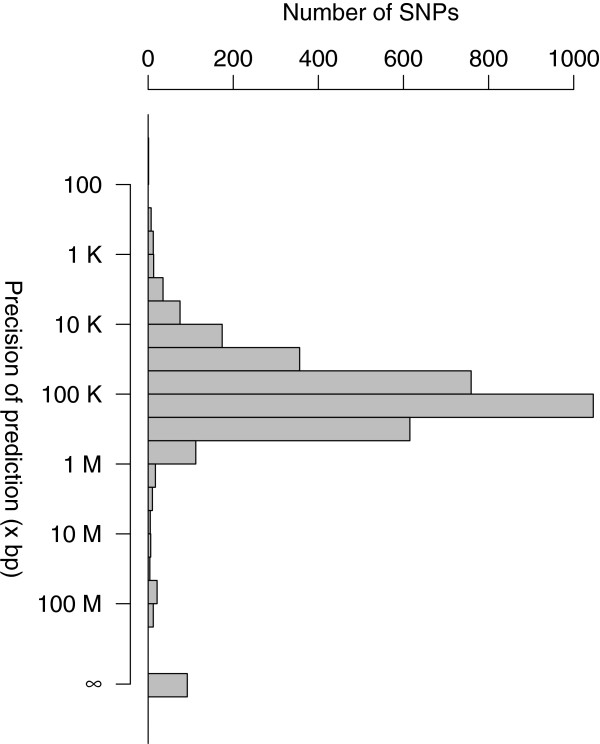
**Precision of predicted uSNPs positions on the pig genome.** Empirical differences between the predicted position of SNPs based on RH data and its true position on the pig genome assembly. The ∞ symbol marks SNPs assigned to a wrong chromosome. Details of the prediction procedure are given in the text.

As an application of the RH maps, we studied the possibility of predicting the positions of SNPs with no localization on the build9 assembly, either because they were mapped to unplaced scaffolds or because they could not be reliably aligned to the assembly scaffolds. We will call these *unmapped* SNPs “uSNPs” herein. Our approach was to find the best possible position on our RH maps for each of the uSNPs, by first identifying the most likely chromosome and then the most likely position. We used a resampling approach to estimate confidence thresholds for this assignation. A detailed description of the procedure is given in the Methods section below. On the build9 assembly, 6788 uSNPs had an RH vector. We were able to predict a position for 6076 of these SNPs, hence a prediction rate of about 90%. Given that we did not try to assign uSNPs to the X chromosome, whose length encompasses about 6% (140 Mb) of the pig genome sequence, this rate met our expectations. We initially conducted this analysis on the build9 genome assembly. The released pig genome has improved a lot and 3706 of the uSNPs with RH vectors are now located on the pig genome. This provided us with the possibility to evaluate the performance of our prediction. We found that, among these 3706 uSNPs, 2.6% were assigned to the wrong chromosome by our procedure and 2.4% were assigned a position more than 1Mb from their true location. Thus, our procedure comes with an error rate of about 5%, which seems reasonable. Note that this does not imply that positioning of SNPs on robust maps come with a similar error rate. First, for these SNPs we have a concordance between the position given by the genome sequence (information missing for uSNPs) and the RH data. Second, the procedure to establish robust maps is much less prone to errors than the simple approach used for uSNPs. Looking in more details at the precision of our localization (Figure 5), we found that most SNPs lie within a few hundreds kilobases from their true position, a figure that is compatible with the resolution of the RH panel.

**Figure 6:**
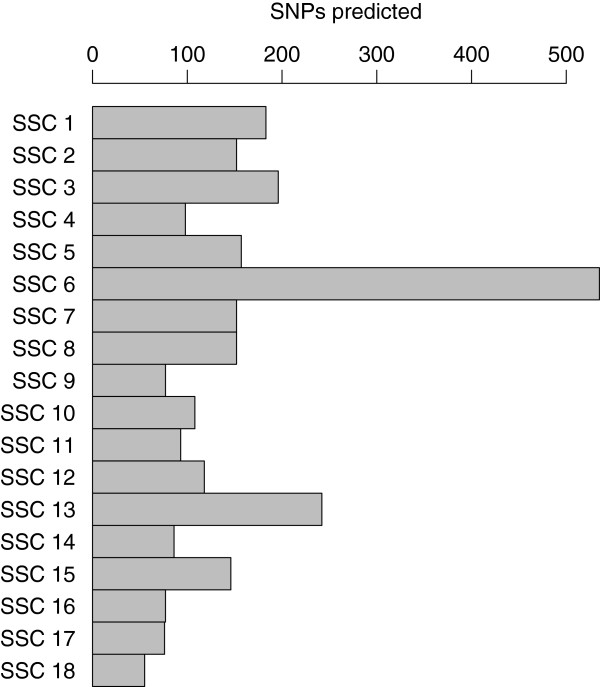
Number of uSNPs predicted for each pig autosome.

As our procedure seemed to provide good results, we studied further the positions of the 3082 uSNPs remaining unmapped on build10 of the pig genome sequence. Out of the 3082, we could predict a position for 2703 uSNPs (Additional file 4). Interestingly, the chromosomes for which we predict the most SNPs (Figure 6) are chromosomes 6 and 13, two of the chromosomes that we found carrying less SNPs than expected (Figure 1). This could imply that for these chromosomes, more genome sequence is missing in the current assembly than for others.

We applied the same prediction procedure to the 570 SNPs that were initially discarded in the construction of RH maps as they did not show evidence of linkage with the other SNPs. Because the confirmation of linkage was conducted within chromosomes (see Methods), this lack of linkage can be due either to wrong assignments of SNP sequences to chromosomes or to genotype calling errors. Applying our prediction procedure provided mixed results. First, only 44% (252) of the 570 SNPs could be assigned a chromosome, much lower than the 90% success rate obtained on the uSNPs. Among the 252 SNPs, 44% (117) were predicted a different chromosome than on build9. This figure goes down to 19% with build10, which indicates some success in our prediction. However, if we consider this proportion as an error rate, like we did for uSNPs, then it is much higher than for uSNPs (2.6%). This suggests that genotype calling errors contribute significantly to the linkage problem initially identified and that the prediction for these SNPs is not reliable enough for assignment.

### Mapping of unplaced scaffolds to the pig genome

Among the 2,703 SNPs for which we could assign a position on the pig genome, we were able to align 1,947 to 1,428 scaffolds present in the pig genome sequence but currently unassigned. In total, this represents 79 Megabases of pig genome sequence. As a first validation, we noticed that essentially all SNPs assigned to a given scaffold had consistent predicted localization on the pig genome (same chromosome and same position). Only one SNP on a single scaffold (chrU_scaffold3109) had a different chromosome than the 3 other SNPs of this scaffold. The SNP was therefore removed from the analysis. We note however that there is a chance that this scaffold is chimeric as its sequence aligns to two distant regions of Human chromosome 1 (at 6 and 206 Mb). Being aware of the potential errors in localization mentioned above, we used comparative genomics to validate our predictions. We aligned the candidate scaffolds to the Human genome and identified the surrounding homologous region on the pig genome from conserved syntenies predicted by the Narcisse comparative genome browser [29]. We classified the validation results into 5 categories (Table 2):

scaffolds for which the position predicted by comparative genomics did not match the same chromosome as our prediction (*discordant*). These adds up to 7% of the total number of scaffolds and 8.7% of the total sequence length. Given that there might also be errors in the comparative genomics prediction, these values can be considered quite close to the estimated 5% error rate above.

scaffolds that could not be aligned on the human genome (*noinfo*) or for which the corresponding human genome sequence has no identified homologous region on the pig genome (*nosynt*). There is insufficient evidence to validate or invalidate our predicted position for these scaffolds. These are typically small scaffolds as, while they represent 27% of the number of unassigned scaffolds considered, they only cover 7.8% of the sum of their sequence lengths (Table 2).

scaffolds with the same predicted chromosome by RH maps and comparative genomics. Within these scaffolds, we distinguish between those that have predicted positions separated by less than 1 megabase (*valid*) and more (*sscvalid*).

**Table 2:** Validation of the unplaced scaffolds positioning

**Category**	**Number**	**Proportion (%)**	**Sequence length**		**Number of uSNPs**
			**Megabases**	**Proportion (%)**	
**Discordant**	**100**	**7.0**	**6.9**	**8.7**	**149**
**Tentatives**	**1328**	**93.0**	**72.3**	**91.3**	**1798**
noinfo	197	13.8	1.2	1.5	213
nosynt	187	13.1	5.0	6.3	249
sscvalid	166	11.6	12.4	15.6	242
valid	778	54.5	53.7	67.8	1094

Categorization of unplaced scaffolds positioned on the pig genome. The table presents the number of scaffolds in the different validation categories (based on comparative genomics with the human genome): discordant , not mapped on the human genome (noinfo), mapped on the human genome but in a region not found on the pig genome (nosynt), on the same chromosome but at a substantially different (***≥*** 1*Mb*) location (sscvalid), and concordant chromosome and position (valid). The Tentatives category correspond to the sum of noinfo , nosynt , sscvalid and valid.

Overall, if we consider scaffolds that are not discordant (*tentative* scaffolds), we can map more than 72 megabases of unassigned scaffolds on the pig genome. If we consider only scaffolds with the most robust prediction (*valid* category), they still add up to more than 53 megabases. Predicted positions and category of the *tentative* scaffolds are given as in Additional file 5.

To provide an example of how to use this information, we illustrate the case of scaffold GL894031, the unplaced scaffold with a *valid* category that carries the largest number of SNPs (10). The predicted localization for this scaffold, based on SNPs predicted positions, is on chromosome 6, between 91.1 and 91.9 Mb. Within this region, the pig genome has a large gap, identified as a 50Kb stretch of ’N’ nucleotides. We verified that scaffold GL894031 aligned on the putative syntenic region of the Human genome and constructed a local RH map of the region including the scaffold SNPs (Additional file 6). This analysis validates the predicted position for this 253Kb scaffold and shows it can be placed on the pig genome confidently.

## Discussion

In this study, we present what we believe is the first example of constructing genome-wide high resolution RH maps from SNP array data. Our main motivation for building these maps was to provide independent data to analyze and validate the pig genome sequence. Before proposing improvements to the pig genome, we carefully validated our results using information on segregation data in pig families. Eventually, this made our inference more robust and allowed us to contribute significant improvements to the pig genome: we proposed modifications for the largest discrepancies and the assembly was modified when this did not contradict sequence data (*e.g.* breaking a contig). We discuss here what we believe are important aspects of the current study: genotyping an RH panel with a high density SNP array, construction of high (ultimate) resolution genome-wide RH maps and finally the analysis of discrepancies between maps and assemblies.

### Genotype calling from SNP array data

Key to the success of RH mapping in general and for this study in particular is the ability, for each marker, to confidently distinguish between its presence or absence in each clone of the panel thereby providing the retention profile used for constructing maps. In this context, and in order to reduce the risk of false negative/positive calling and its severe impact on the subsequent linkage analysis, PCR-based genotyping is usually performed in duplicates, scoring discrepancies as unknown. In contrast to the binary outcome of PCR, the raw intensities provided by the Illumina genotyping platform enabled a calling procedure to be devised based on a continuous measure. The full distribution of signals across SNPs and clones was used in an attempt to control the false positive/negative rate by scoring intermediate intensity values as unknowns (see Methods). This can also be seen as using all other data points when calling a particular SNP genotype in a single clone. This genotype calling procedure from intensity data obtained by SNP array genotyping can certainly be improved in future studies. In particular, it would be interesting to try to separate the different effects of clones, SNPs and arrays on the observed intensities in order to provide better prediction of the genotypes, possibly reducing missing data and genotyping error rates. This would require the development of new statistical methods and the genotyping of at least some clones on multiple arrays.

### On the resolution of RH maps

In RH mapping, the precision of a map or a mapping tool is generally characterized by the *resolution* expressed as a Kilobases to centiRay ratio. Based on our RH maps, we estimate the resolution of the IMpRH panel at 8.6 Kb/cR and of the IMNpRH2 panel at 5.3 Kb/cR, whereas previous estimates were respectively 35.4 Kb/cR and 12.5 Kb/cR [13]. This difference can be explained by two reasons. First, the number of markers in this study is much larger than in any previous analysis. It is indeed well known that an increase in marker density causes map inflation and hence observing a decrease in the Kb/cR ratio when increasing marker density is a classical behavior of chromosomal maps. Second, we use a comparative mapping approach that incorporates, in the optimization criteria to construct RH maps, a prior information of a reference order given here by the genome assembly. As such, maps obtained are not the most parsimonious in breakpoints, *i.e.* not the map of smallest length (in cR). Again, this has the consequence of decreasing the Kb/cR ratio.

The resolution can however be understood in a broader sense than this simple Kb to cR ratio (see Additional file 7). When constructing high-resolution maps, a natural question arises: what is the maximum number of markers that we can expect to order? This depends of course on the design of the mapping experiment and we address the question in the context of our study where two radiation hybrid panels were used. Using estimates of the resolution parameters above, we can compute the theoretical proportion of markers that can be assigned distinct positions in RH maps (Additional file 7), using three assumptions: (i) the RH order is the true order (ii) markers are evenly spaced on the genome and (iii) RH vectors have no missing data or genotyping errors. In our case, this theoretical proportion is 99.7% and we observe a value of 94.2% (Additional file 7). We consider this difference as reasonable given that none of the three hypotheses strictly holds in real data.

Designing an RH mapping experiment requires to define what is the desired resolution, *i.e.* what is the typical physical distance (in Kilobases) between markers that are to be ordered. For example in the case of ordering the scaffolds of a genome assembly, the N50 or N90 scaffold size could be the relevant target resolution. The resolution of an RH panel depends on the panel size and the resolution parameter expressed in Kilobases per centiRay. This parameter is related to the radiation dose (expressed in Rads) but through a process too complex to be modeled so its value can only be guessed from previous studies. However, estimates obtained from the literature must be taken with caution. First they were most likely obtained in other species. Furthermore, as we have shown, these estimates depend on the number of markers and the mapping methods used. Overall, adjusting the resolution through the radiation dose is going to be imprecise. In previous RH panels construction experiments, the panel size was purposely limited to 90 clones because of PCR genotyping where a single marker is genotyped for all clones disposed on a 96-well plate (with wells reserved for control samples). SNP array genotyping however proceeds by genotyping all SNPs on a single clone and therefore does not impose such a design so panel sizes can be made larger to increase the resolution. Given a panel size and resolution parameter, Additional file 7 provides the equations allowing to derive the expected number of markers that can be mapped to distinct positions. For example, we estimate that using the two pig panels, up to 250K markers could be mapped on the autosomes (see Additional file 7 for details). However, it would require obtaining RH vectors for about 1 million SNPs because there is a trade-off between increasing the number of markers interrogated and decreasing the probability of separating adjacent markers. Above 100K SNPs, there is a strong diminishing return in the proportion of markers that can be mapped among the genotyped markers. The numbers of separable markers above depend on the characteristics of the panels used here. It can be increased, in particular by using panel with more than 200 clones. However this may be prohibitively expensive and our general conclusion is that using arrays larger than 100K SNPs is not going to be cost-effective for producing high-density RH maps in most situations.

### Discrepancies between maps and assembly

The resolution of discrepancies directly addresses the question of the reliability of the order defined on one side by the genome map and on the other by the assembly. The construction of robust maps was precisely designed to address the reliability of RH maps [26]. On the assembly side, the process is clearly too complicated, involving different technologies such as sequence assembly or physical mapping resources, to enable the development of confidence measure for the organization of sequences in a particular region. A reasonable step is certainly to differentiate the different components of the assembly such as the contigs and scaffolds on one side and their organization along chromosomes on the other side. The modifications of the preliminary assembly (build9) proposed in this study only involved reordering of scaffolds along chromosomes. Note however that our approach could potentially contribute to the identification of chimeric scaffolds. Another approach to resolve contradicting orders is the exploitation of additional and independent source of information such as the genetic data used in this study. Finally, some of the remaining inconsistencies could be biologically grounded, reflecting individual structural variations. The reference sequence and the RH panels were indeed constructed using the DNA from different individuals and from different breeds (a Duroc for the reference sequence and Large White for the panels). Preliminary studies in pigs have demonstrated the existence of a considerable level of between-breed variation [30].

### The particular case of the X chromosome

The X chromosome was not investigated in this study because it requires a specific analysis. First, both RH panels were constructed using male DNA hence with a single X chromosome and therefore a reduced retention in comparison to other chromosomes, with the exception of the pseudo-autosomal region which is believed to cover a small fraction of the X chromosome (∼5% [31]). Second, the X chromosome harbors the HPRT gene used as the selection locus leading to a retention fraction in its neighborhood which requires specific attention for the construction of maps [32]. Finally, our validation procedure, using genetic data and based essentially on the observation of male meioses is not applicable here. For these reasons, we reserve the construction of an RH map for this chromosome and associated analysis for future work.

## Conclusions

Overall there is good agreement between the current genome assembly and the robust RH maps presented here. Although the pig genome sequence is now released, we believe our RH maps can still be useful to the community. There are likely to still be ordering problems on the pig genome, as has been seen for other mammalian genomes before, and the RH information can provide the pig genetics community with valuable information to help improve further the pig genome sequence. Also, the RH maps allow the positioning of currently unassigned SNPs and sequence to the pig genome, which effectively increases the coverage of the assembly, allowing for example the mapping of significant genome-wide association signals currently positioned on an unassigned scaffold. Our RH maps have already proved to be useful to study the recombination rate patterns in the pig [28], by providing a robust ordering of markers which is crucial for recombination rate estimation.

The approach used for this study relies on the availability of both RH panels and a high density SNP array. For species where both tools are available, this study demonstrates that high density RH panels are very useful for providing physical evidence for the ordering genome sequence and for positioning unplaced scaffolds. However, we do not expect that it can be applied as presented here to a large number of species. Indeed, the production of an RH panel, let alone two, is a labor intensive, highly technical and therefore expensive task. High density SNP array also come with a large designing cost and will likely be available for a small number of species. While we can expect an increasing number of genomes to be sequenced, even *de novo* thanks to NGS techniques, producing high-resolution genome maps based on non sequence data (such as RH maps, genetic maps, in situ hybridization etc.) will be essential for the production of good quality genome assemblies. This will most likely require bypassing the limitations mentioned above, for example by substituting SNP arrays by Genotyping By Sequencing techniques and radiation hybrids by other chromosome breaking mechanisms such as genetic recombination in large samples. The methods used here can readily be applied to such datasets.

## Methods

### Genotype calling

We genotyped two RH panels with the Illumina porcineSNP60 chip. In the same experiment, one sample of pig genomic DNA and hamster genomic DNA were added as controls. Radiation hybrid samples contain only a fraction of the total genomic DNA found in a cell, as such, the genotype calling methods used on genomic DNA samples are not adapted for radiation hybrid data. We used a specific methodology for calling the RH genotypes: the principle is to identify markers for which raw signal intensities show a clear-cut difference between the negative hamster control and the positive control.

**Figure 7:**
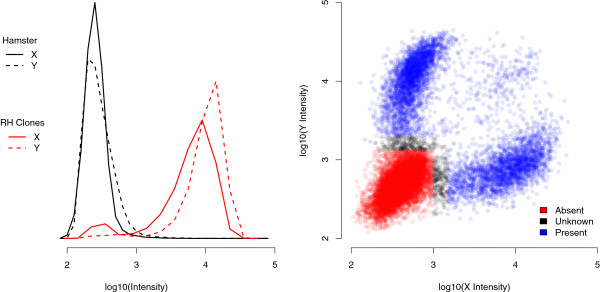
**Genotype calling from intensity data.** Left: Distribution of signal intensities among SNPs in the hamster control (black lines) and averaged over RH clones (red lines). Right: Distribution of signal intensities colored according to genotype calls. For clarity only a random subset of 20,000 data points is plotted.

We started with a data set of 59,950 SNPs for which at least 5 genotypes were initially called in the 180 radiation hybrids. We then compared raw intensities on the X and Y axes (for two SNP alleles) in the hamster and averaged over RH clones (Figure 7, left). As we could observe mean intensities as low as 500 on the X axis and 1000 on the Y axis within RH clones, any SNP exceeding these thresholds in the hamster was discarded. We also discarded SNPs for which the mean intensity in RH clones was less than 3000 on the Y axis or less than 1500 on the X axis, because we lacked the discrimination power to distinguish clones that retained the SNP and clones that did not.

For the remaining SNPs, we determined thresholds for genotype calling based on the observed distribution of raw intensities (Figure 7, right): signal with an X intensity less than 1000 and Y intensity less than 1300 were called absent, signals with an X intensity greater than 1500 or a Y intensity greater than 2000 were called present; other signals correspond to intermediate values and the corresponding genotypes were set as missing. Finally, we eliminated SNPs for which a genomic control was negative (186) or dubious (92) as well as SNPs presenting more than 10 unknown genotypes over 180 clones (3219). Eventually we produced RH vectors for 50220 SNPs. The proportion of SNPs passing these filters was constant across autosomes (between 85% and 90%). However, for SNPs that were not assigned a chromosomal position on build9, about 40% of the SNPs failed these filters.

### Construction of Radiation Hybrid maps

We first partitioned SNPs according to their assigned chromosome on assembly build9. For each autosome, we determined linkage groups among SNPs, based on RH data alone. Specifically, we used the multigroup option of the carthagene software [33], requiring a minimum LOD score of 6 in each panel, and keeping only linkage groups of size greater than 10. This resulted in typically few (less than 5) linkage groups per chromosome. We treated them jointly using a method [25] that combines *a priori* information (here coming from the build9 assembly) and RH data as implemented in carthagene by merging the two RH datasets and the assembly dataset. Using this method guarantees that discrepancies observed between the RH map and the assembly are strongly supported by RH data, as regions were RH data are not informative keep the prior (*i.e.* the assembly) ordering. RH maps were built with the lkh command. On these initial RH maps 570 SNPs did not show clear evidence for linkage and were therefore removed to obtain RH maps totaling 41,729 SNPs.

We next applied a novel approach [26] to build maps with a highly reliable ordering, which we call *robust* maps. Briefly, the principle of the method is to obtain a set of possible maps with associated probabilities of being correct and then extract from this distribution a subset of markers that show the same ordering across maps of high probability. Specifically, we first estimated the posterior distribution of possible orders using an MCMC approach [26] implemented in the mcmc command of carthagene. We performed 5000 MCMC iterations and discarded the first 1000 as burning iterations. Finally we extracted robust maps from the posterior distribution using the metamap software [26], with default values for all parameters. This approach is closely related to the idea of constructing framework maps. In the case of framework maps, an order is accepted based on a maximum likelihood ratio, also called LOD, criteria: the logarithm of odds between the best order and the second best order must exceed a preset ratio, such as 1,000:1 for example (LOD-3) [34]. In contrast, the construction of robust maps falls into the Bayesian paradigm [35].

### Predicting the position of uSNPs on the pig genome

6076 SNPs with RH vectors were not assigned to a chromosome on build9. Here we describe how we used the RH data to assign them to the pig genome and to predict their position on the pig genome assembly. The robust RH maps that we developed are composed of a subset of markers for which a position on build9 was known (M. Groenen, pers. comm.). Given the high number of markers, producing maps *de novo* would require a very long time. We decided to use a more rapid approach, which would be good enough to provide reasonable predictions.

The principle of the mapping of an unassigned SNP (uSNP) on a pig chromosome is based on computing a similarity score between this SNP and all the already mapped SNPs (mSNPs). Given two SNP RH vectors (one uSNP and one mSNP), we count the number of clones which show the presence of both SNPs. We can derive a chi-square statistic for seeing N match (present,present) between the two markers, under the null hypothesis that the two markers are independent. The score used is -log10 of the pvalue corresponding to this statistic. We performed an empirical study to obtain a threshold for the score of unlinked SNPs. We sampled a mSNP on a chromosome and calculated its maximum score on another chromosome. Repeating this process a 1000 times provides a distribution of the scores of a SNP when tested against a wrong chromosome.

The first step of our analysis was to assign uSNPs to pig chromosomes. To this end, we calculated the similarity score of each uSNP with all mSNPs, using RH vectors of the lower resolution panel [12]. A uSNP was assigned to a chromosome when its score exceeded an empirical threshold. Given the empirical distribution above, a score of 5 seemed appropriate. However, this led to many uSNPs that could be assigned to more than one chromosome. Indeed, we had to use a score of 7 to get a single chromosome assignment for all SNPs (Additional file 8). At the end of this step we had a list of SNPs to map for each of the 18 autosomes. For each autosome, we calculated for each uSNPs assigned to this chromosome a score with all the mSNPs, using RH vectors of the higher resolution panel [13]. We identified the highest scoring neighbor as a first flanking marker and the second highest scoring SNP as its other neighbor. We then predicted its RH position as the barycenter, with weights equal to the scores, as a mean to give more weight to the highest scoring neighbor. At the end of this step, we obtained a list of uSNPs with a predicted chromosome and position on the RH map. The last step was to predict the position of the uSNP on the genome assembly. For this we first fitted a spline of the assembly position on the map position, using the mSNPs. We then used this spline to predict new positions for uSNPs, given their predicted position on the RH map.

### Software availability

All software and computer programs used in this study are freely available from the corresponding author.

## Competing interests

The authors declare that they have no competing interests.

## Authors’ contributions

BS carried out the analysis of the data and wrote the manuscript. TF contributed to the data analysis and writing the manuscript. DZ and NI carried out the sample preparation and genotyping. DM conceived the study, coordinated the genotyping and contributed to the data analysis and drafting the manuscript. All authors read and approved the final manuscript.

## Supplementary Material

Additional file 1 Radiation hybrid maps of the Illumina pig 60K SNP chip. This text file contains the position (in centiRays) of 38379 SNPs from the Illumina porcineSNP60 SNP chip on radiation hybrid maps. For each SNP, two positions are given, one on the IMpRH panel (pos.rh1) (Yerle et al., 1998) and one on the IMNpRH2 panel (pos.rh2) (Yerle et al., 2002). Click here for file

Additional file 2
Characteristics of the RH maps. This table contains detailed characteristics of the RH maps obtained for each chromosome: number of SNPs mapped, number of distinct position for each panel, resolution, average marker distance and retention fraction. Click here for file

Additional file 3 Detailed comparison of RH maps with the **build9** assembly and with the **build10** assembly. This file contains comprehensive pictures comparing (i) the build9 draft assembly and the RH maps and (ii) the pig genome sequence build10 and the RH maps. Click here for file

Additional file 4 Predicted positions on the pig genome for 2703 SNPs. This file contains a predicted chromosome and location in base pairs for 2703 SNPs currently not assigned a position on the pig genome. We estimate that 95% of these predicted positions lie at less than 1Mb of the true position, with a median at 120Kb. See details in the text. Click here for file

Additional file 5 Predicted positions on the pig genome for 1328 scaffolds. This file contains a predicted chromosome and location in base pairs for 1328 scaffolds currently unplaced on the pig genome. This files contains only the *Tentatives* scaffolds (see details in the main text). Click here for file

Additional file 6 Validation of the predicted position for scaffold GL894031. This file contains figures and text presenting the validation of the predicted position for the unplaced scaffold GL894031. Click here for file

Additional file 7 Resolution of RH mapping for high density arrays. This file contains theoretical calculations on the resolution of RH panels and the design of RH experiments. Click here for file

Additional file 8 Number of uSNPs with multiple chromosome assignments against similarity score threshold. This file is a figure showing the evolution of the number uSNPs with multiple chromosome assignments as a function of the similarity score threshold. Click here for file
