# Supplementary material for: High-resolution autosomal radiation hybrid maps of the pig genome and their contribution to the genome sequence assembly
Source: BMC Genomics. 2012 Nov 15;13:585. doi: 10.1186/1471-2164-13-585 (PMC3499281; doi:10.1186/1471-2164-13-585)
Supplement: Additional file 3 — Detailed comparison of RH maps with the build9 assembly and with the build10 assembly. This file contains comprehensive pictures comparing (i) the build9 draft assembly and the RH maps and (ii) the pig genome sequence build10 and the RH maps. [file 1471-2164-13-585-S3.pdf]

High resolution autosomal radiation hybrid maps of the pig genome and their contribution to the genome sequence assembly. Additional File 3

Bertrand Servin, Thomas Faraut, Nathale Iannuccelli, Diana Zelenica,Denis Milan

SSC1

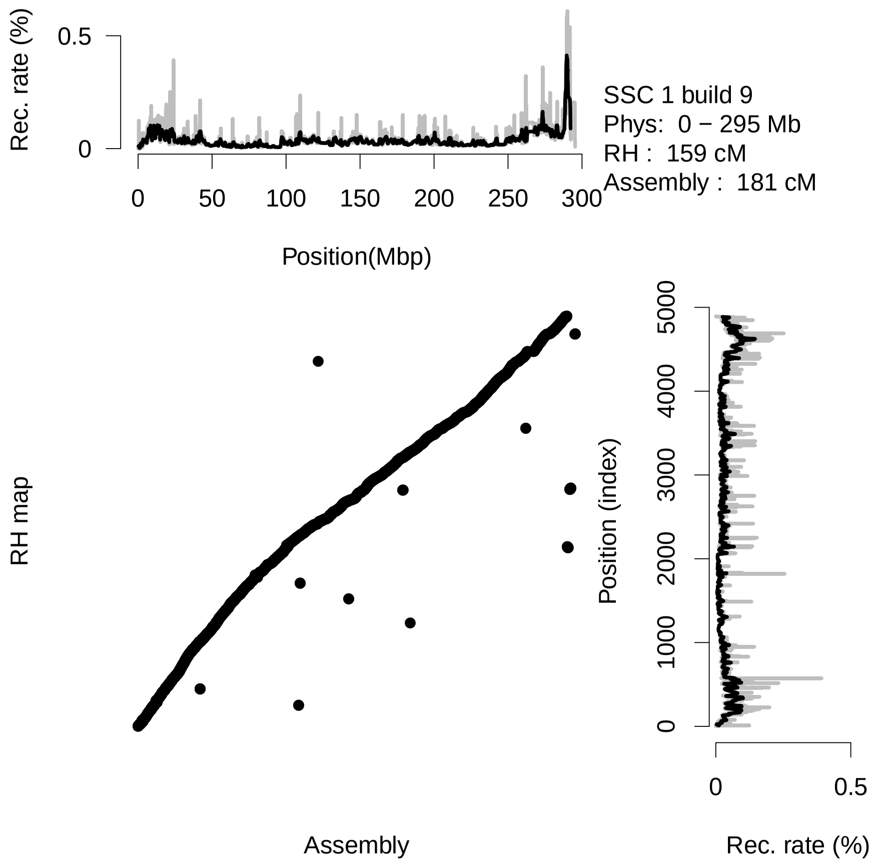
Rec. rate (%)
0.5
0
0
50
100
150
200
250
300
Position(Mbp)
SSC 1 build 9
Phys: 0 − 295 Mb
RH : 159 cM
Assembly : 181 cM
RH map
Assembly
Position (index)
0
1000
2000
3000
4000
5000
0
0.5
Rec. rate (%)

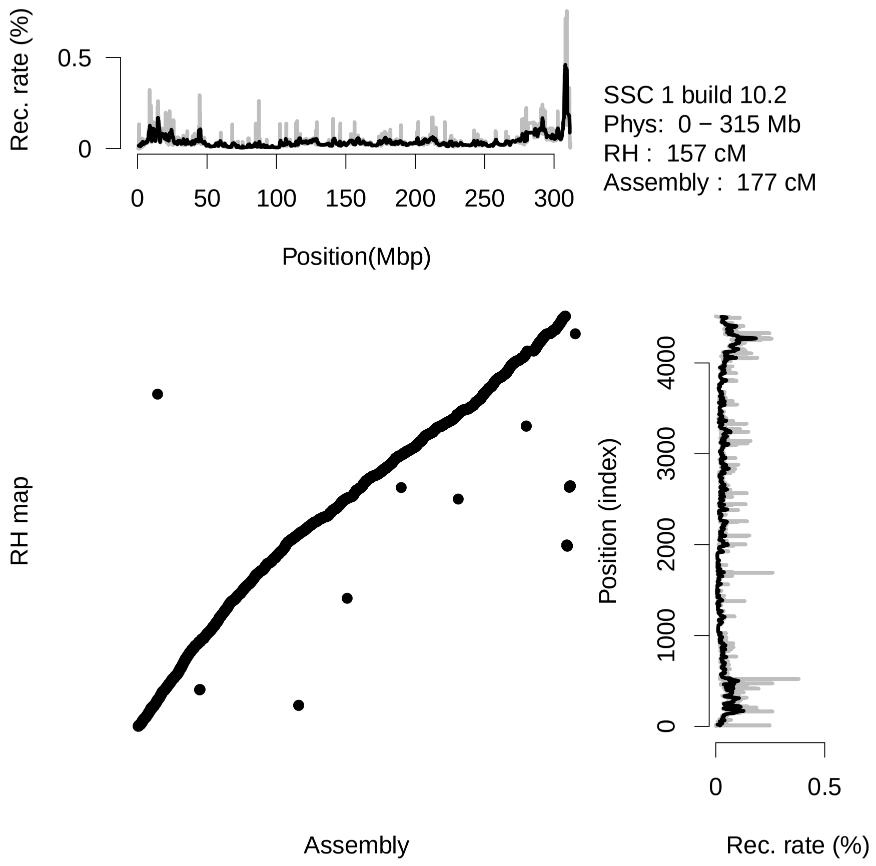
Rec. rate (%)
0.5
0
0
50
100
150
200
250
300
Position(Mbp)
SSC 1 build 10.2
Phys: 0 − 315 Mb
RH : 157 cM
Assembly : 177 cM
RH map
Assembly
Position (index)
0
1000
2000
3000
4000
0
0.5
Rec. rate (%)

SSC2

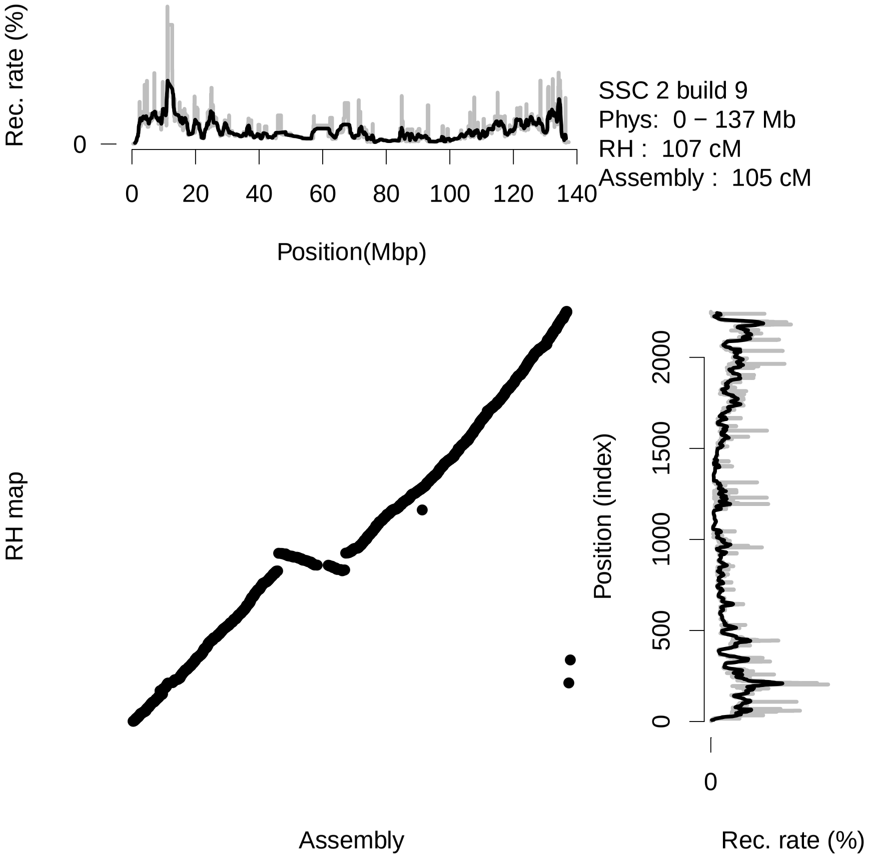
Rec. rate (%)
0
0
20
40
60
80
100
120
140
Position(Mbp)
SSC 2 build 9
Phys: 0 − 137 Mb
RH : 107 cM
Assembly : 105 cM
RH map
Assembly
Position (index)
0
500
1000
1500
2000
0
Rec. rate (%)

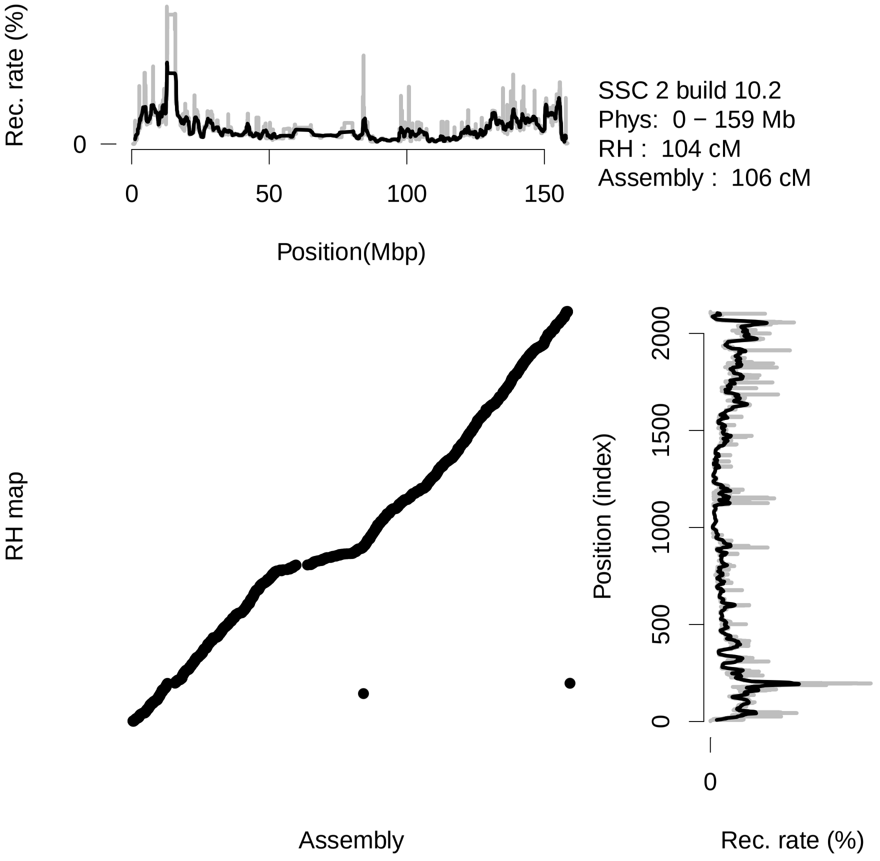
Rec. rate (%)
0
0
50
100
150
Position(Mbp)
SSC 2 build 10.2
Phys: 0 − 159 Mb
RH : 104 cM
Assembly : 106 cM
RH map
Assembly
Position (index)
0
500
1000
1500
2000
0
Rec. rate (%)

SSC3

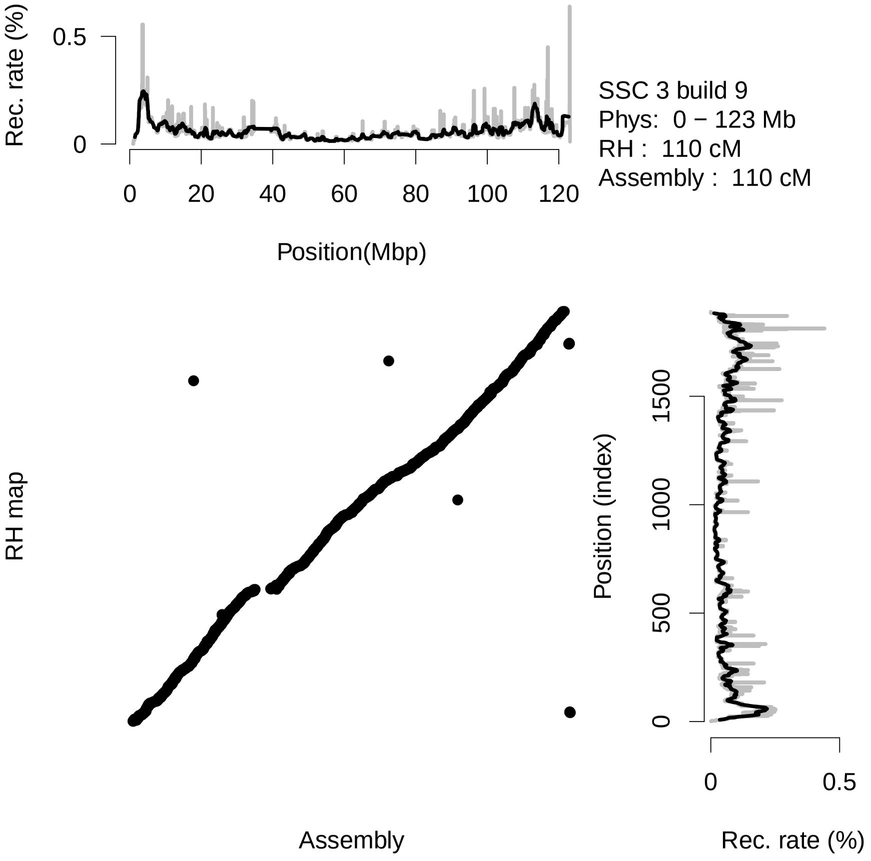

Rec. rate (%)
0.5
0
0
20
40
60
80
100
120
Position(Mbp)
SSC 3 build 9
Phys: 0 − 123 Mb
RH : 110 cM
Assembly : 110 cM
RH map
Assembly
Position (index)
1500
1000
500
0
0
0.5
Rec. rate (%)

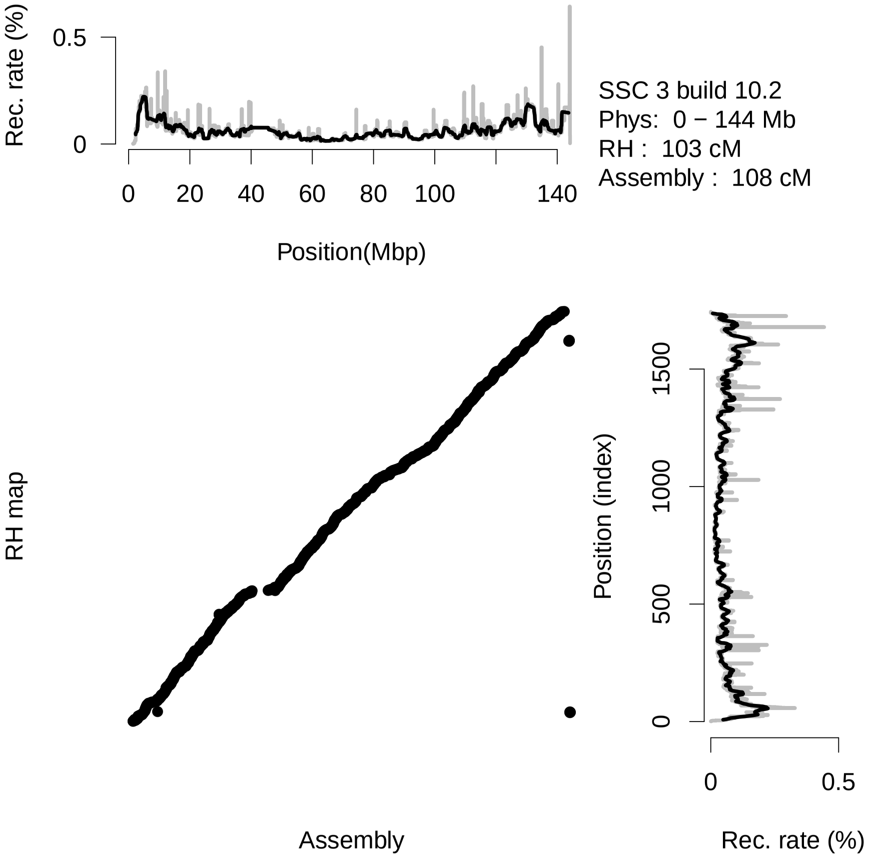

Rec. rate (%)
0.5
0
0
20
40
60
80
100
140
Position(Mbp)
SSC 3 build 10.2
Phys: 0 − 144 Mb
RH : 103 cM
Assembly : 108 cM
RH map
Assembly
Position (index)
1500
1000
500
0
0
0.5
Rec. rate (%)

SSC4

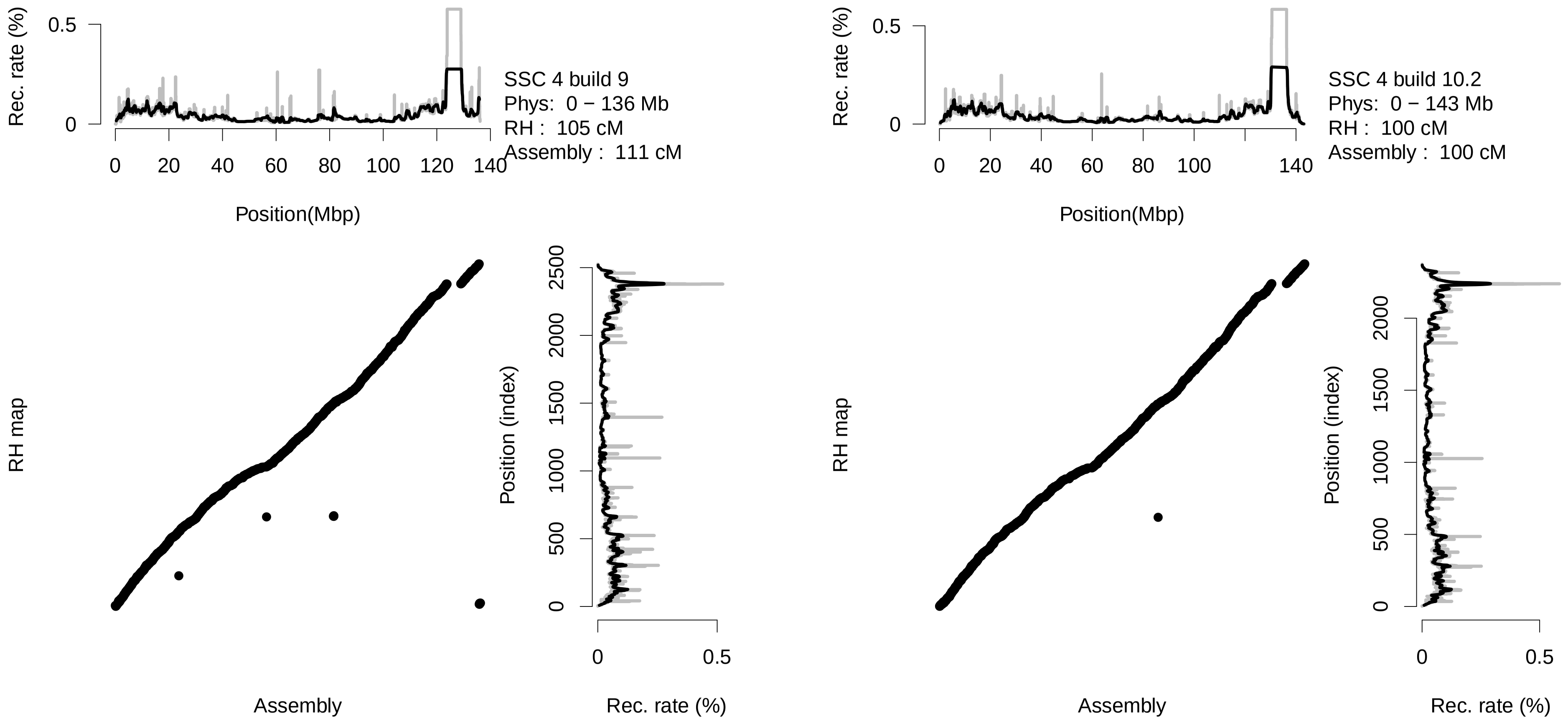

Rec. rate (%)
0.5
0
0
20
40
60
80
100
120
140
Position(Mbp)
SSC 4 build 9
Phys: 0 − 136 Mb
RH : 105 cM
Assembly : 111 cM
RH map
Assembly
Position (index)
2500
2000
1500
1000
500
0
0
0.5
Rec. rate (%)
Rec. rate (%)
0.5
0
0
20
40
60
80
100
140
Position(Mbp)
SSC 4 build 10.2
Phys: 0 − 143 Mb
RH : 100 cM
Assembly : 100 cM
RH map
Assembly
Position (index)
2000
1500
1000
500
0
0
0.5
Rec. rate (%)

SSC5

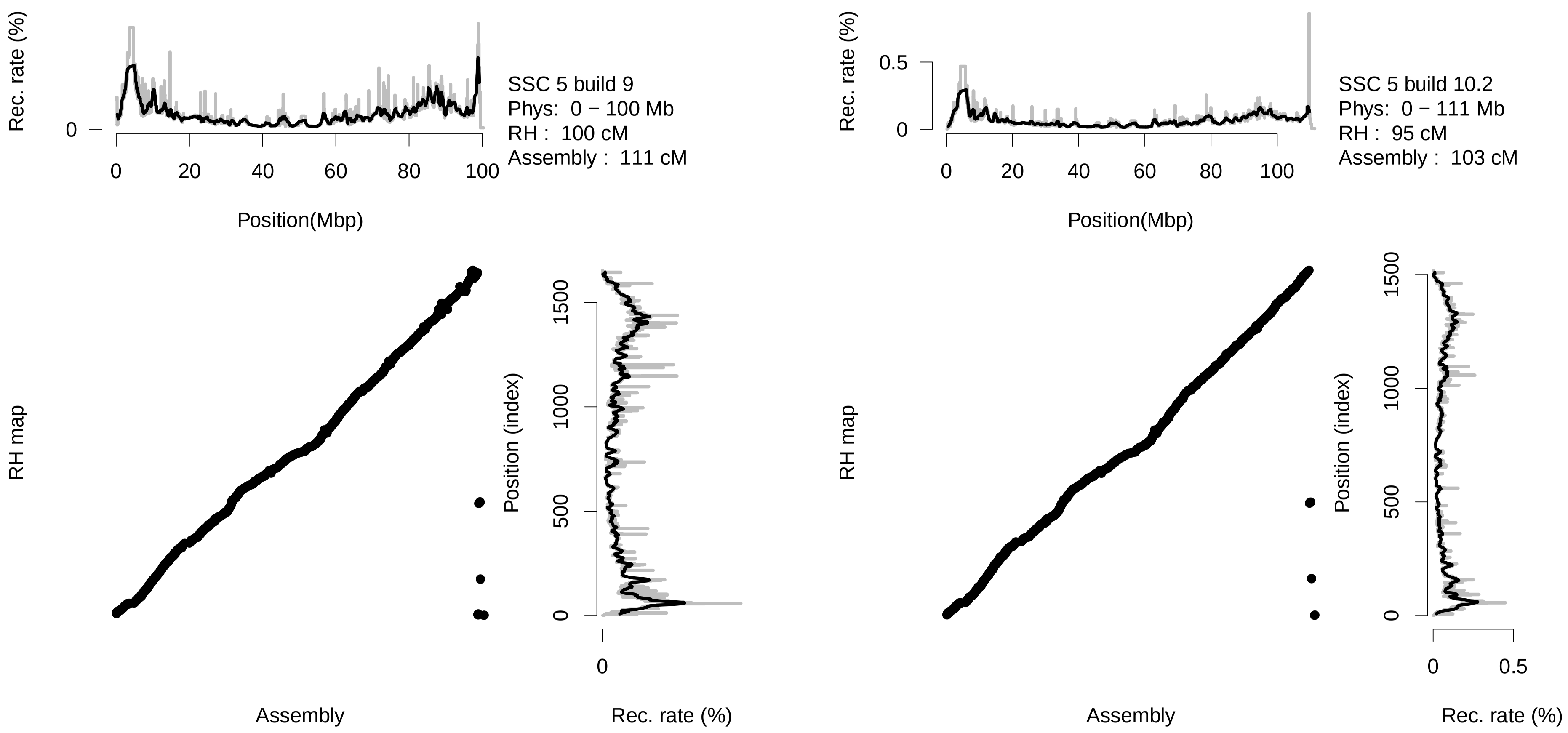

SSC 5 build 9
Phys: 0 − 100 Mb
RH : 100 cM
Assembly : 111 cM
Rec. rate (%)
Position(Mbp)
RH map
Assembly
Position (index)
SSC 5 build 10.2
Phys: 0 − 111 Mb
RH : 95 cM
Assembly : 103 cM

SSC5 Zoom 1

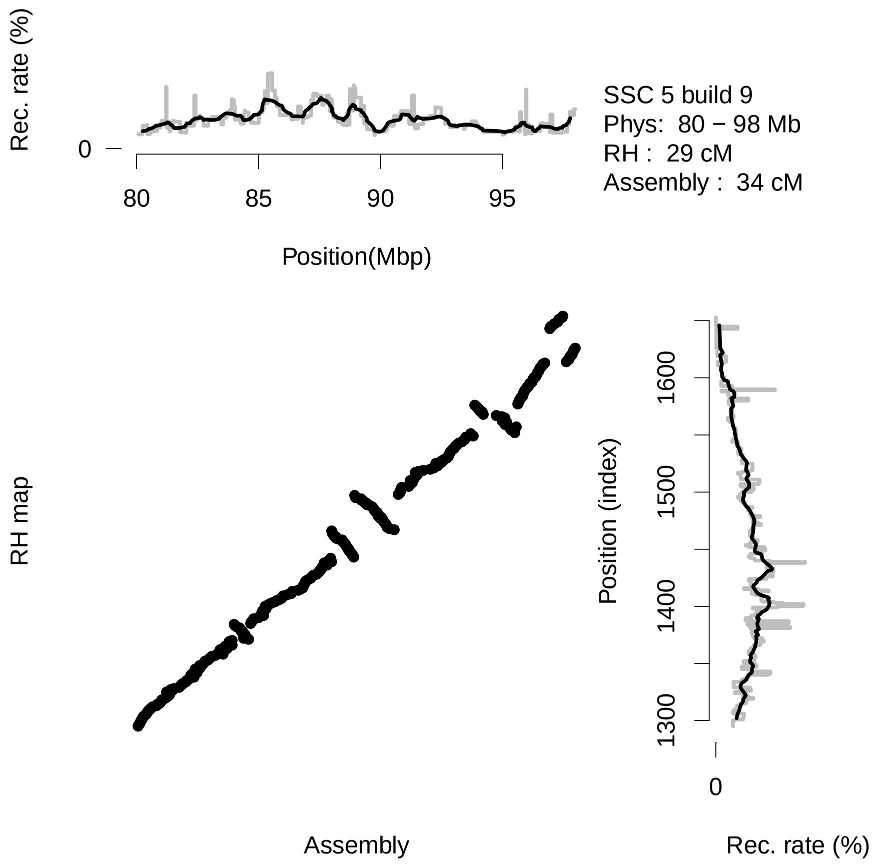
Rec. rate (%)
0
80
85
90
95
Position(Mbp)
SSC 5 build 9
Phys: 80 − 98 Mb
RH : 29 cM
Assembly : 34 cM
RH map
Assembly
Position (index)
1300
1400
1500
1600
Rec. rate (%)

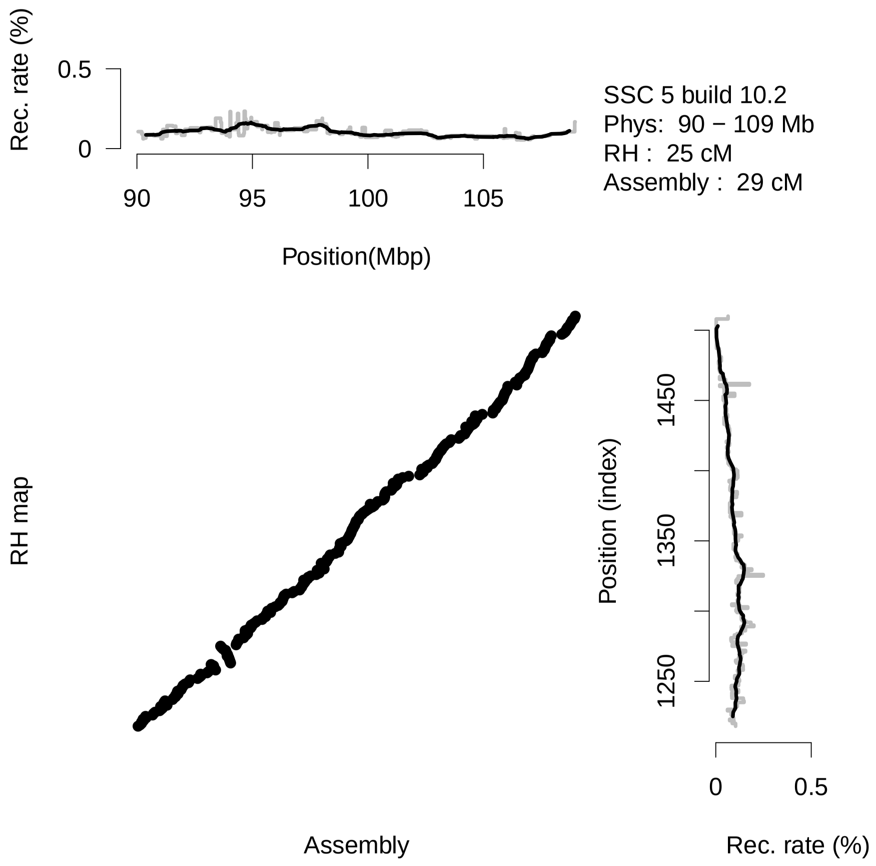
Rec. rate (%)
0.5
0
90
95
100
105
Position(Mbp)
SSC 5 build 10.2
Phys: 90 − 109 Mb
RH : 25 cM
Assembly : 29 cM
RH map
Assembly
Position (index)
1250
1350
1450
0.5
Rec. rate (%)

SSC6

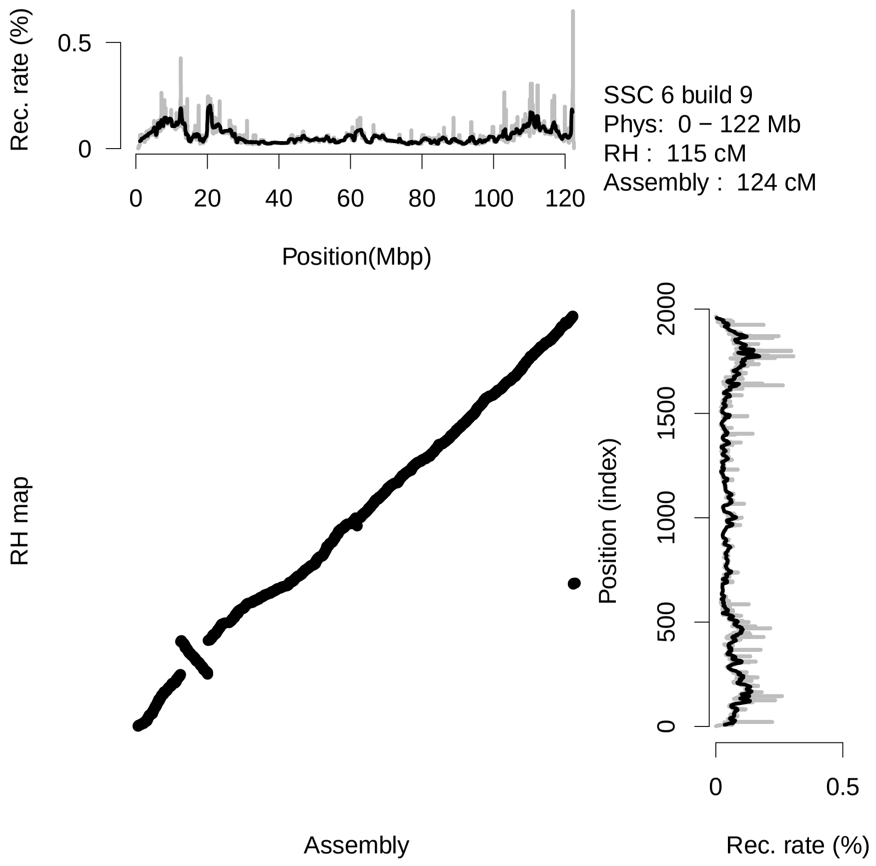
Rec. rate (%)
0.5
0
0
20
40
60
80
100
120
Position(Mbp)
SSC 6 build 9
Phys: 0 − 122 Mb
RH : 115 cM
Assembly : 124 cM
RH map
Assembly
Position (index)
2000
1500
1000
500
0
0.5
Rec. rate (%)

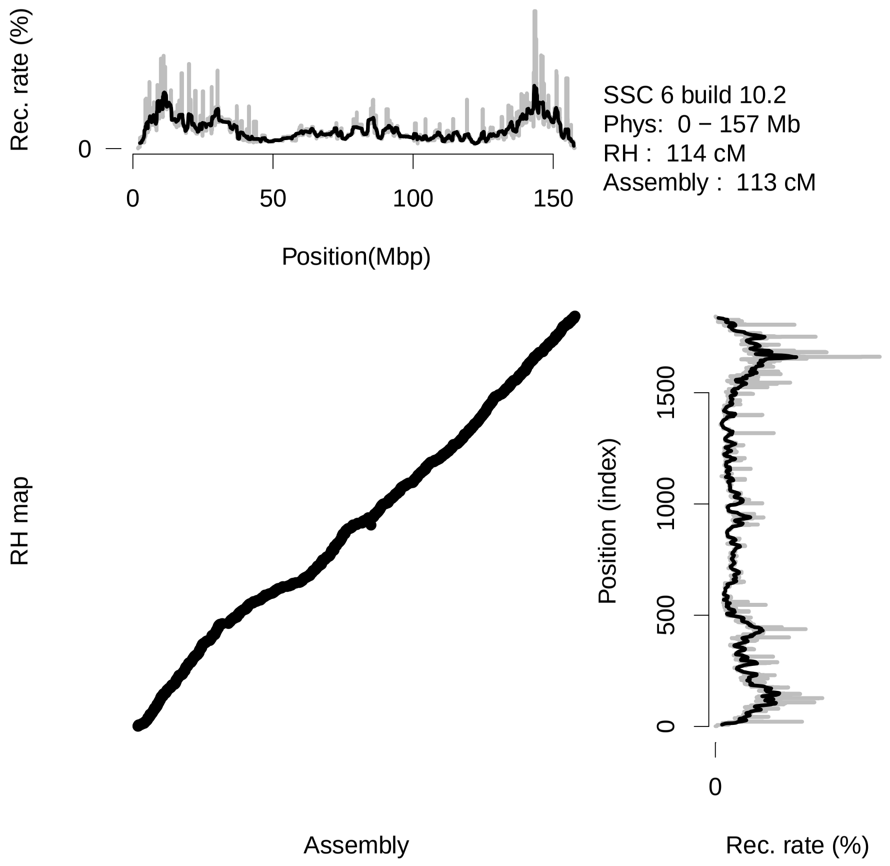
Rec. rate (%)
0
0
50
100
150
Position(Mbp)
SSC 6 build 10.2
Phys: 0 − 157 Mb
RH : 114 cM
Assembly : 113 cM
RH map
Assembly
Position (index)
1500
1000
500
0
Rec. rate (%)

SSC6 Zoom 1

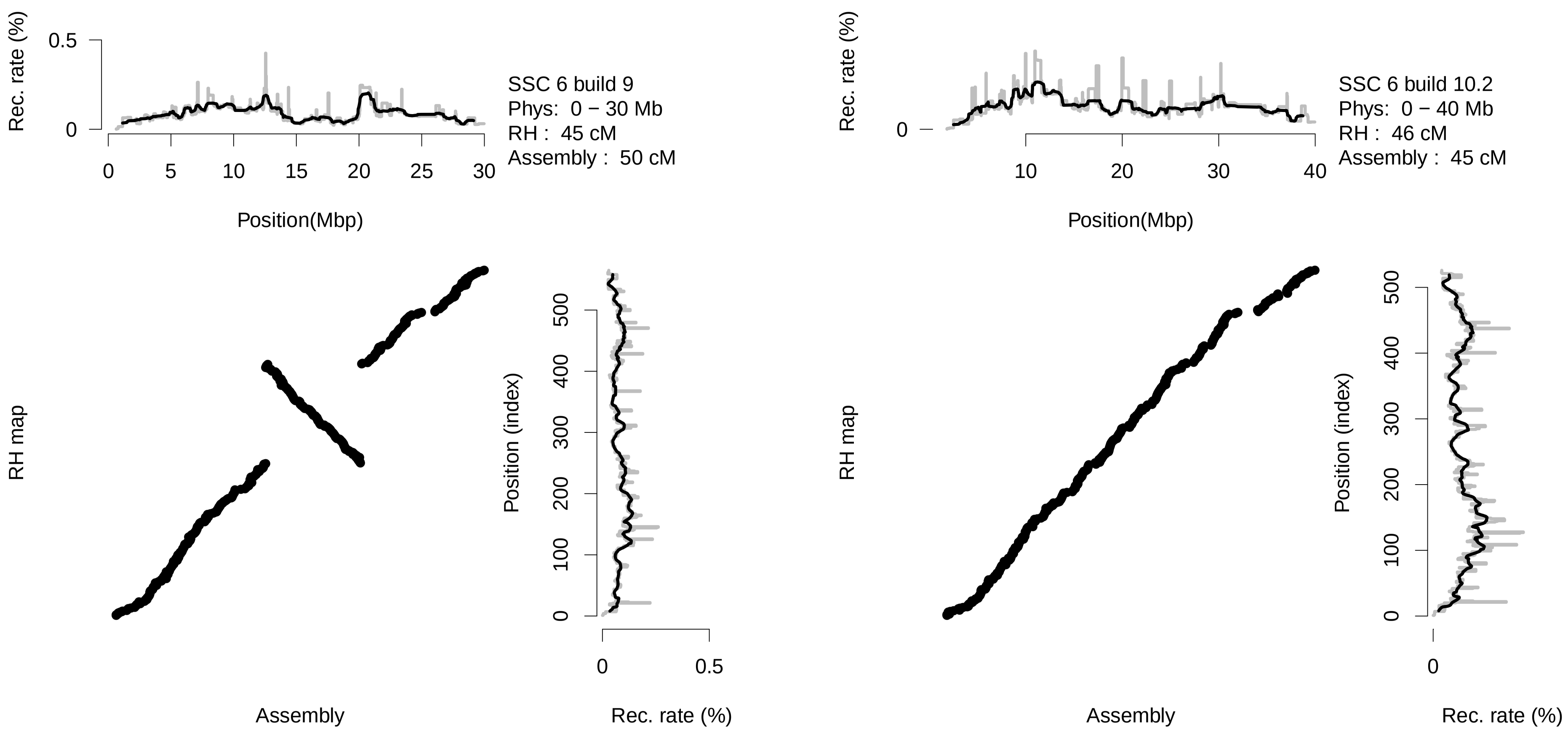
SSC 6 build 9
Phys: 0 − 30 Mb
RH : 45 cM
Assembly : 50 cM
Rec. rate (%)
Position(Mbp)
RH map
Assembly
Position (index)
SSC 6 build 10.2
Phys: 0 − 40 Mb
RH : 46 cM
Assembly : 45 cM

SSC7

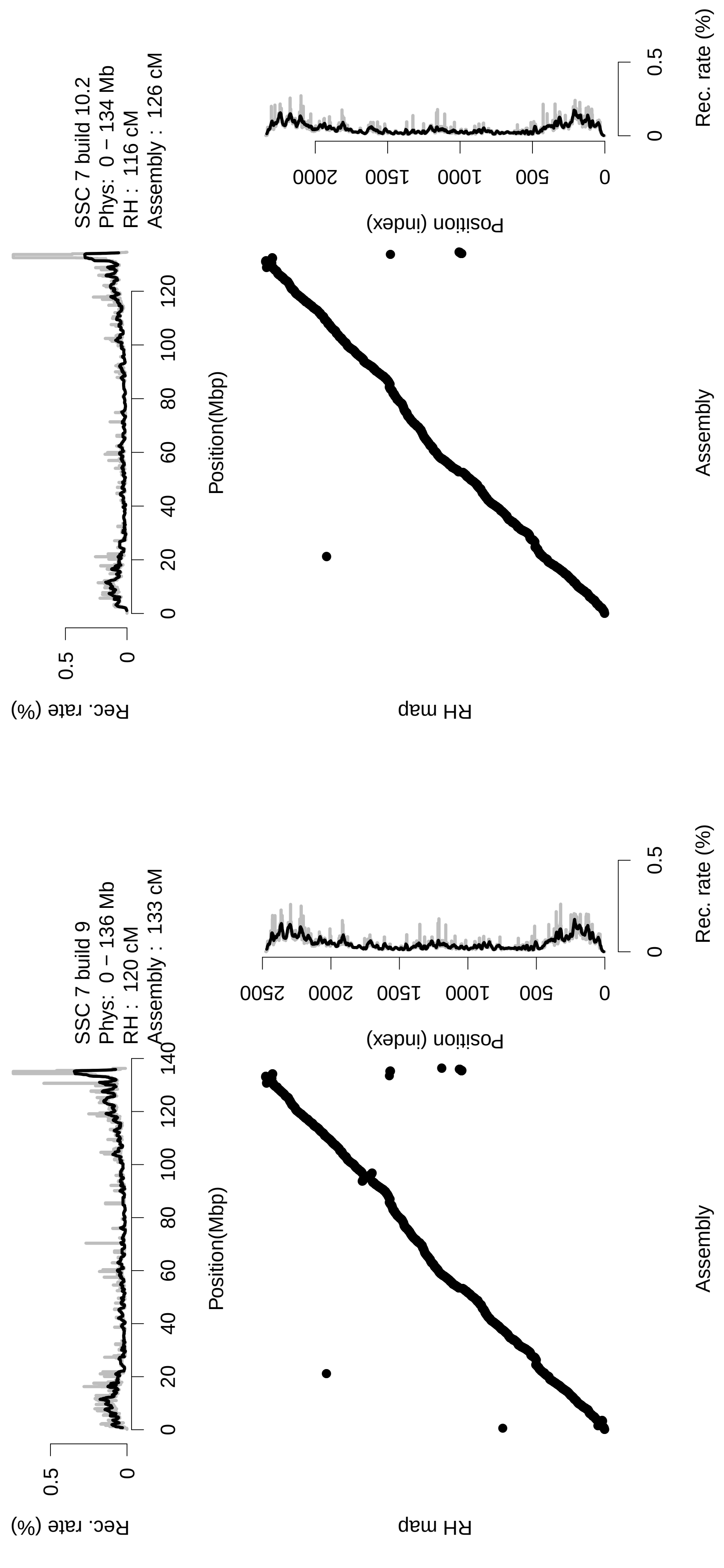
SSC 7 build 10.2
Phys: 0 – 134 Mb
RH : 116 cM
Assembly : 126 cM
SSC 7 build 9
Phys: 0 – 136 Mb
RH : 120 cM
Assembly : 133 cM
Position(Mbp)
Rec. rate (%)
Position (index)
RH map
Assembly

SSC7 Zoom 1

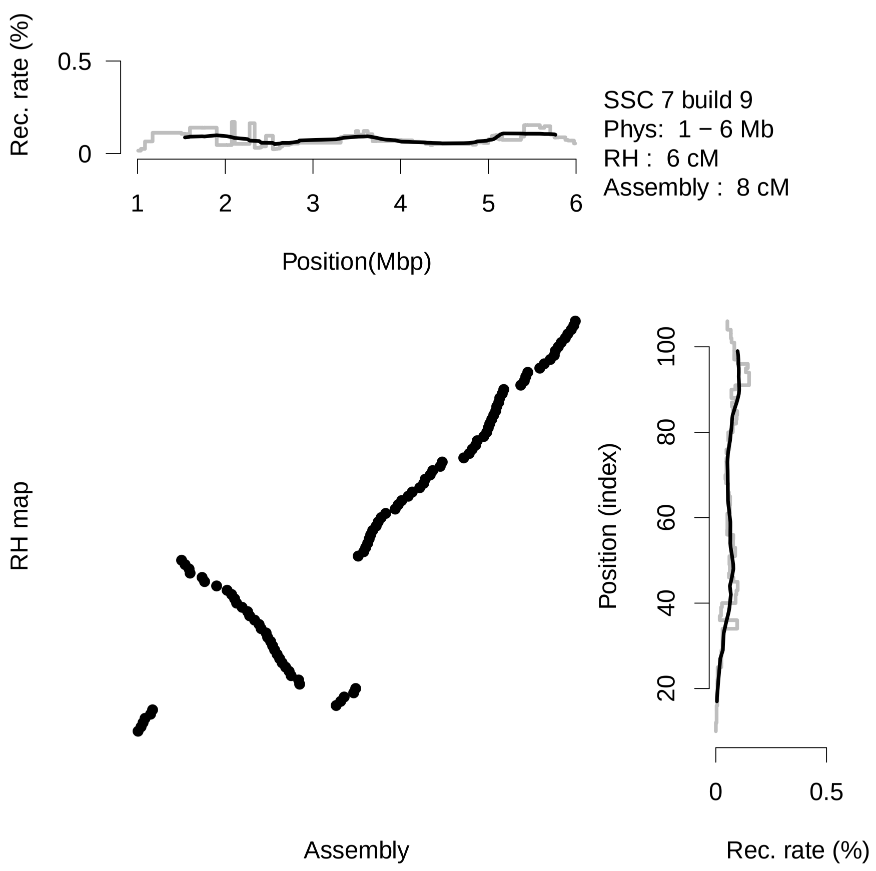

Rec. rate (%)
0.5
0
SSC 7 build 9
Phys: 1 − 6 Mb
RH : 6 cM
Assembly : 8 cM
1
2
3
4
5
6
Position(Mbp)
RH map
Position (index)
100
80
60
40
20
0
0.5
Assembly
Rec. rate (%)

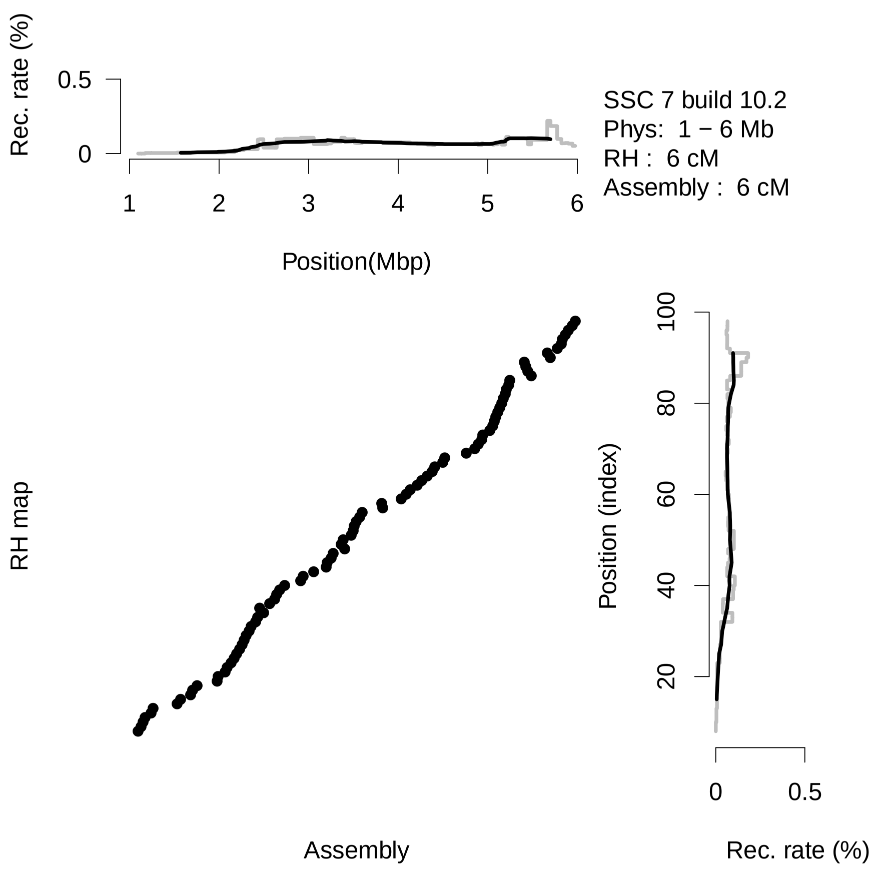

Rec. rate (%)
0.5
0
SSC 7 build 10.2
Phys: 1 − 6 Mb
RH : 6 cM
Assembly : 6 cM
1
2
3
4
5
6
Position(Mbp)
RH map
Position (index)
100
80
60
40
20
0
0.5
Assembly
Rec. rate (%)

SSC7 Zoom 2

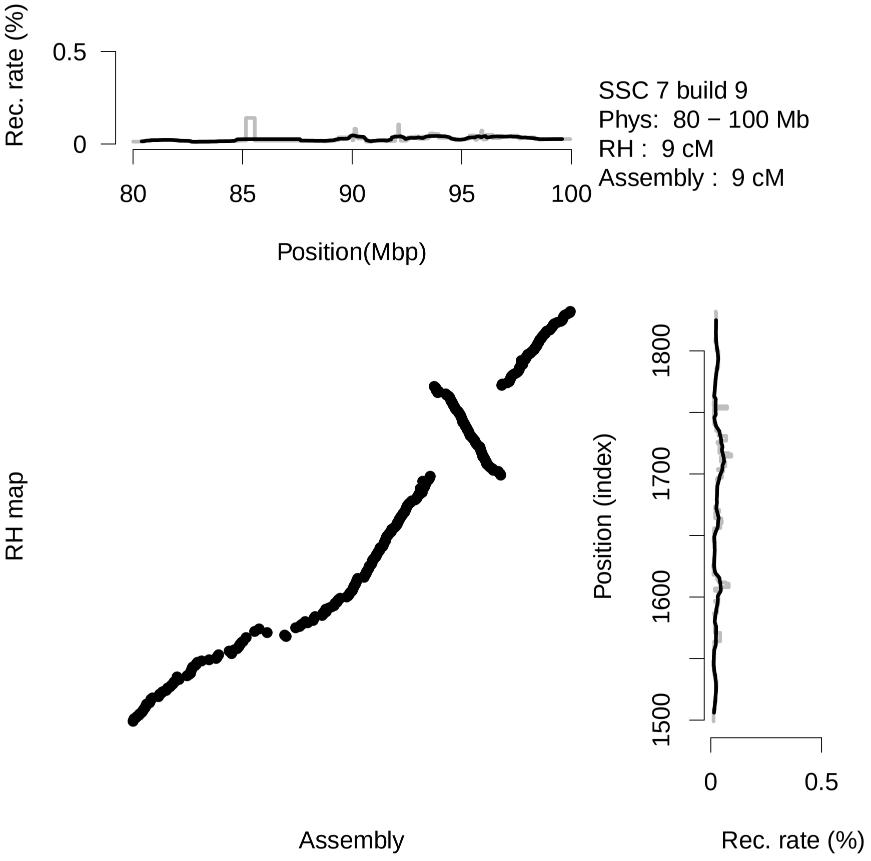
SSC 7 build 9
Phys: 80 − 100 Mb
RH : 9 cM
Assembly : 9 cM
Rec. rate (%)
Position(Mbp)
RH map
Assembly
Position (index)
80
85
90
95
100
1500
1600
1700
1800
0
0.5

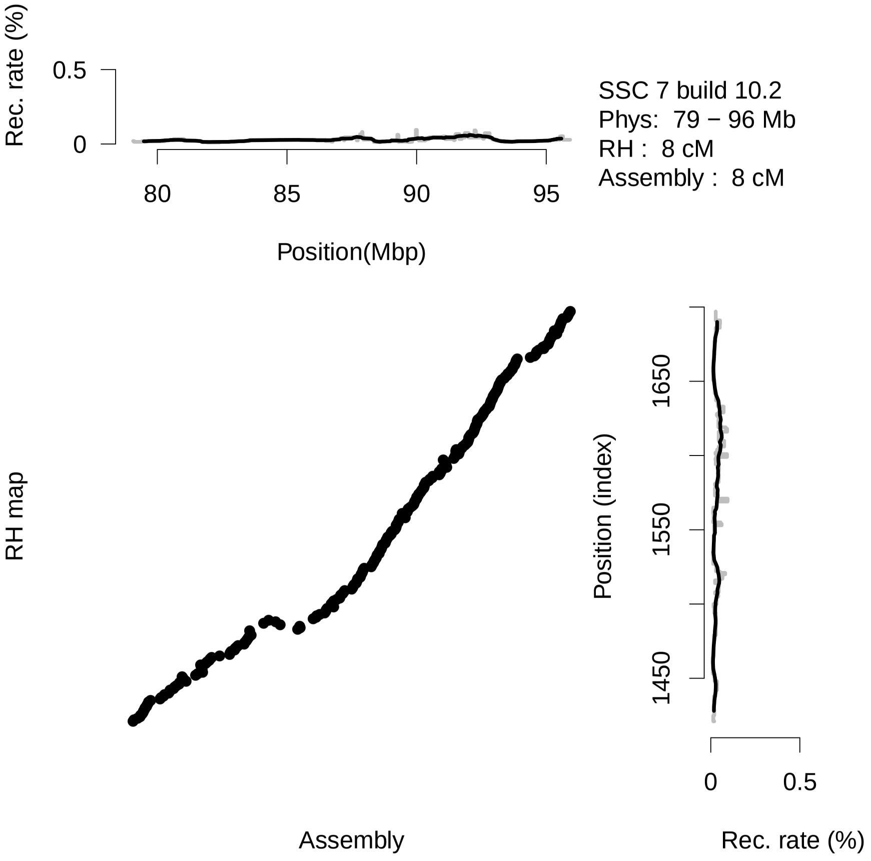
SSC 7 build 10.2
Phys: 79 − 96 Mb
RH : 8 cM
Assembly : 8 cM
Rec. rate (%)
Position(Mbp)
RH map
Assembly
Position (index)
80
85
90
95
1450
1550
1650
0
0.5

SSC7 Zoom 3

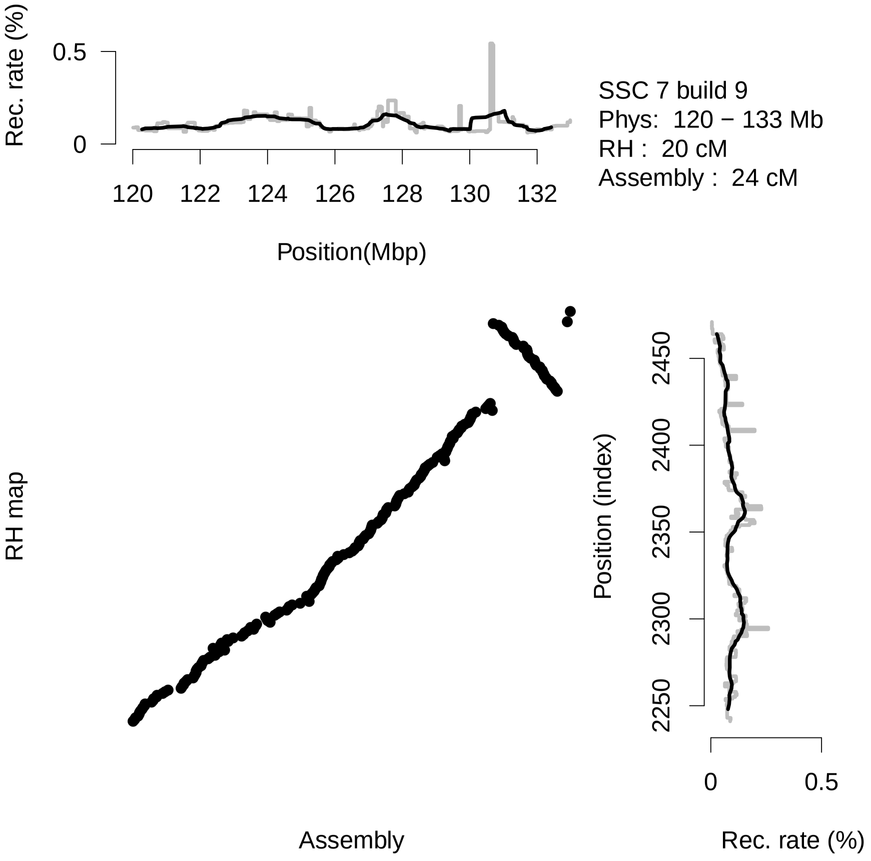

Rec. rate (%)
0.5
0
120
122
124
126
128
130
132
Position(Mbp)
SSC 7 build 9
Phys: 120 − 133 Mb
RH : 20 cM
Assembly : 24 cM
RH map
Assembly
Position (index)
2450
2400
2350
2300
2250
0
0.5
Rec. rate (%)

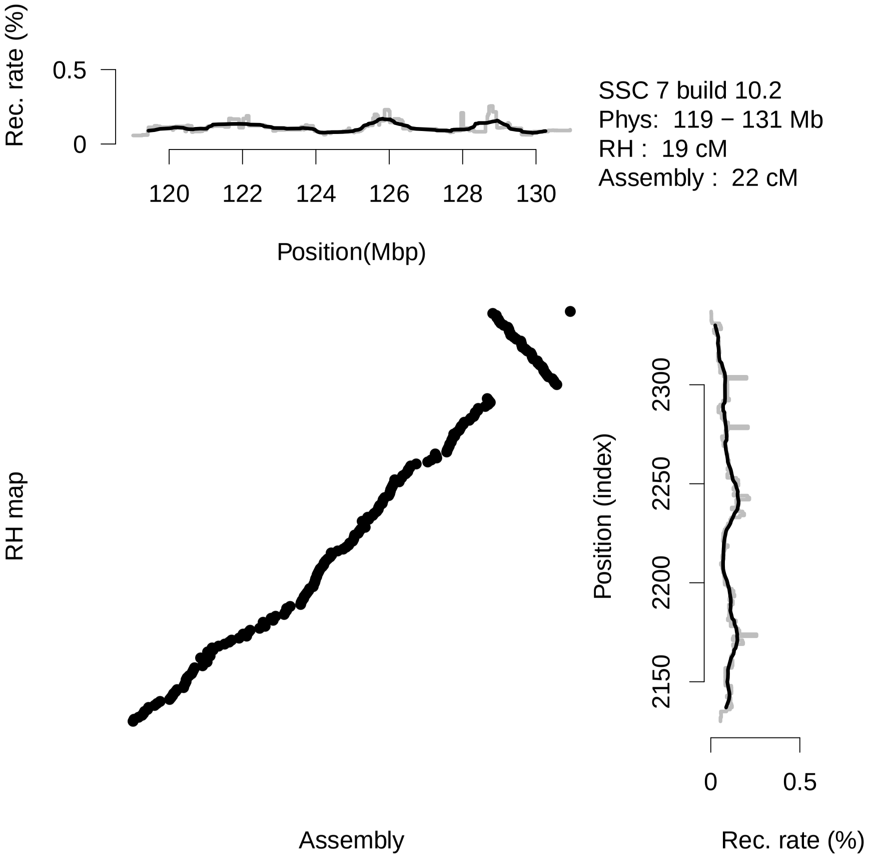

Rec. rate (%)
0.5
0
120
122
124
126
128
130
Position(Mbp)
SSC 7 build 10.2
Phys: 119 − 131 Mb
RH : 19 cM
Assembly : 22 cM
RH map
Assembly
Position (index)
2300
2250
2200
2150
0
0.5
Rec. rate (%)

SSC8

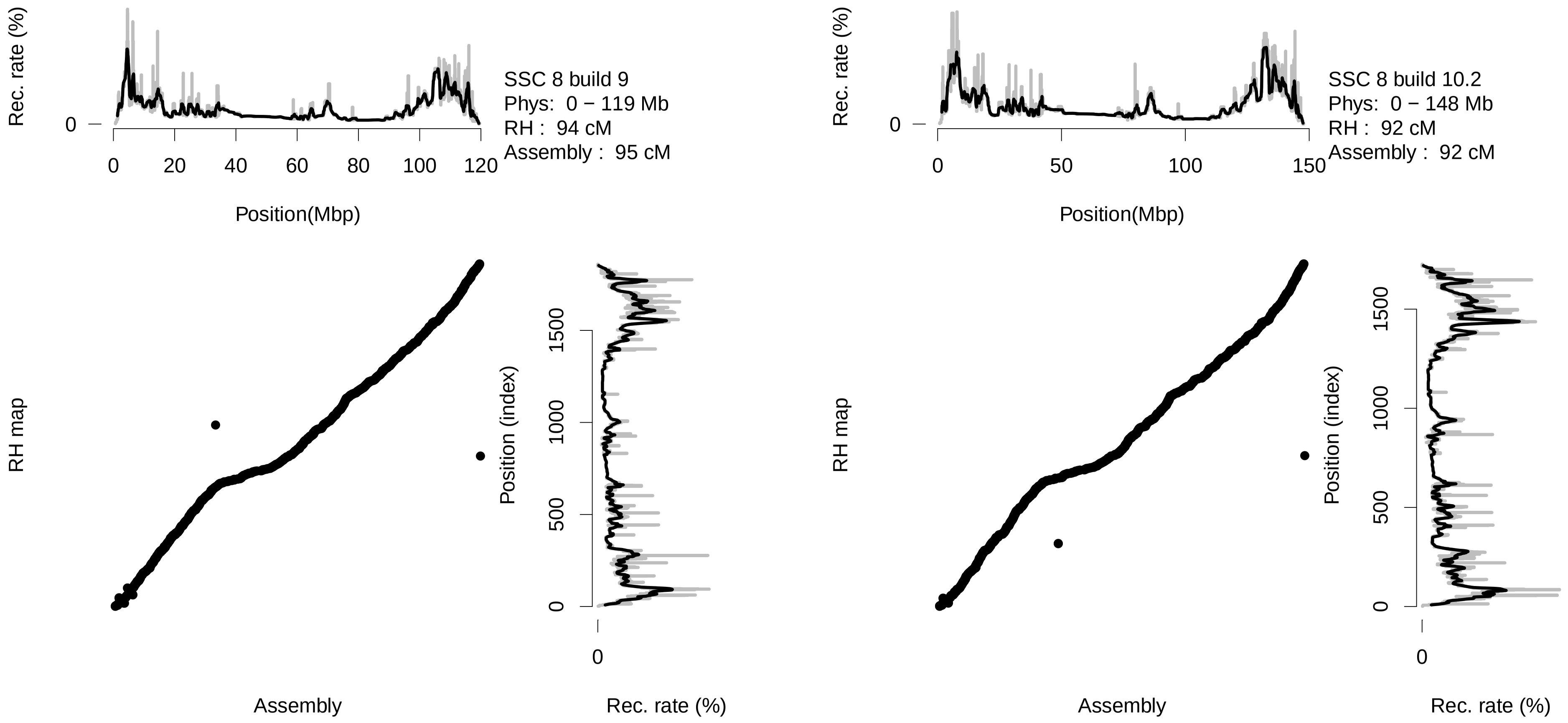

Rec. rate (%)
Position(Mbp)
SSC 8 build 9
Phys: 0 − 119 Mb
RH : 94 cM
Assembly : 95 cM
RH map
Assembly
Position (index)
Rec. rate (%)
SSC 8 build 10.2
Phys: 0 − 148 Mb
RH : 92 cM
Assembly : 92 cM

SSC8 Zoom 1

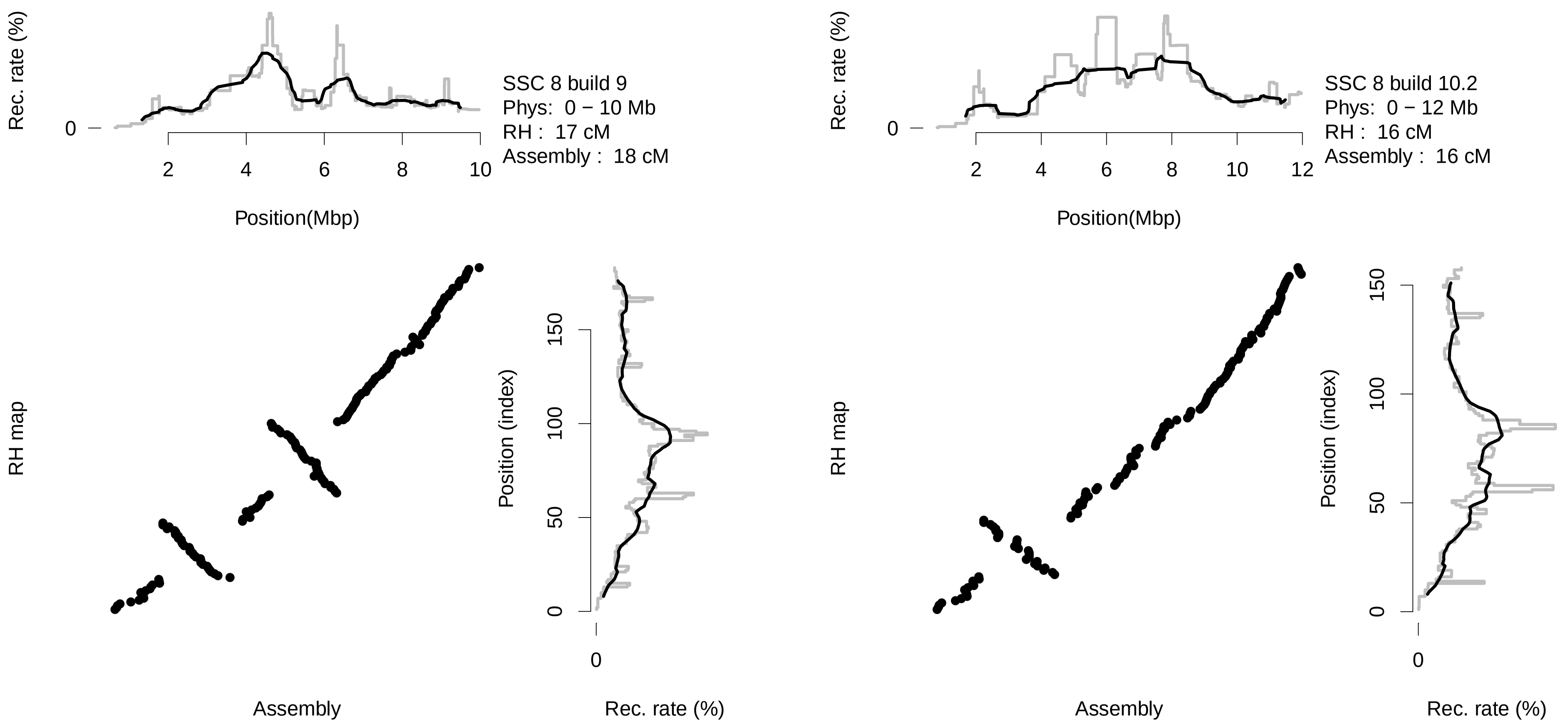

Rec. rate (%)
Position(Mbp)
SSC 8 build 9
Phys: 0 − 10 Mb
RH : 17 cM
Assembly : 18 cM
RH map
Assembly
Position (index)
Rec. rate (%)
SSC 8 build 10.2
Phys: 0 − 12 Mb
RH : 16 cM
Assembly : 16 cM

SSC9

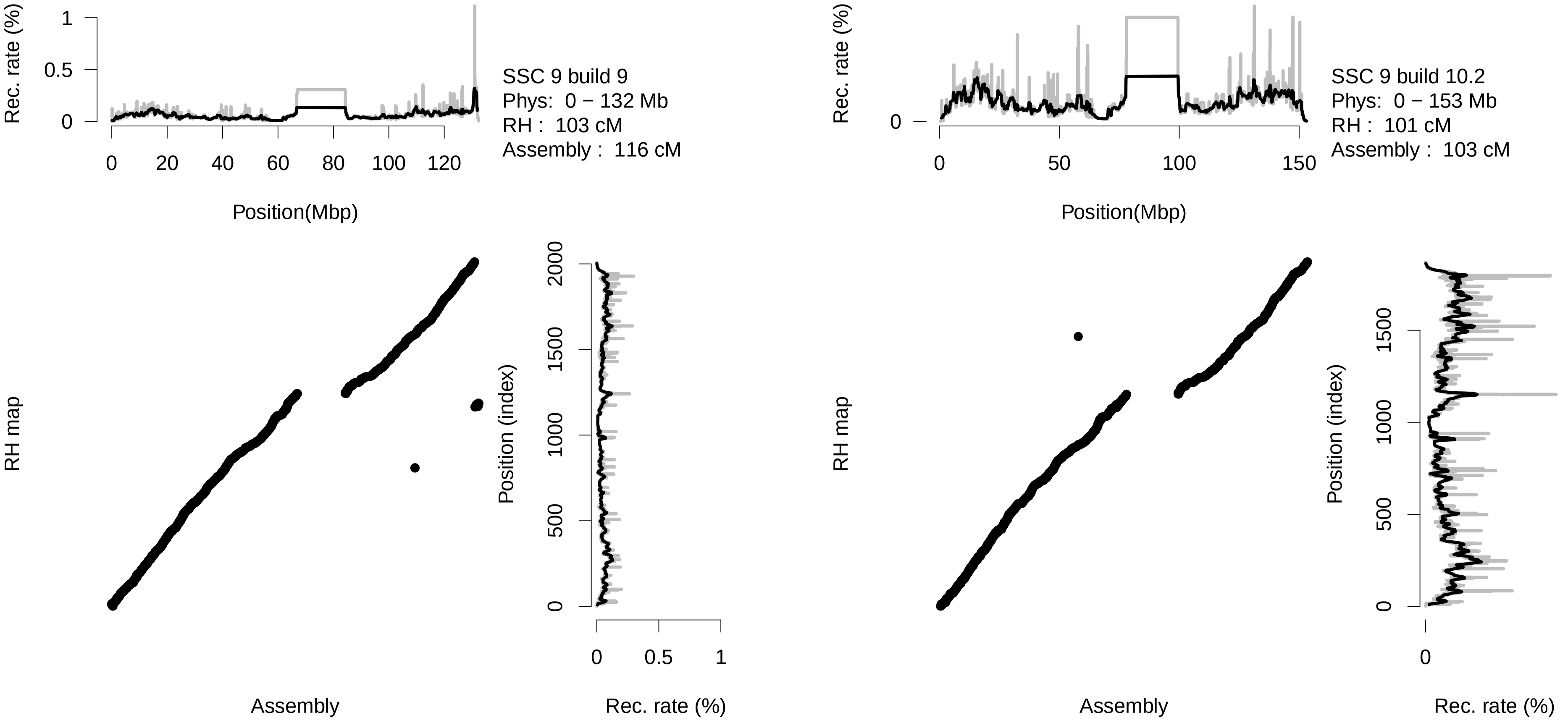

Rec. rate (%)
1
0.5
0
0
20
40
60
80
100
120
Position(Mbp)
SSC 9 build 9
Phys: 0 − 132 Mb
RH : 103 cM
Assembly : 116 cM
RH map
Assembly
Position (index)
0
500
1000
1500
2000
0
0.5
1
Rec. rate (%)
Rec. rate (%)
0
0
50
100
150
Position(Mbp)
SSC 9 build 10.2
Phys: 0 − 153 Mb
RH : 101 cM
Assembly : 103 cM
RH map
Assembly
Position (index)
0
500
1000
1500
0
Rec. rate (%)

SSC9 Zoom 1

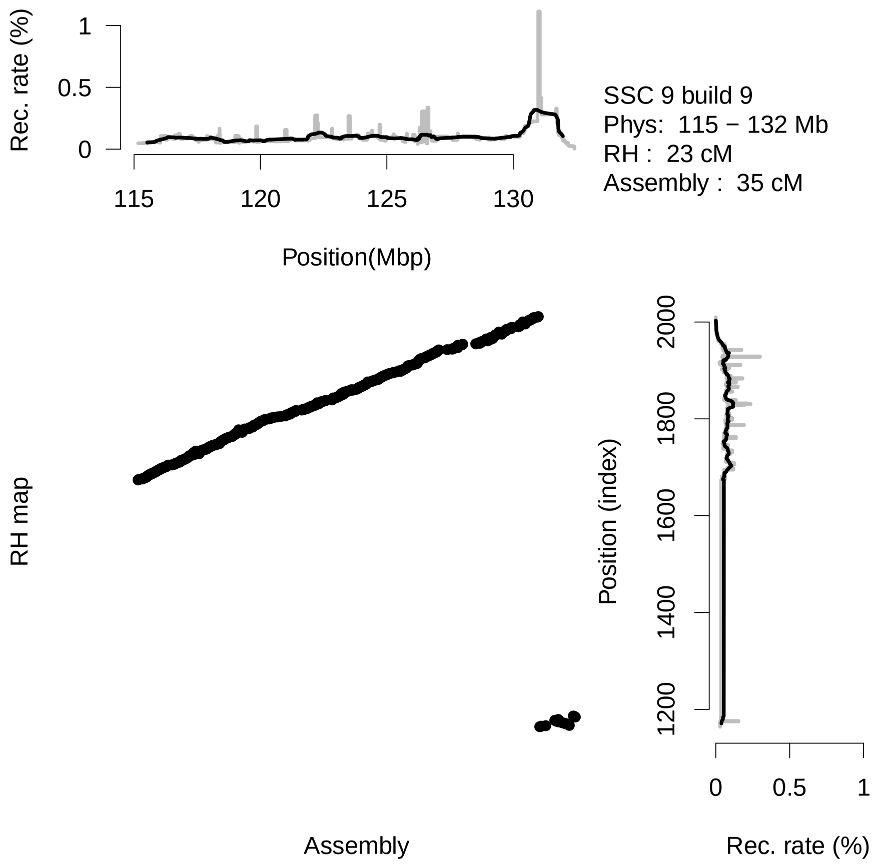
Rec. rate (%)
1
0.5
0
115
120
125
130
Position(Mbp)
SSC 9 build 9
Phys: 115 − 132 Mb
RH : 23 cM
Assembly : 35 cM
RH map
Assembly
Position (index)
2000
1800
1600
1400
1200
0
0.5
1
Rec. rate (%)

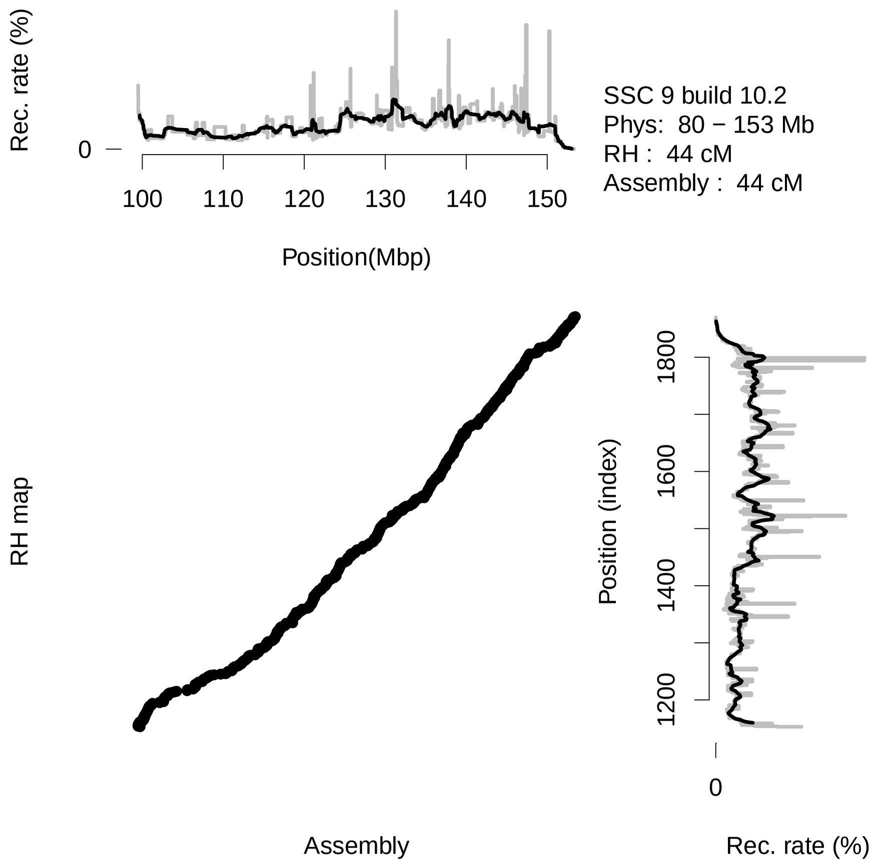
Rec. rate (%)
0
100
110
120
130
140
150
Position(Mbp)
SSC 9 build 10.2
Phys: 80 − 153 Mb
RH : 44 cM
Assembly : 44 cM
RH map
Assembly
Position (index)
1800
1600
1400
1200
0
Rec. rate (%)

SSC10

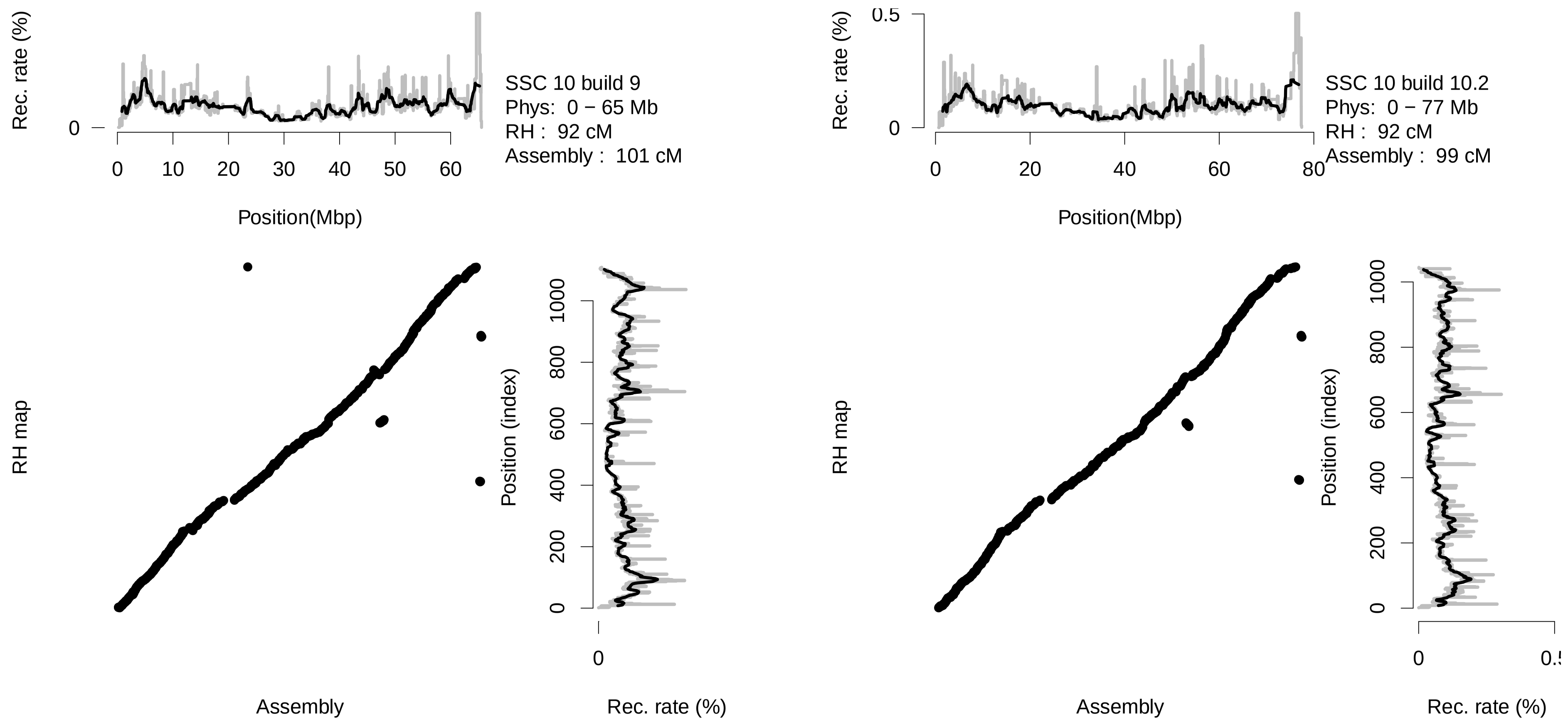

Rec. rate (%)
Position(Mbp)
SSC 10 build 9
Phys: 0 − 65 Mb
RH : 92 cM
Assembly : 101 cM
RH map
Assembly
Position (index)
Rec. rate (%)
SSC 10 build 10.2
Phys: 0 − 77 Mb
RH : 92 cM
Assembly : 99 cM

SSC10 Zoom 1

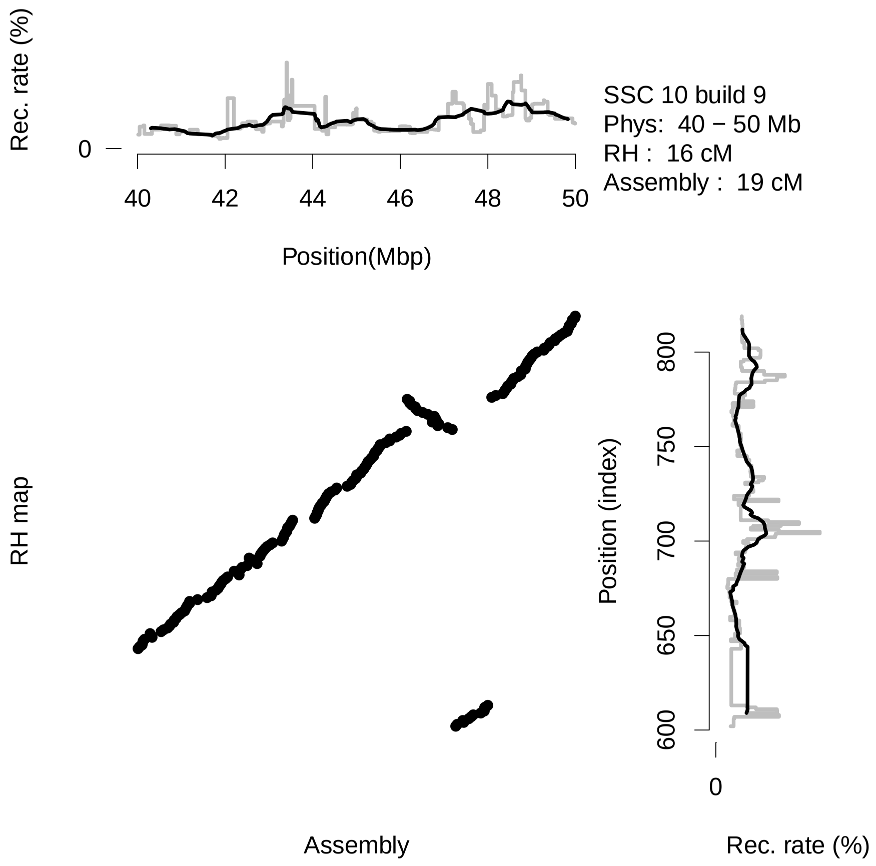
Rec. rate (%)
0
40
42
44
46
48
50
Position(Mbp)
SSC 10 build 9
Phys: 40 − 50 Mb
RH : 16 cM
Assembly : 19 cM
RH map
Assembly
Position (index)
600
650
700
750
800
0
Rec. rate (%)

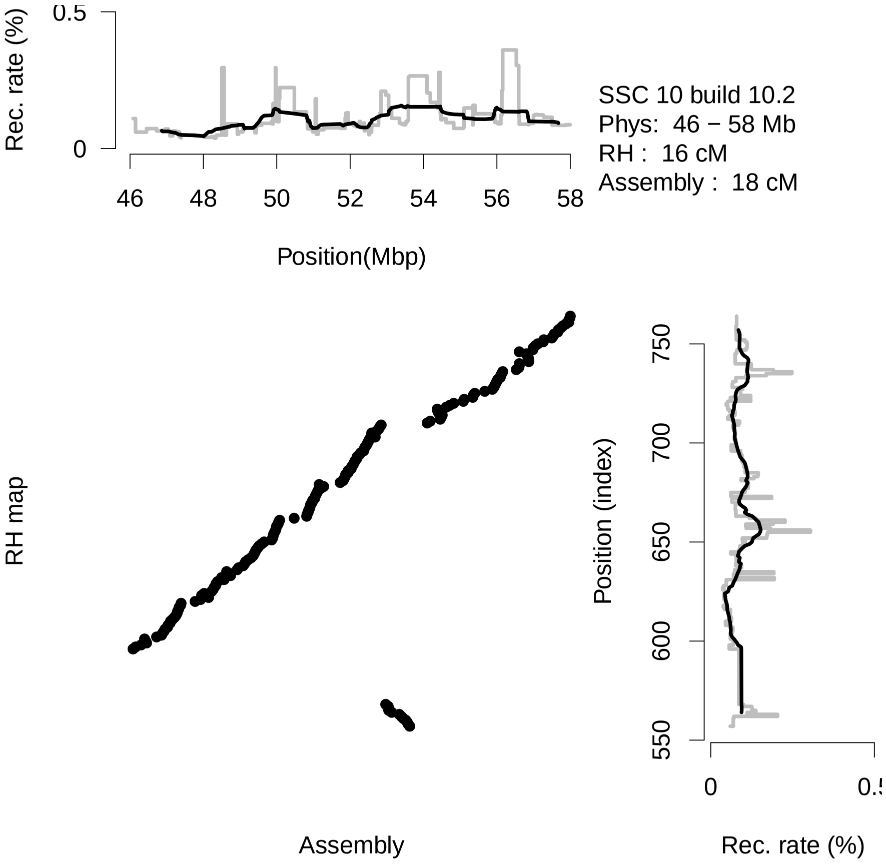
Rec. rate (%)
0.5
0
46
48
50
52
54
56
58
Position(Mbp)
SSC 10 build 10.2
Phys: 46 − 58 Mb
RH : 16 cM
Assembly : 18 cM
RH map
Assembly
Position (index)
550
600
650
700
750
0
Rec. rate (%)

SSC11

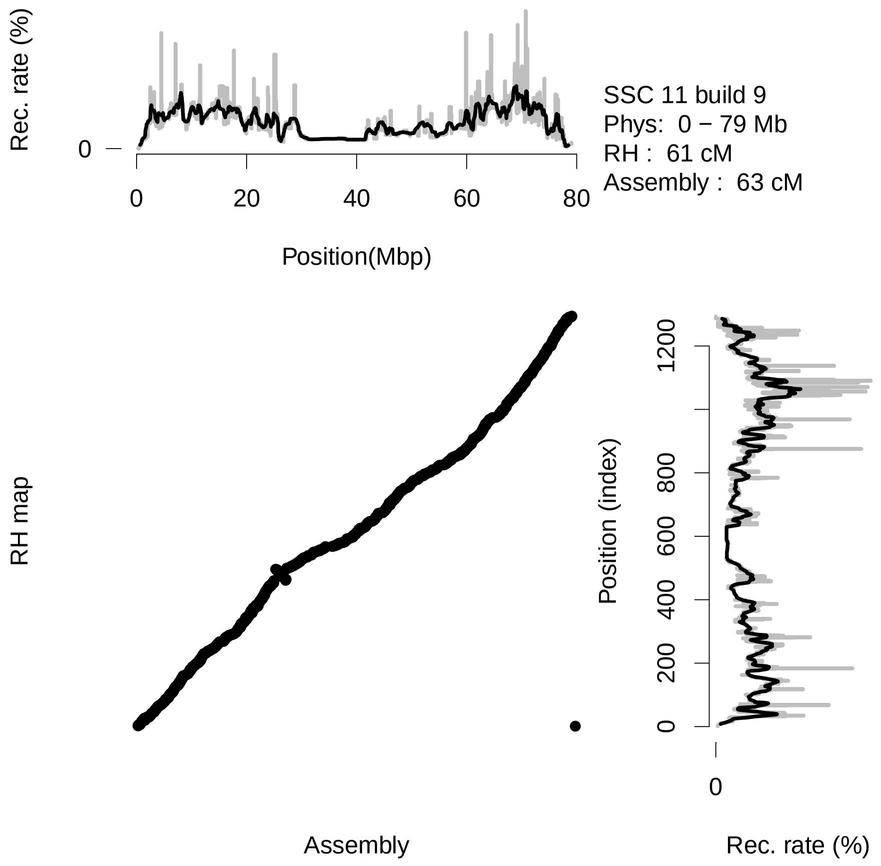
Rec. rate (%)
0
0
20
40
60
80
Position(Mbp)
SSC 11 build 9
Phys: 0 − 79 Mb
RH : 61 cM
Assembly : 63 cM
RH map
Assembly
Position (index)
0
200
400
600
800
1200
0
Rec. rate (%)

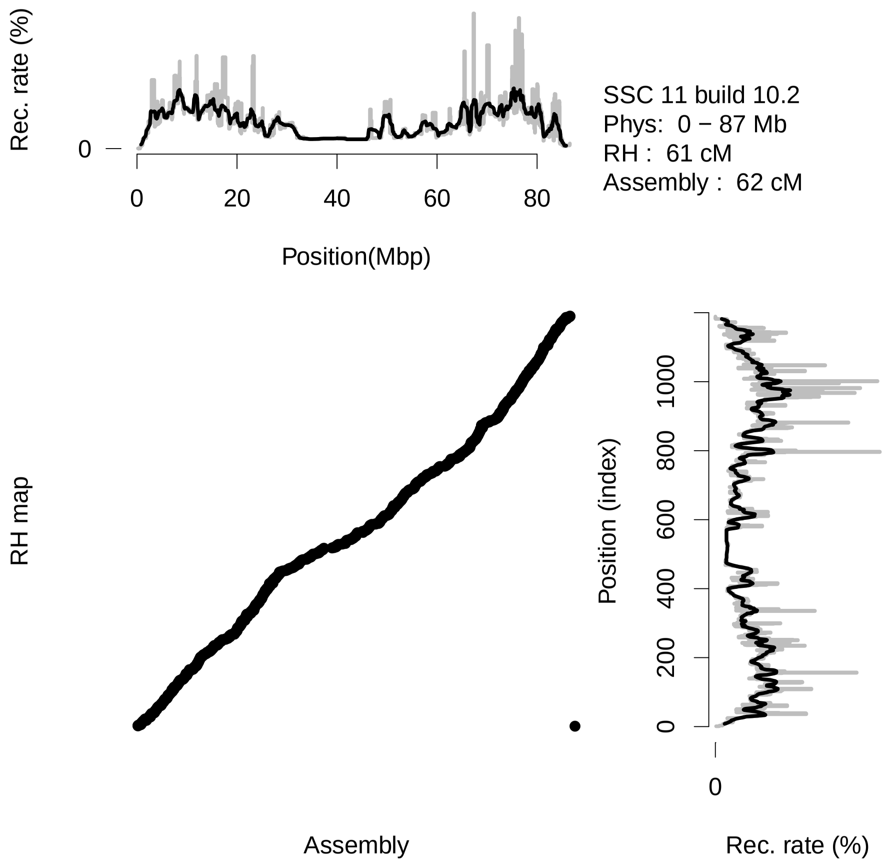
Rec. rate (%)
0
0
20
40
60
80
Position(Mbp)
SSC 11 build 10.2
Phys: 0 − 87 Mb
RH : 61 cM
Assembly : 62 cM
RH map
Assembly
Position (index)
0
200
400
600
800
1000
0
Rec. rate (%)

SSC11 Zoom 1

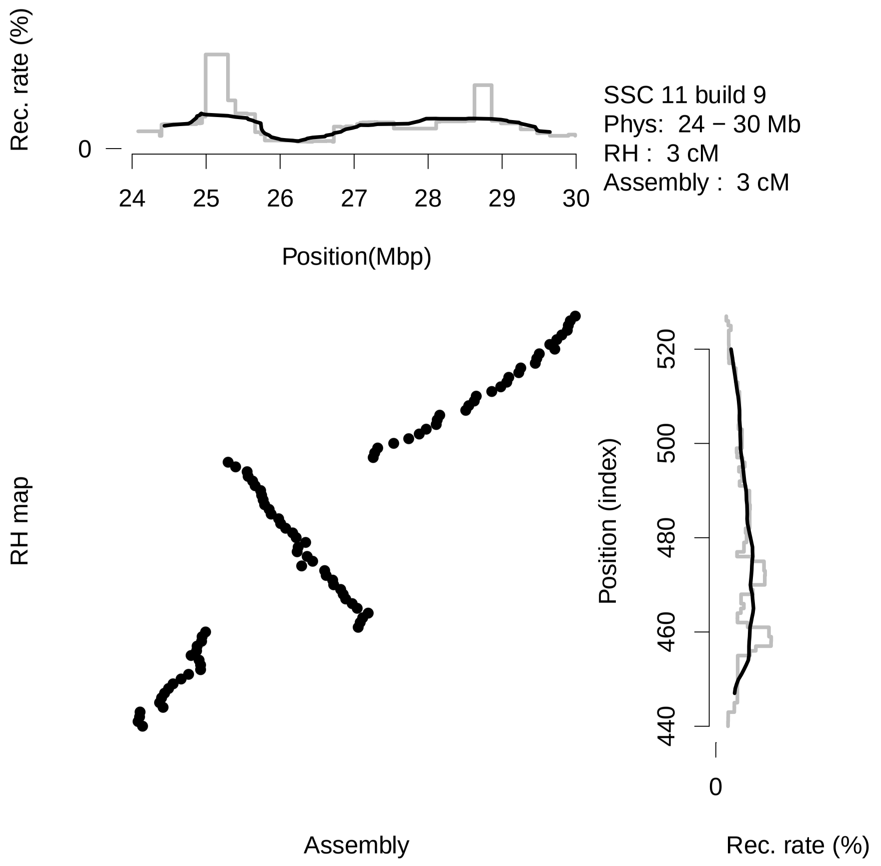

Rec. rate (%)
0
24
25
26
27
28
29
30
Position(Mbp)
SSC 11 build 9
Phys: 24 − 30 Mb
RH : 3 cM
Assembly : 3 cM
RH map
Assembly
Position (index)
440
460
480
500
520
Rec. rate (%)

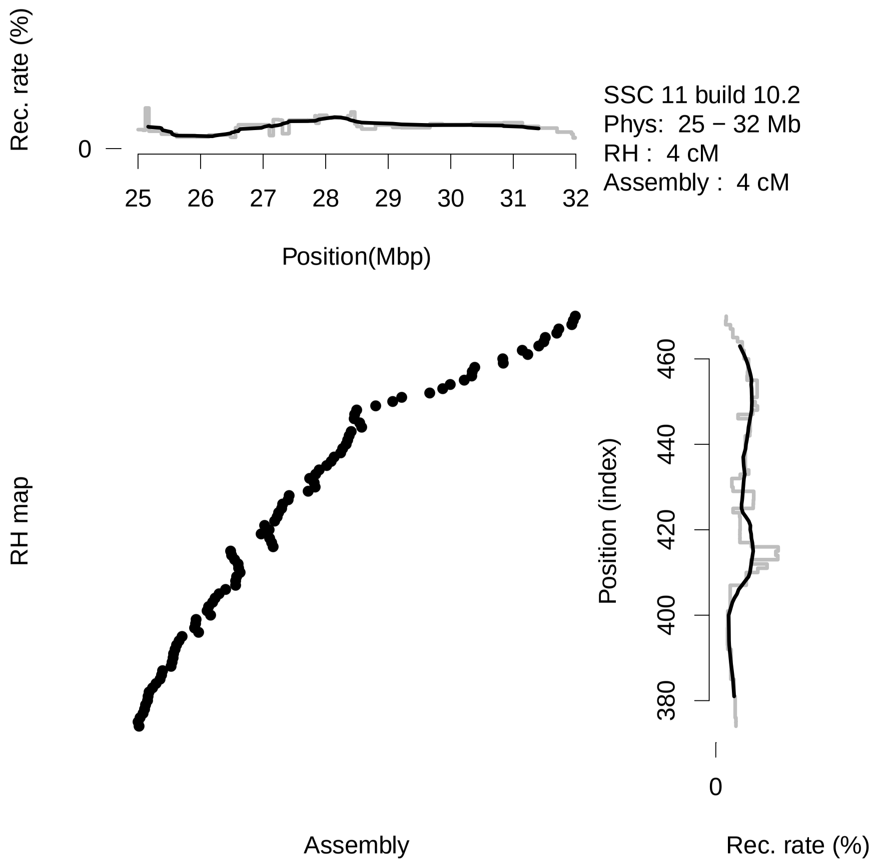

Rec. rate (%)
0
25
26
27
28
29
30
31
32
Position(Mbp)
SSC 11 build 10.2
Phys: 25 − 32 Mb
RH : 4 cM
Assembly : 4 cM
RH map
Assembly
Position (index)
380
400
420
440
460
Rec. rate (%)

SSC12

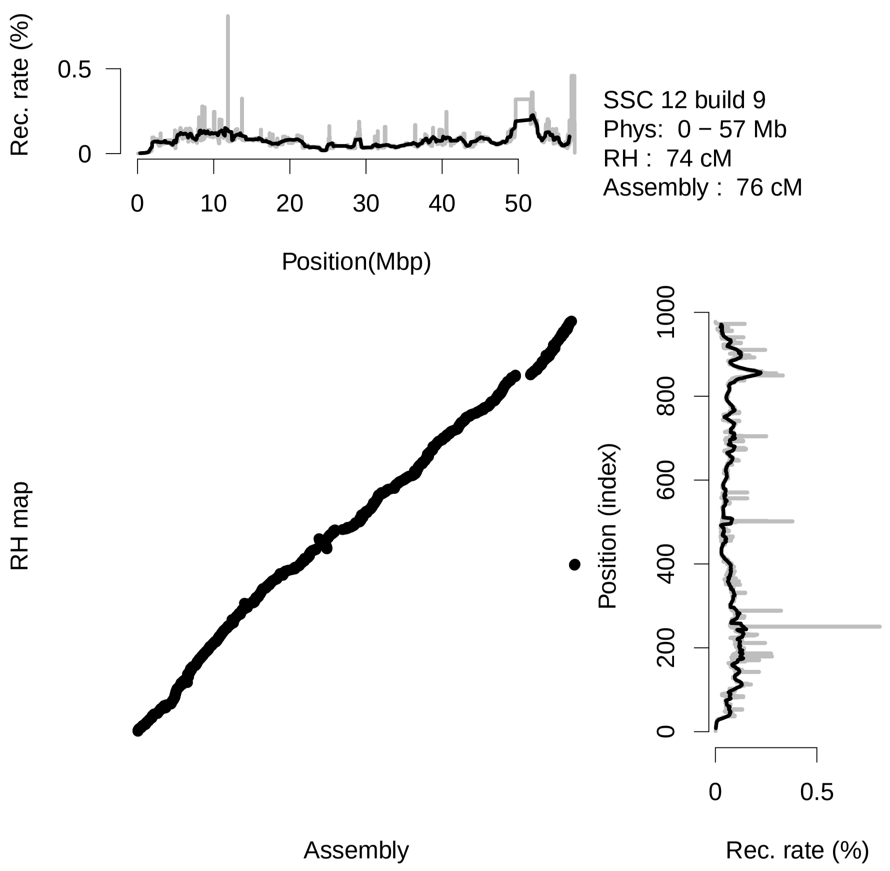
Rec. rate (%)
0.5
0
0
10
20
30
40
50
SSC 12 build 9
Phys: 0 − 57 Mb
RH : 74 cM
Assembly : 76 cM
Position(Mbp)
RH map
Position (index)
1000
800
600
400
200
0
0
0.5
Assembly
Rec. rate (%)

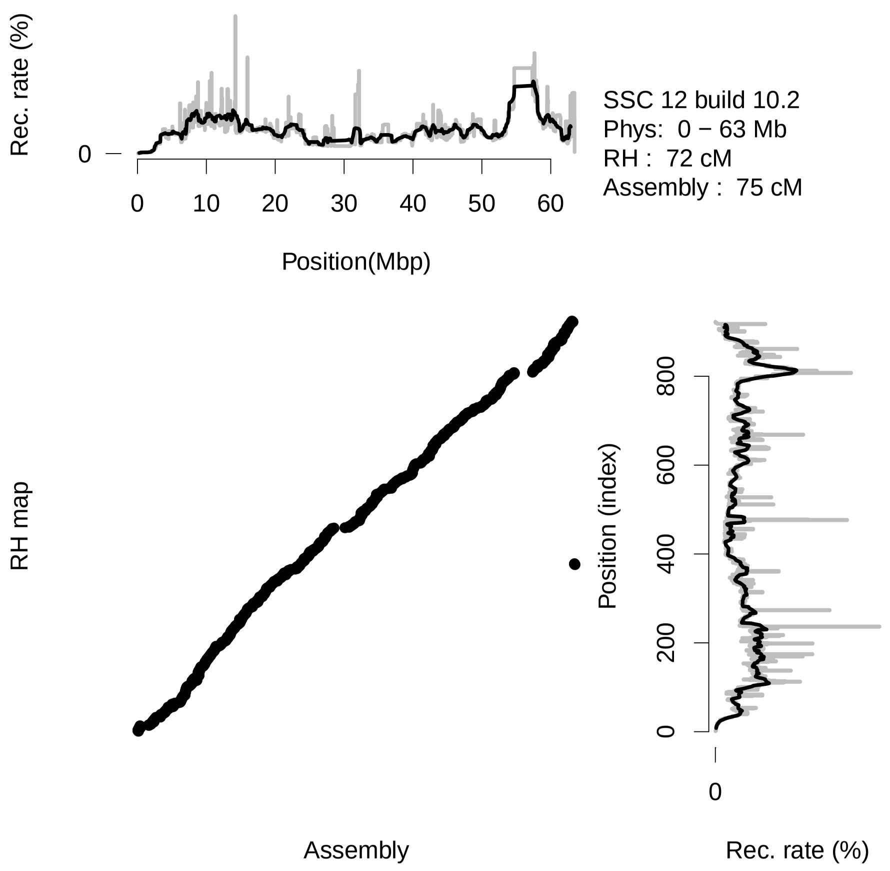
Rec. rate (%)
0
0
10
20
30
40
50
60
SSC 12 build 10.2
Phys: 0 − 63 Mb
RH : 72 cM
Assembly : 75 cM
Position(Mbp)
RH map
Position (index)
800
600
400
200
0
0
Assembly
Rec. rate (%)

SSC12 Zoom 1

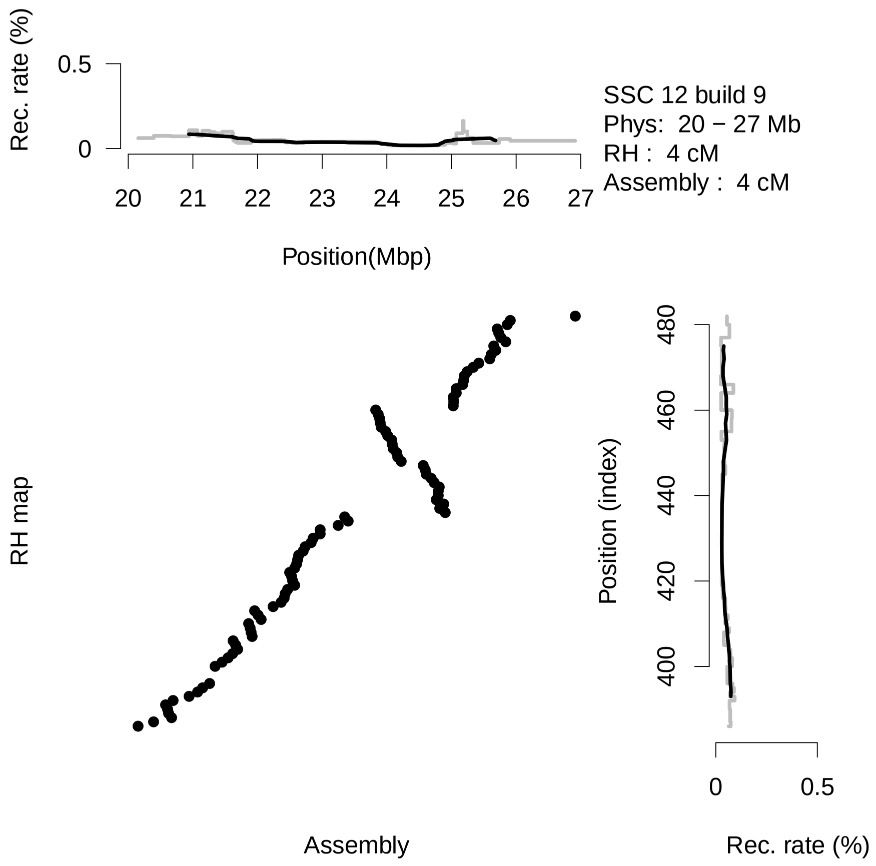

Rec. rate (%)
0.5
0
SSC 12 build 9
Phys: 20 − 27 Mb
RH : 4 cM
Assembly : 4 cM
20 21 22 23 24 25 26 27
Position(Mbp)
RH map
Position (index)
480 460 440 420 400
0 0.5
Assembly
Rec. rate (%)

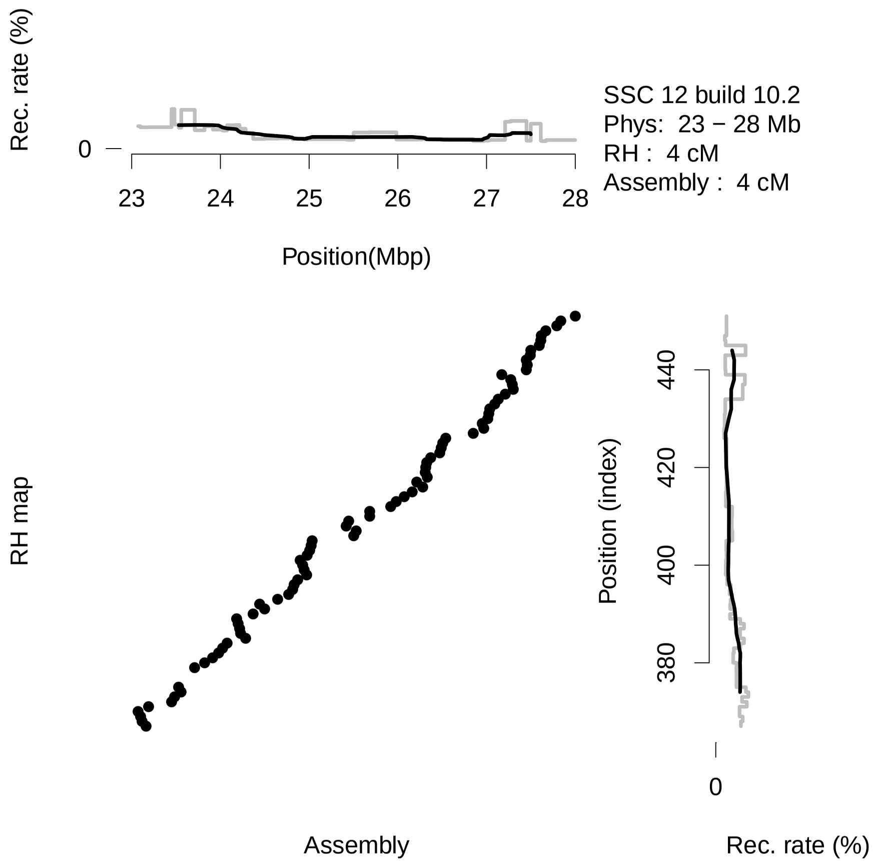

Rec. rate (%)
0
SSC 12 build 10.2
Phys: 23 − 28 Mb
RH : 4 cM
Assembly : 4 cM
23 24 25 26 27 28
Position(Mbp)
RH map
Position (index)
440 420 400 380
0
Assembly
Rec. rate (%)

SSC13

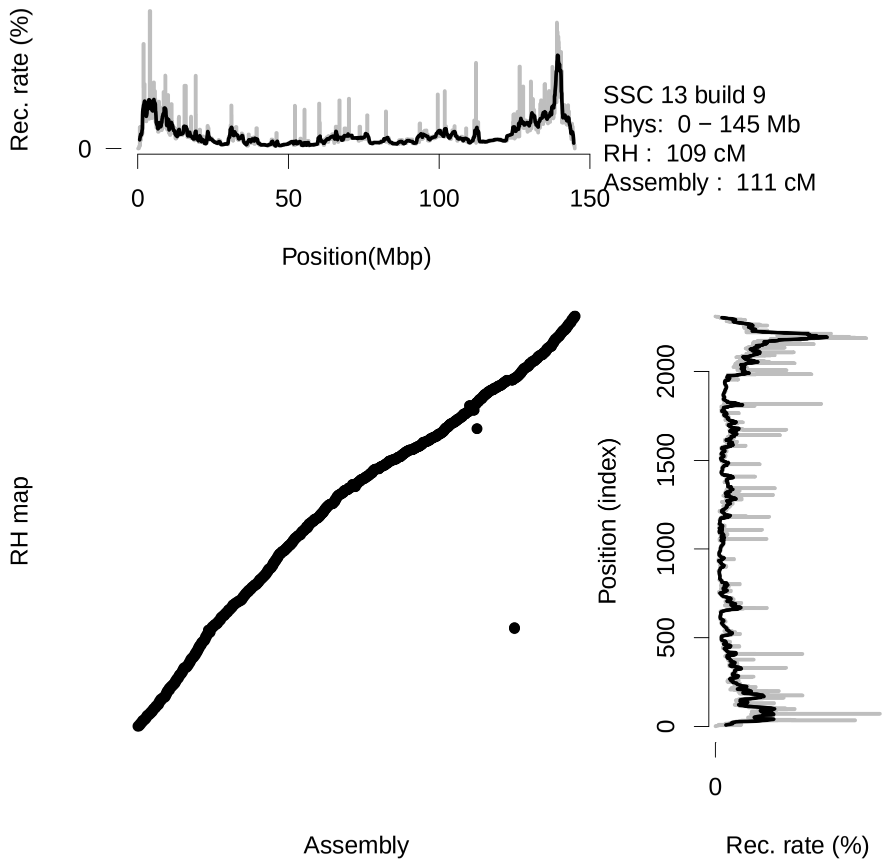

SSC 13 build 9
Phys: 0 − 145 Mb
RH : 109 cM
Assembly : 111 cM
Rec. rate (%)
Position(Mbp)
RH map
Assembly
Position (index)
Rec. rate (%)

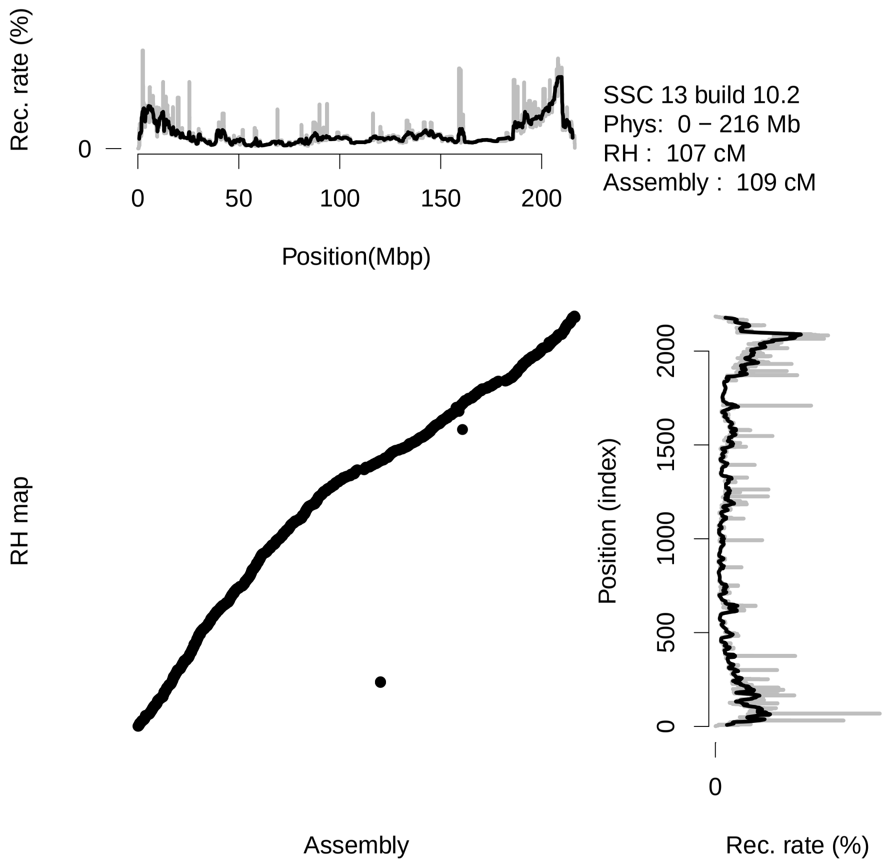

SSC 13 build 10.2
Phys: 0 − 216 Mb
RH : 107 cM
Assembly : 109 cM
Rec. rate (%)
Position(Mbp)
RH map
Assembly
Position (index)
Rec. rate (%)

SSC14

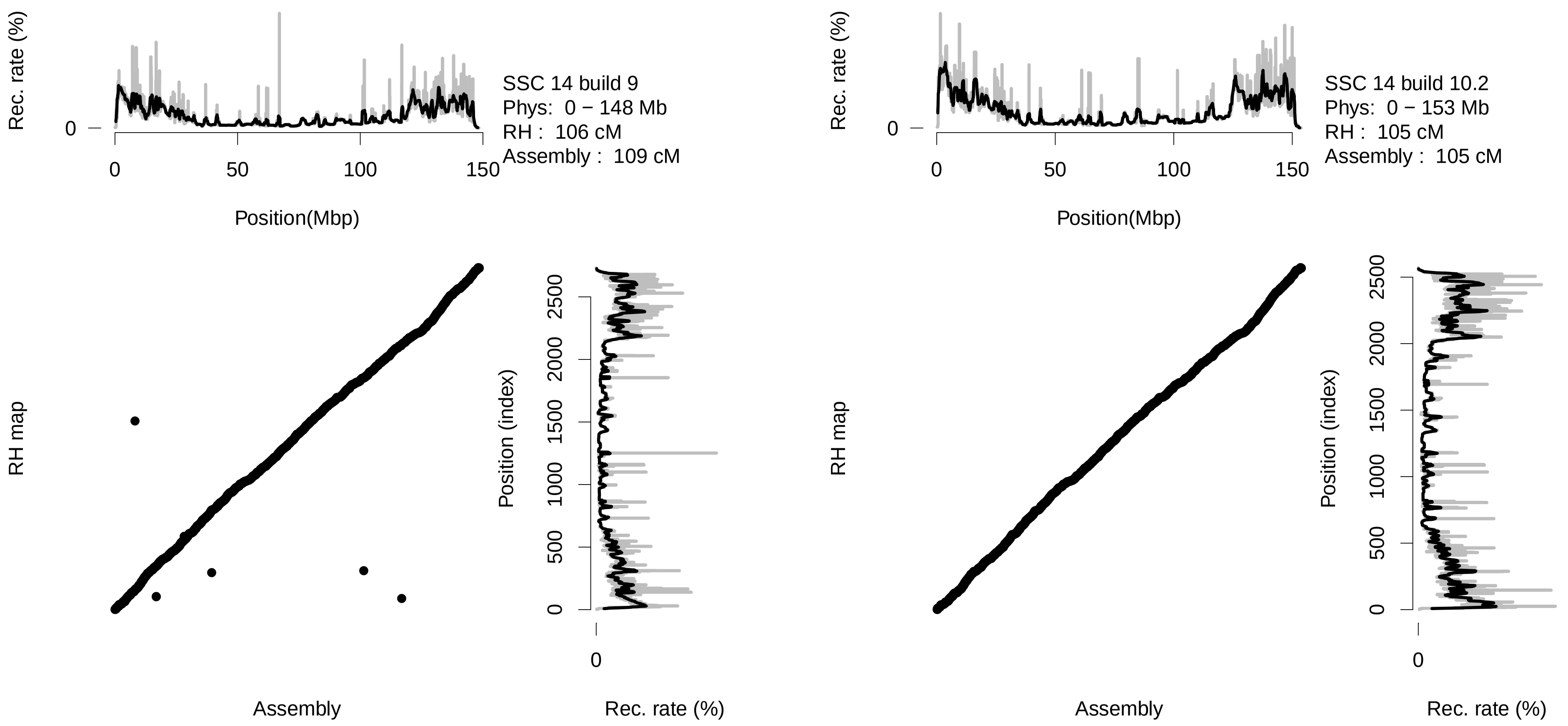

Rec. rate (%)
0
0
50
100
150
Position(Mbp)
SSC 14 build 9
Phys: 0 − 148 Mb
RH : 106 cM
Assembly : 109 cM
RH map
Assembly
Position (index)
0
500
1000
1500
2000
2500
Rec. rate (%)
SSC 14 build 10.2
Phys: 0 − 153 Mb
RH : 105 cM
Assembly : 105 cM

SSC15

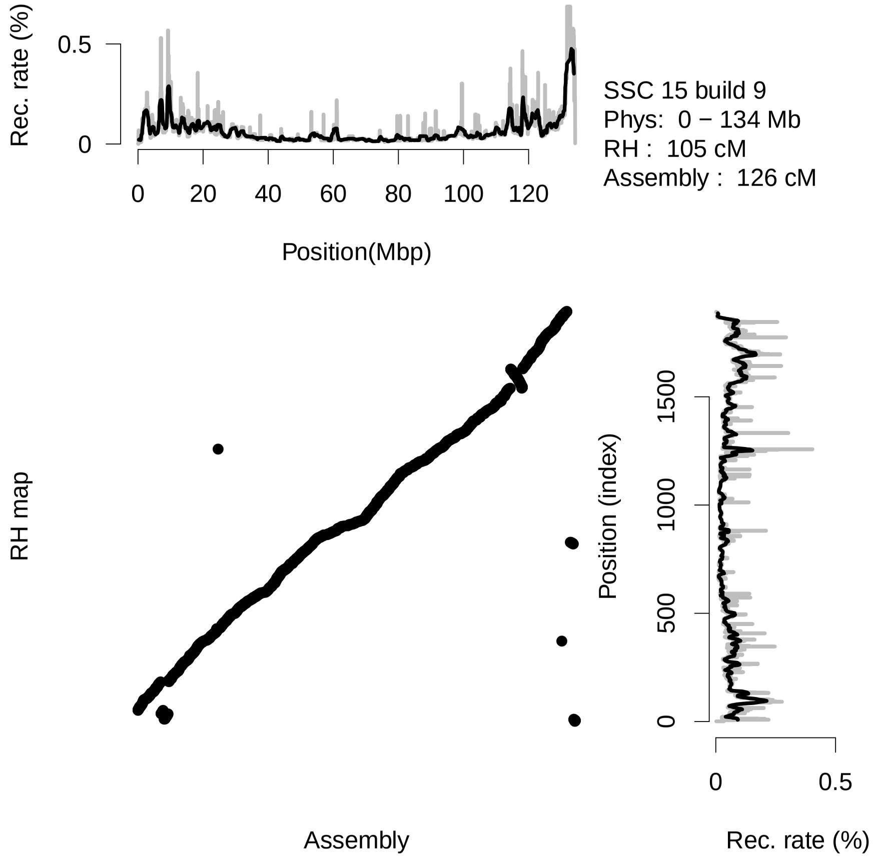

Rec. rate (%)
0.5
0
0
20
40
60
80
100
120
Position(Mbp)
SSC 15 build 9
Phys: 0 − 134 Mb
RH : 105 cM
Assembly : 126 cM
RH map
Assembly
Position (index)
0
500
1000
1500
0
0.5
Rec. rate (%)

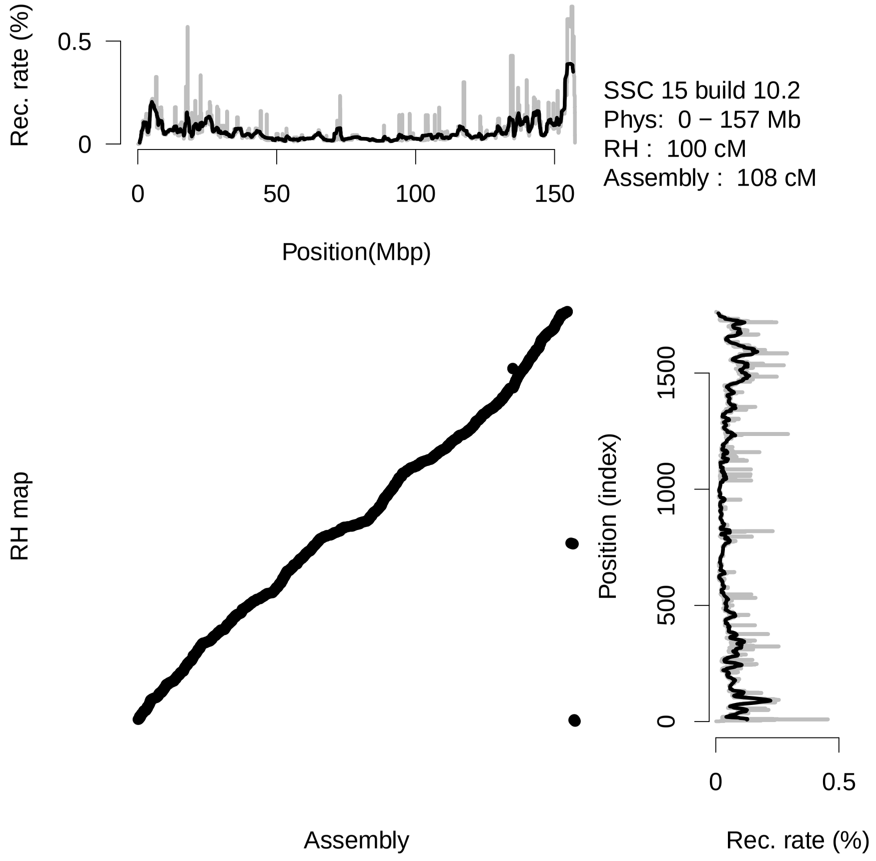

Rec. rate (%)
0.5
0
0
50
100
150
Position(Mbp)
SSC 15 build 10.2
Phys: 0 − 157 Mb
RH : 100 cM
Assembly : 108 cM
RH map
Assembly
Position (index)
0
500
1000
1500
0
0.5
Rec. rate (%)

SSC15 Zoom 1

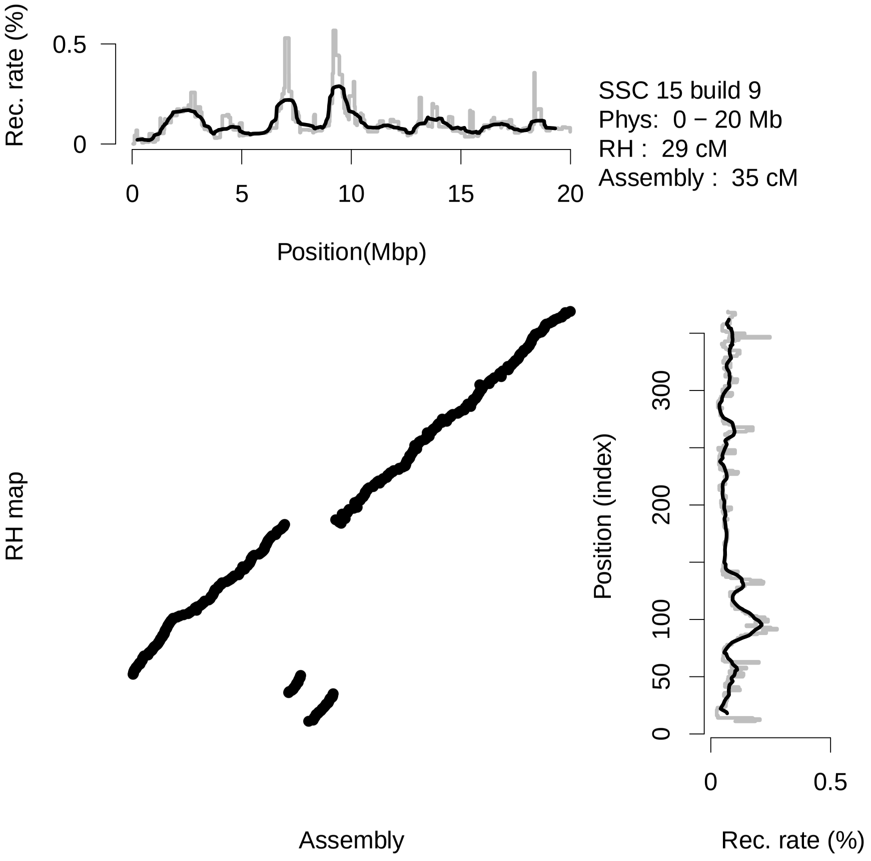
Rec. rate (%)
0.5
0
0
5
10
15
20
Position(Mbp)
SSC 15 build 9
Phys: 0 − 20 Mb
RH : 29 cM
Assembly : 35 cM
RH map
Assembly
Position (index)
0
50
100
200
300
0
0.5
Rec. rate (%)

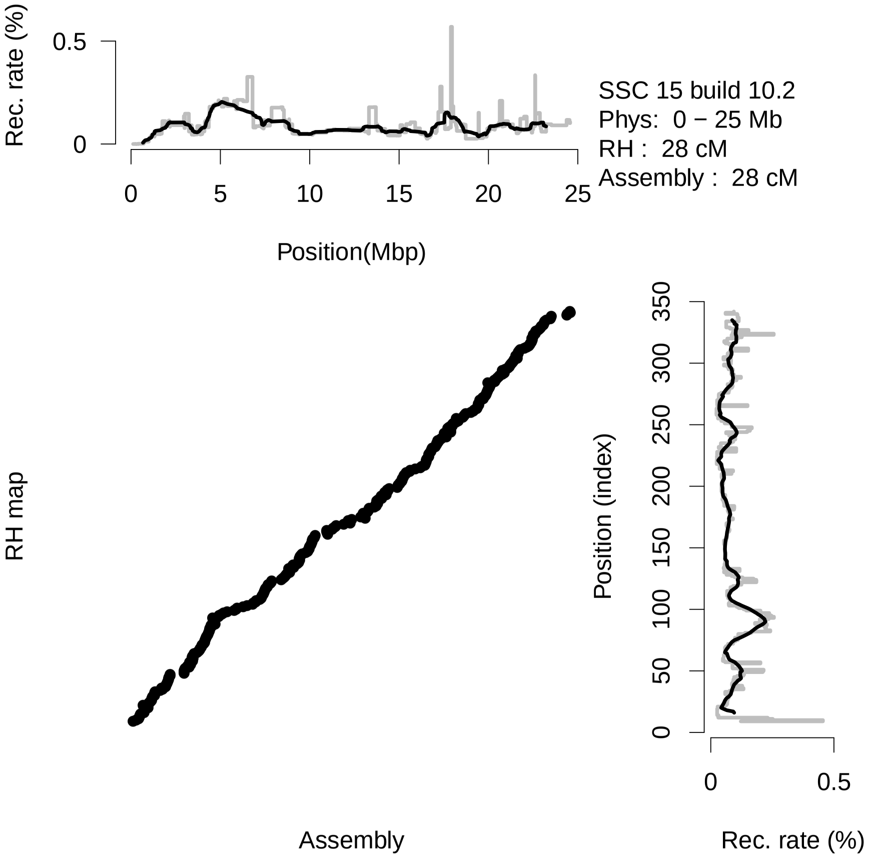
Rec. rate (%)
0.5
0
0
5
10
15
20
25
Position(Mbp)
SSC 15 build 10.2
Phys: 0 − 25 Mb
RH : 28 cM
Assembly : 28 cM
RH map
Assembly
Position (index)
0
50
100
150
200
250
300
350
0
0.5
Rec. rate (%)

SSC15 Zoom 2

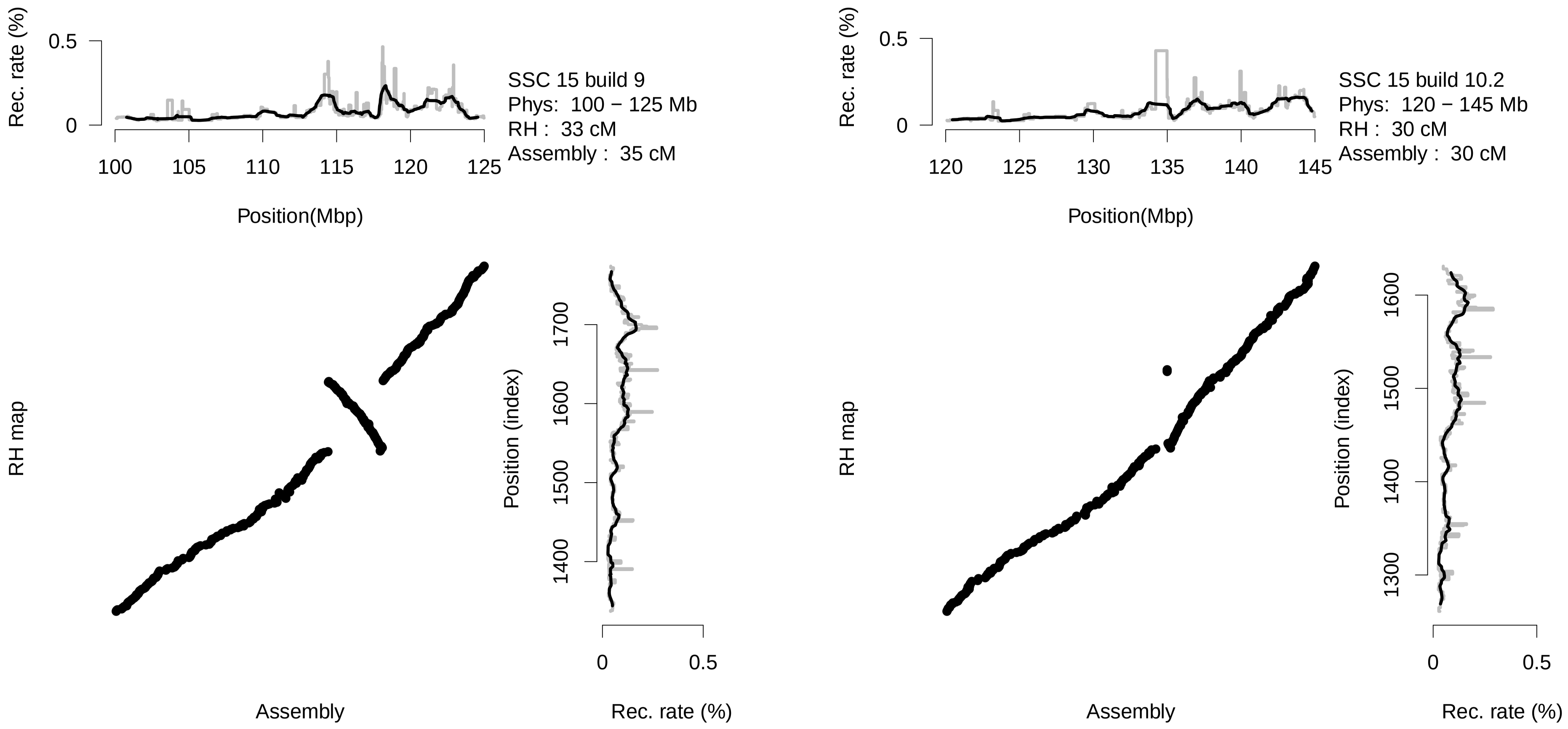

Rec. rate (%)
0.5
0
100
105
110
115
120
125
Position(Mbp)
SSC 15 build 9
Phys: 100 − 125 Mb
RH : 33 cM
Assembly : 35 cM
RH map
Assembly
Position (index)
1400
1500
1600
1700
Rec. rate (%)
0
0.5
Rec. rate (%)
0.5
0
120
125
130
135
140
145
Position(Mbp)
SSC 15 build 10.2
Phys: 120 − 145 Mb
RH : 30 cM
Assembly : 30 cM
RH map
Assembly
Position (index)
1300
1400
1500
1600
Rec. rate (%)
0
0.5

SSC16

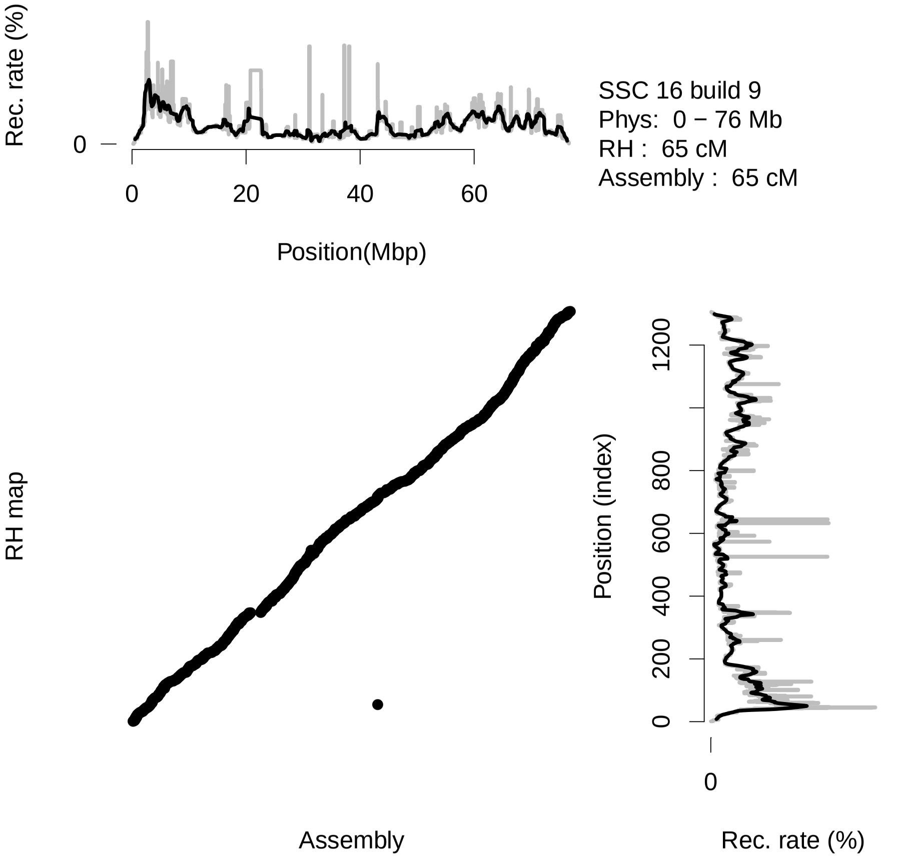
Rec. rate (%)
0
0
20
40
60
Position(Mbp)
SSC 16 build 9
Phys: 0 − 76 Mb
RH : 65 cM
Assembly : 65 cM
RH map
Assembly
Position (index)
0
200
400
600
800
1200
Rec. rate (%)

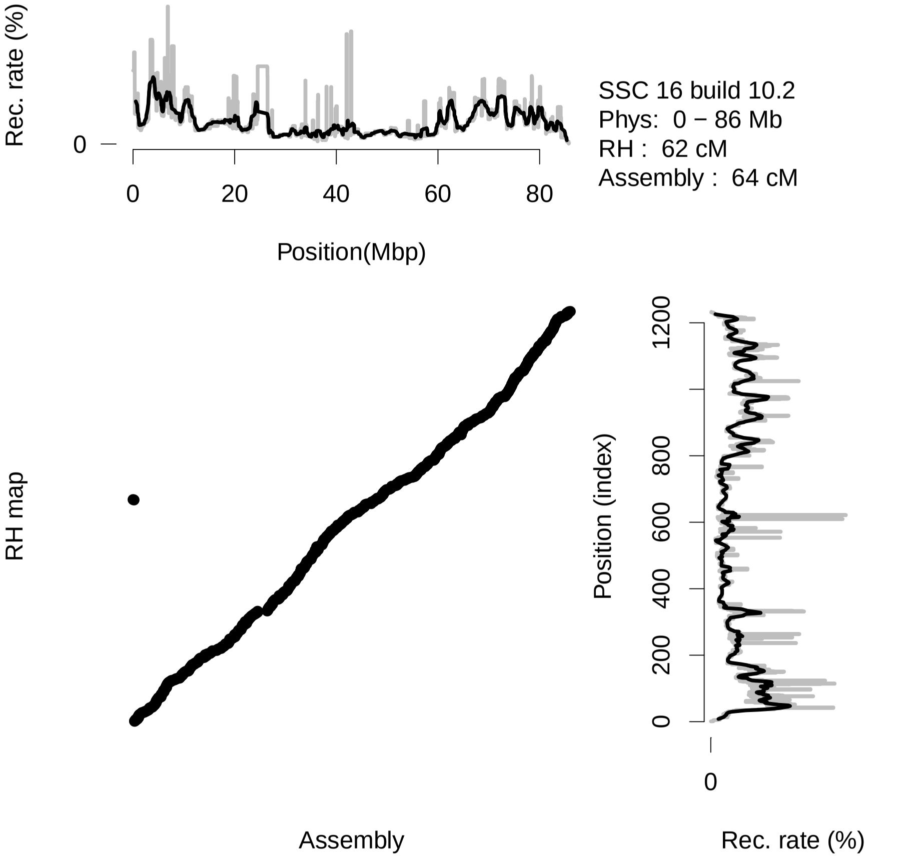
Rec. rate (%)
0
0
20
40
60
80
Position(Mbp)
SSC 16 build 10.2
Phys: 0 − 86 Mb
RH : 62 cM
Assembly : 64 cM
RH map
Assembly
Position (index)
0
200
400
600
800
1200
Rec. rate (%)

SSC17

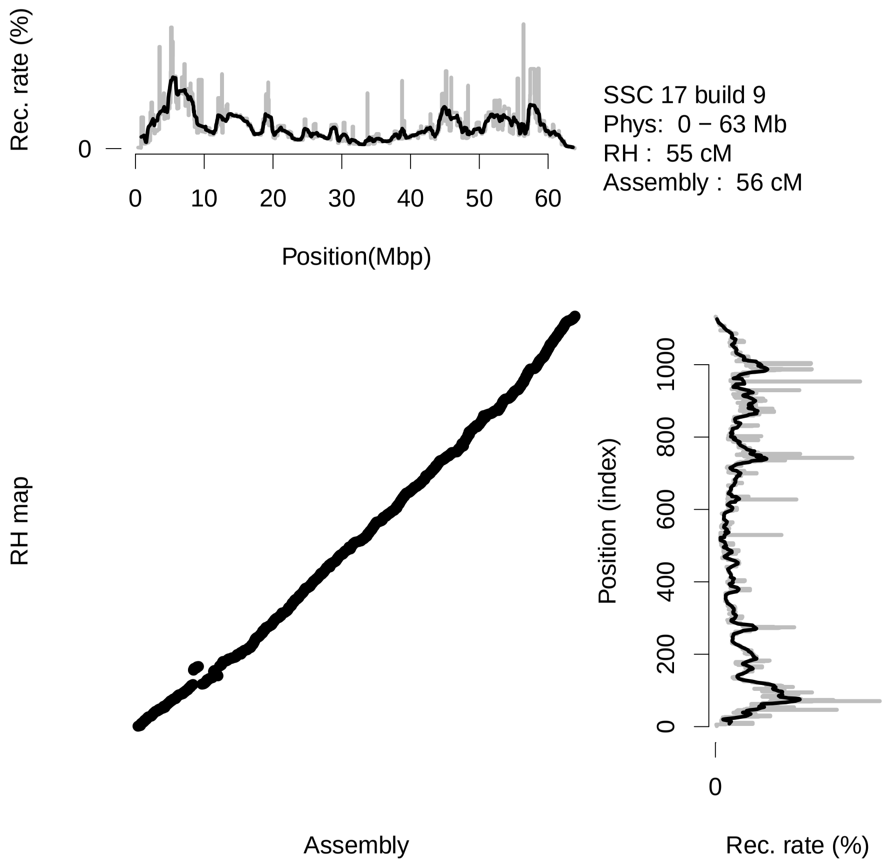

Rec. rate (%)
Position(Mbp)
SSC 17 build 9
Phys: 0 − 63 Mb
RH : 55 cM
Assembly : 56 cM
RH map
Assembly
Position (index)
Rec. rate (%)

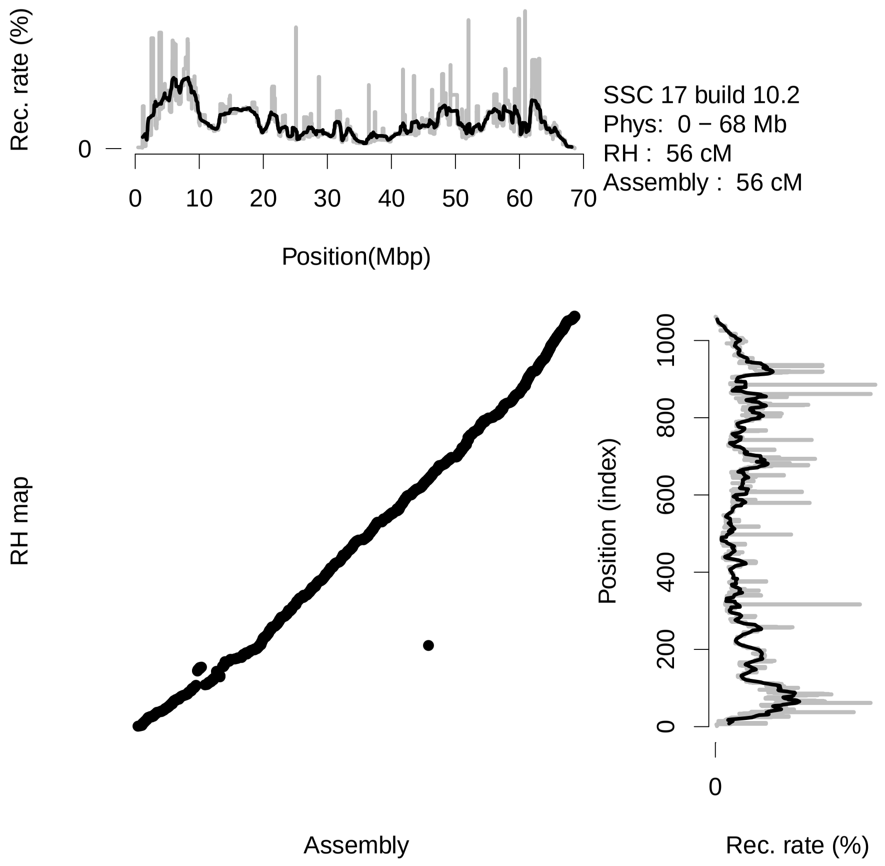

Rec. rate (%)
Position(Mbp)
SSC 17 build 10.2
Phys: 0 − 68 Mb
RH : 56 cM
Assembly : 56 cM
RH map
Assembly
Position (index)
Rec. rate (%)

SSC17 Zoom 1

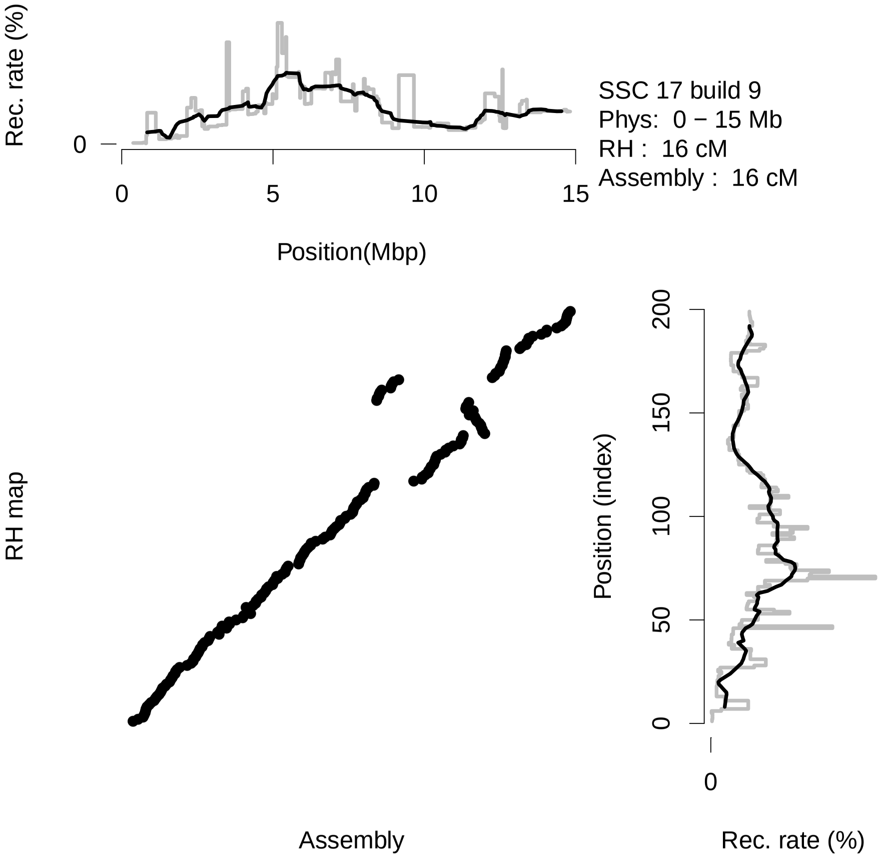
Rec. rate (%)
0
0
5
10
15
Position(Mbp)
SSC 17 build 9
Phys: 0 − 15 Mb
RH : 16 cM
Assembly : 16 cM
RH map
Assembly
Position (index)
0
50
100
150
200
0
Rec. rate (%)

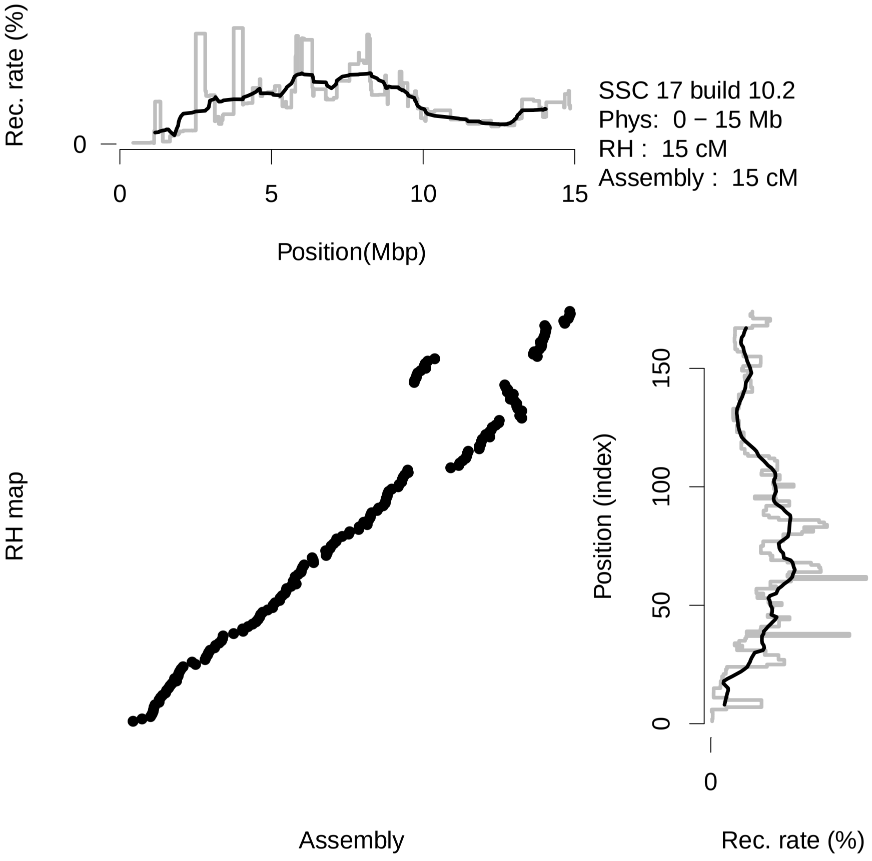
Rec. rate (%)
0
0
5
10
15
Position(Mbp)
SSC 17 build 10.2
Phys: 0 − 15 Mb
RH : 15 cM
Assembly : 15 cM
RH map
Assembly
Position (index)
0
50
100
150
0
Rec. rate (%)

SSC18

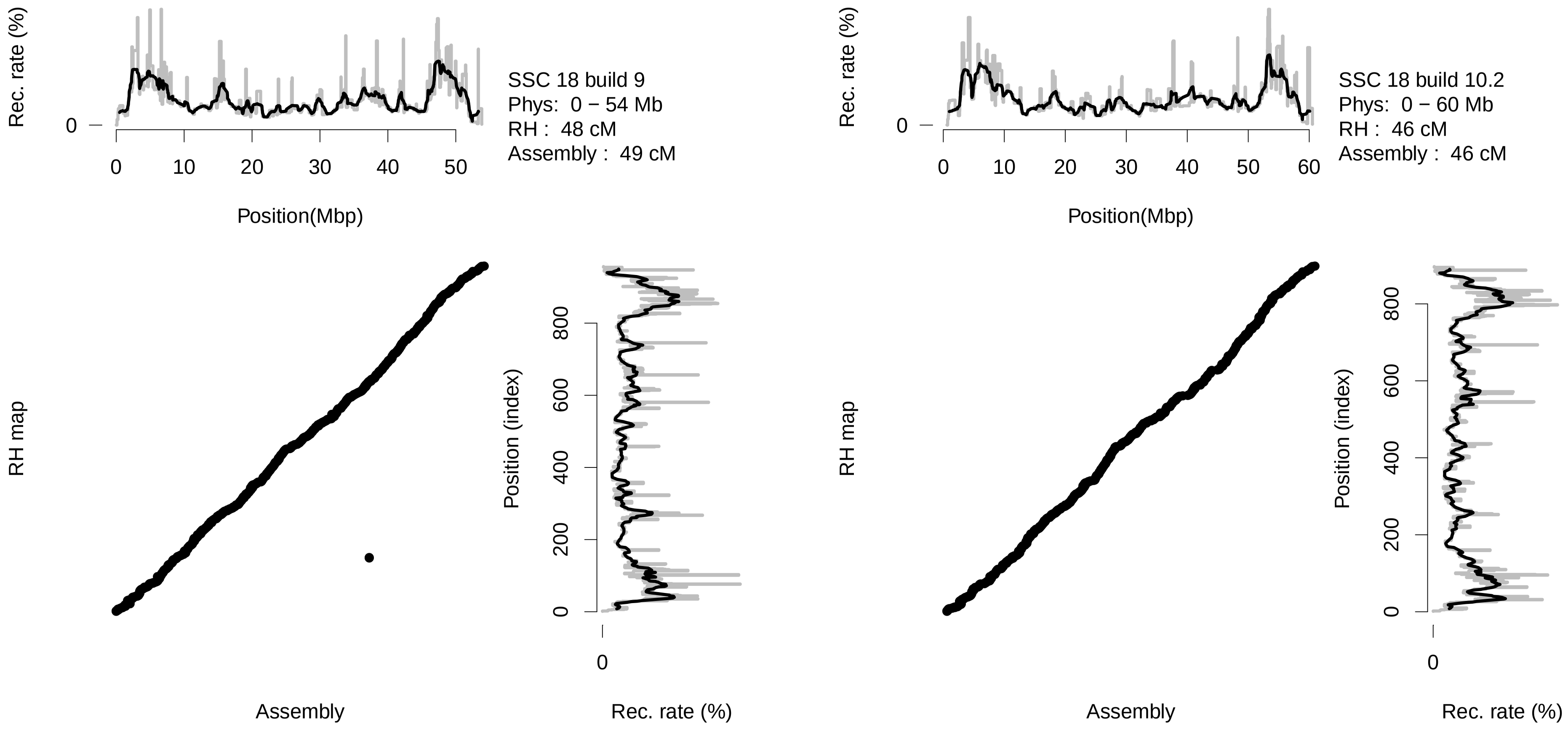

Rec. rate (%)
Position(Mbp)
SSC 18 build 9
Phys: 0 − 54 Mb
RH : 48 cM
Assembly : 49 cM
RH map
Assembly
Position (index)
Rec. rate (%)
Rec. rate (%)
Position(Mbp)
SSC 18 build 10.2
Phys: 0 − 60 Mb
RH : 46 cM
Assembly : 46 cM
RH map
Assembly
Position (index)
Rec. rate (%)
