# Supplementary material for: High-resolution autosomal radiation hybrid maps of the pig genome and their contribution to the genome sequence assembly
Source: BMC Genomics. 2012 Nov 15;13:585. doi: 10.1186/1471-2164-13-585 (PMC3499281; doi:10.1186/1471-2164-13-585)
Supplement: Additional file 6 — Validation of the predicted position for scaffold GL894031. This file contains figures and text presenting the validation of the predicted position for the unplaced scaffold GL894031. [file 1471-2164-13-585-S6.pdf]

## Synteny between Human and Pig

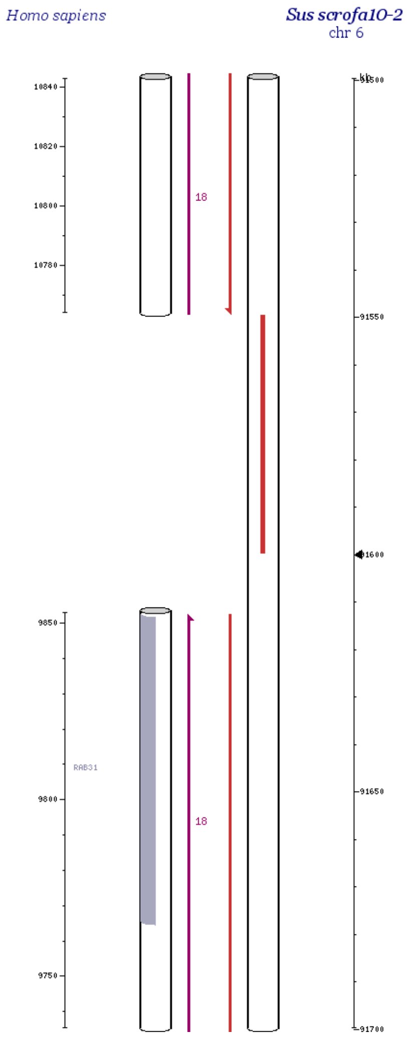


## Radiation Hybrid map

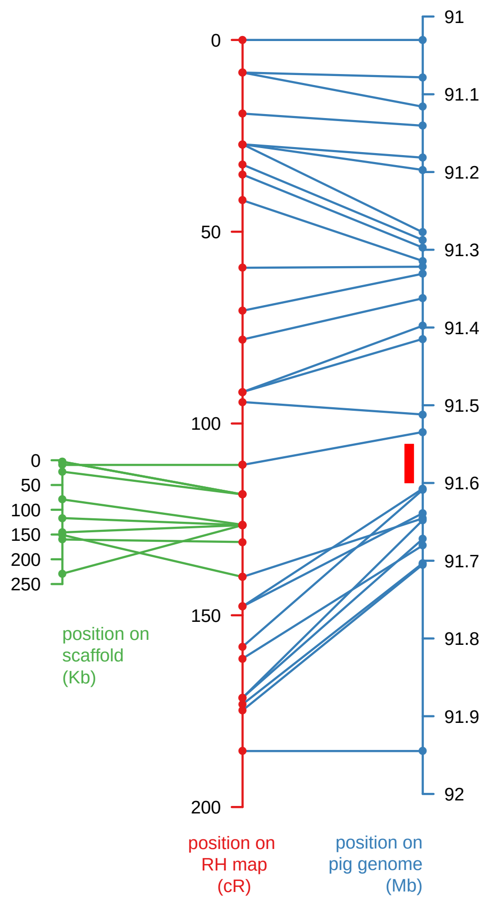


## Alignments of scaffold GL894031 with Human (left) and Pig (right)

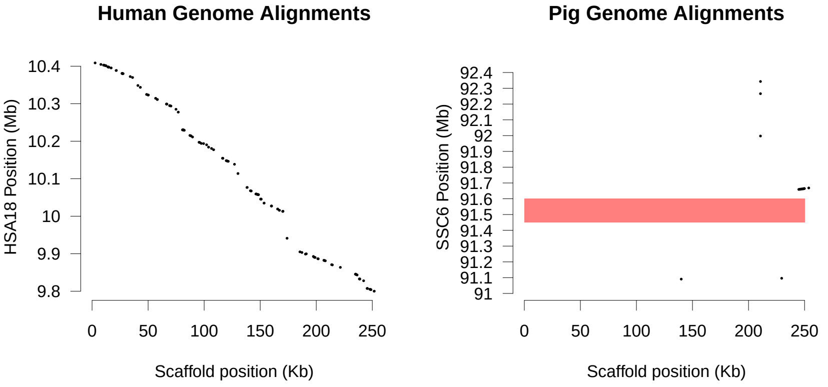


**Analysis of the predicted position of scaffold GL894031 on the pig genome** RH maps position the 10 SNPs matching the scaffold between 91.1 and 91.9 Mb on SSC6. This localisation corresponds to a gap in the current pig genome (red rectangles on the figures). The syntenic human chromosome is HSA 18 (top left). The scaffold aligns nicely with the human chromosome but does not align to the current pig genome (bottom). A local RH map confirms linkage between the SNPs of the scaffold and the surrounding SNPs on the pig genome (top right), placing it in the gap.
