# Supplementary figures and images for: High-resolution autosomal radiation hybrid maps of the pig genome and their contribution to the genome sequence assembly
Source: BMC Genomics. 2012 Nov 15;13:585. doi: 10.1186/1471-2164-13-585 (PMC3499281; doi:10.1186/1471-2164-13-585)

# multiple hits

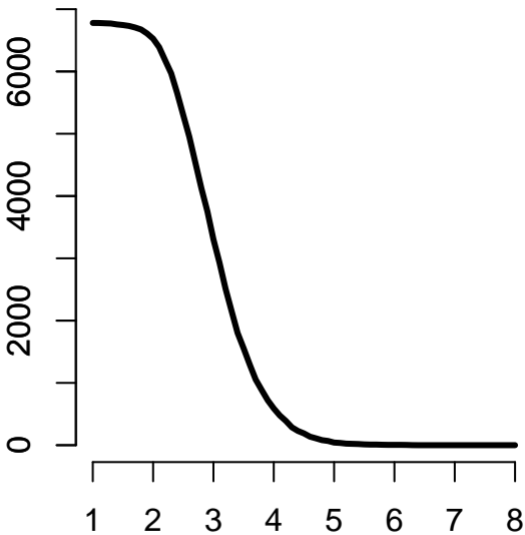

Similarity score

Supplement: Additional file 7 — Resolution of RH mapping for high density arrays. This file contains theoretical calculations on the resolution of RH panels and the design of RH experiments. [file 1471-2164-13-585-S7.pdf]
