# Supplementary material for: High-resolution autosomal radiation hybrid maps of the pig genome and their contribution to the genome sequence assembly
Source: BMC Genomics. 2012 Nov 15;13:585. doi: 10.1186/1471-2164-13-585 (PMC3499281; doi:10.1186/1471-2164-13-585)
Supplement: Additional file 2 — Characteristics of the RH maps. This table contains detailed characteristics of the RH maps obtained for each chromosome: number of SNPs mapped, number of distinct position for each panel, resolution, average marker distance and retention fraction. [file 1471-2164-13-585-S2.pdf]

				IMpRH panel (7000 rad)				IMNpRH2 Panel (12000 rad)			
SSC	size† (Mb)	SNPs	positions‡	positions‡	distance* (cR)	resolution (kb/cR)	retention (%)	positions‡	distance* (cR)	resolution (kb/cR)	retention (%)
1	307	5417	5180	4083	5.6	10	32	4808	9.0	6.3	33
2	118	2451	2373	1934	6.7	7.2	28	2197	10.5	4.6	34
3	104	2057	1963	1564	6.4	7.9	31	1842	10.7	4.7	34
4	126	2736	2639	2107	6.2	7.5	32	2431	8.3	5.6	36
5	92	1813	1773	1523	6.8	7.4	37	1631	10.0	5.1	36
6	140	2134	2058	1613	6.7	9.8	29	1916	12.4	5.3	33
7	107	2743	2643	2168	6.5	6.0	30	2409	9.0	4.3	31
8	114	2001	1915	1526	5.8	9.8	30	1763	10.5	5.4	34
9	115	2193	2108	1716	6.5	8.1	33	1922	9.5	5.5	35
10	52	1202	1159	876	5.5	7.9	36	1099	10.6	4.1	36
11	51	1406	1351	1060	6.6	5.5	30	1267	10.6	3.4	33
12	37	1047	992	781	6.9	5.1	28	904	10	3.6	29
13	201	2516	2393	1808	6.2	12.	21	2235	11.6	6.9	29
14	137	2916	2752	2014	5.2	9.0	25	2522	8.6	5.5	29
15	134	2074	1981	1555	5.4	12.0	31	1864	10.8	6.0	34
16	71	1413	1359	1092	6.1	8.3	41	1245	10.2	4.9	35
17	59	1232	1189	946	6.6	7.3	28	1121	9.6	5.1	35
18	51	1028	988	800	6.3	7.9	36	902	10.5	4.7	35
all	2016	38379	36816	29166	6.1	8.6	31	34078	9.9	5.3	33

Radiation-Hybrid map characteristics

†: size covered by SNPs on the RH map
‡: number of distinct positions on the maps
* : average distance between two markers
