# Supplementary material for: High-resolution autosomal radiation hybrid maps of the pig genome and their contribution to the genome sequence assembly
Source: BMC Genomics. 2012 Nov 15;13:585. doi: 10.1186/1471-2164-13-585 (PMC3499281; doi:10.1186/1471-2164-13-585)
Supplement: Additional file 8 — Number of uSNPs with multiple chromosome assignments against similarity score threshold. This file is a figure showing the evolution of the number uSNPs with multiple chromosome assignments as a function of the similarity score threshold. [file 1471-2164-13-585-S8.pdf]

Resolution of RH mapping for high density arrays

Bertrand Servin*, Thomas Faraut, Nathalie Iannuccelli, Diana Zelenica, Denis Milan.

Laboratoire de Génétique Cellulaire, Animal Genetics Division, INRA,

Email: Bertrand.Servin@toulouse.inra.fr;

*Corresponding author

We provide in this text theoretical considerations on the resolution of RH panels and RH maps. In RH mapping, the resolution can be defined in several ways. In the common sense, the resolution is the granularity of a map or a mapping tool: the smallest measurable distance that can be inferred by the tool. In RH mapping however, the physical mapping unit (Ray) is associated to the radiation dose of the panel (cR_{7000} and cR_{12000} for the two pig panels) and therefore does not directly reflect the physical distance. Because the relevant physical unit distance is really the base pair, the resolution of a panel or a map is frequently given as a Kb to cR ratio with the implicit assumption that the smallest measurable distance is 1cR. This Kb to cR ratio therefore provides a resolution in Kb in the common sense defined above. We will see that, with the typical size of the RH panels that have been constructed and used, the smallest measurable distance between two markers is generally larger than 1cR. Because what really matters is the possibility to map and order markers, a given map is also frequently characterized by a resolution, sometimes defined as the average distance between markers ([1, 2]). In this context of genome maps, the question of resolution can be addressed by estimating, for a given panel, the number of markers that can be mapped at distinct positions.

We therefore propose to tackle here the question of the resolution of the maps described in this study by addressing the two following questions:

1. Given an RH panel, what is the smallest measurable inter-marker distance ?
2. How many markers with distinct positions can we expect to position on a map for a given RH panel, and more generally when combining a set of RH panels ?

We will illustrate the theoretical results with the results obtained on the pig dataset.

Smallest measurable distance

First, we can define the resolution of a panel as the smallest measurable inter-marker distance that can be estimated by an RH mapping experiment. Let n be the panel size, r the average retention fraction and X_1 and X_2 the genotypes at two distinct markers M_1 and M_2. The **two-point** breakage probability between M_1 and M_2 is :

$$\theta = \frac{P(X_1 \neq X_2)}{2r(1-r)} \tag{1}$$

The smallest possible estimate of θ ($\hat{\theta}_{min}$) is obtained when a single hybrid among n discriminates the two RH vectors. In this case $P(X_1 \neq X_2)$ is estimated by $\frac{1}{n}$ and we have

$$\hat{\theta}_{min} = \frac{1/n}{2r(1-r)}$$

and since $\forall r$, $r(1-r) \leq \frac{1}{4}$ we have

$$\hat{\theta}_{min} \geq \frac{2}{n}$$

resulting in a maximal resolution of $\hat{d}_{min} = -\log(1-\hat{\theta}_{min}) = 2.2$ cR for a panel with 90 clones (the size of each panel in our experiment). The resolution, reaching its optimal value when $r = 1/2$, only depends on the number of clones in the panel.

In the case of **multipoint** estimates, missing data can no longer be ignored. The minimal inter-marker distance is no longer meaningful as in the presence of missing data the breakage frequencies have now a probabilistic interpretation and have no lower limit. Consider for example a set of k markers $M_1, \ldots, M_k$ with identical vectors except for the first hybrid where M_1 and M_k are respectively 0 and 1 for genotypes and $M_2, \ldots, M_{k-1}$ are all unknown for this hybrid, the breakage probability and hence the physical distance bewteen M_i and M_{i+1} is

$$\theta = \frac{1}{2nr(1-r)k}$$

The minimal distance decreases as long as the number of markers with an unknown genotype on the first hybrid increases.

The figure below illustrates the distances observed in the pig dataset, given as log ratios of the observed distance d to $\hat{d}_{min}$ to emphasize the impact of this smallest measurable distance.

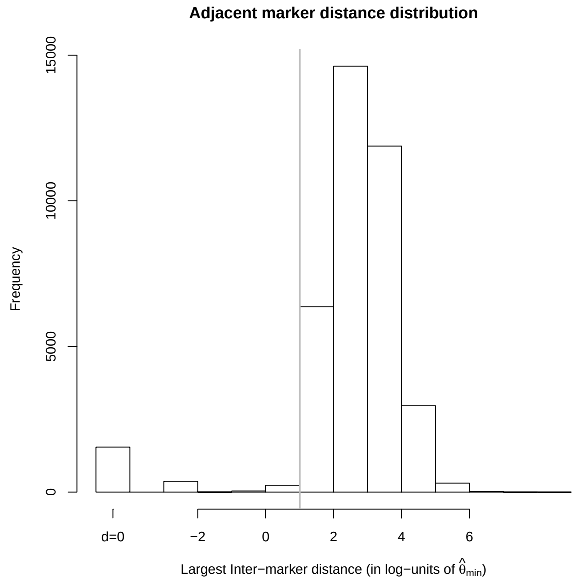

Estimates of the maximum distance between adjacent markers in the pig dataset. Distances are expressed in log units of the minimal two-point marker distance $\hat{d}_{min}$ *i.e.* $\log(d)/\log(\hat{d}_{min})$ where d is the distance in centiRays. The vertical gray line is placed at 1.
For each segment between adjacent markers, we have two distance estimates, one for each panel. The figure presents the distribution of the maximum of these two values in all segments of adjacent markers in the robust maps. Markers with $\log(d)/\log(\hat{d}_{min}) > 1$ can be considered as separated in the RH maps. They represent 94.3% of markers in the pig dataset. The remaining 5.7% can be considered as colocalized with at least one other marker.

The smallest measurable distance expressed in cR does not transpose directly to a resolution in Kb. With the usual assumption of a linear relationship between cR and Kb, the correspondence between cR and Kb can be estimated by comparing th map length in Rays to the corresponding size in Mb. The observed correspondence between cR and Kb, sometimes called resolution, are 8.6 Kb/cR and 5.3 Kb/cR for IMpRH and IMpRH2 respectively (maps totalizing 2292 and 3704 and Rays for a total of XXX Mb for the 18 autosomes), leading to a smallest measurable distance of 19 Kb and 11.6 Kb respectively.
This smallest measurable distance is a lower bound of the inter-marker distance and does not provide an estimation of the number of markers we can expect to map using a given panel, and in our case using two different panels. We address this question in the following sections.

Probablity of separating markers

We address here another aspect of the resolution that takes into account the sampling process of clones in an RH experiment. Consider two adjacent markers separated by a breakage probability of θ (*i.e.* by a distance $d = -\log(1-\theta)$ in centiRays). From equation 1, the probability of observing a break in a clone is:

$$p_b = P(X_1 \neq X_2) = 2\theta r(1-r)$$

and the probability of observing at least one clone with a break in a panel of size n is:

$$p_s = 1 - (1-p_b)^n = 1 - (1 - 2\theta r(1-r))^n$$

This probability depends on the radiation dosage (through θ), the retention fraction and the panel size.

The figure below plots p_s as a function of $d = -\log(1-\theta)$ for a panel of size 90 and a retention fraction of 0.3. For a distance greater than 16 centiRays, the probability of separating the markers is very close to 1. Interestingly, the probability of separating markers with true distance less than $\hat{d}_{min}$ is not negligible and in contrast about 20% of the markers that are distant of 6 cR cannot be separated.

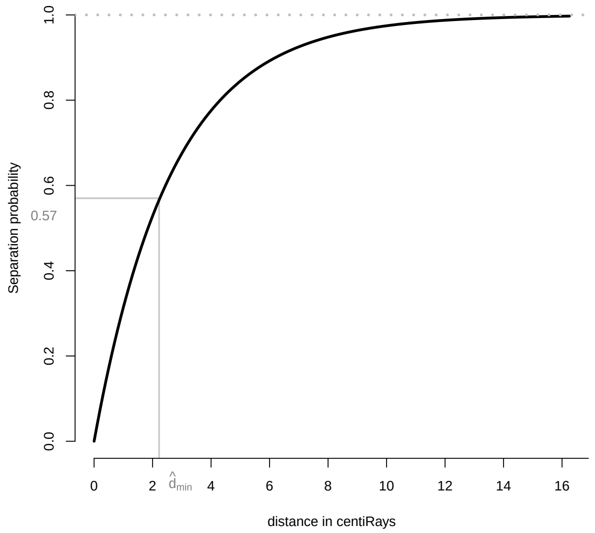

In the case of multiple panels with different resolutions like in our pig dataset, computing this probability is more complicated. Indeed, given two markers separated by L kilobases, the resulting breakage probabilities are different for each panel and depend on a resolution parameter γ (in kilobase per centiRay) that is unknown. This parameter is related to the radiation dose (expressed in Rads) but through a process too complex to be modelled. In the following, we will thus use our estimates of these parameters for the two pig RH panels: $\gamma_1 = 8.6$ Kb/cR and $\gamma_2 = 5.3$ Kb/cR. For two markers separated by L kilobases, the resulting distances in centiRays on the two panels are $d_1 = L/\gamma_1$ and $d_2 = L/\gamma_2$, with corresponding breakage probability θ_1 and θ_2. The probability of observing at least one clone with a break in the two panels combined is:

$$p_s = 1 - (1 - 2\theta_1 r_1(1-r_1))^{n_1}(1 - 2\theta_2 r_2(1-r_2))^{n_2}$$

where n_1 and n_2 are the respective sizes and r_1 and r_2 the respective retention fractions of panel 1 an 2. In the pig dataset, we have $n_1 = n_2$ and $r_1 \sim r_2$, so this equation simplifies to:

$$p_s = 1 - ((1 - 2\theta_1 r(1-r))(1 - 2\theta_2 r(1-r)))^n \qquad (2)$$

with $r = 0.3$ and $n = 90$.
The figure below presents the probability of separating two markers in each of the two pig panels (IMpRH and IMNpRH2) and the two combined (blue line) as a function of their physical distance (in Kb).

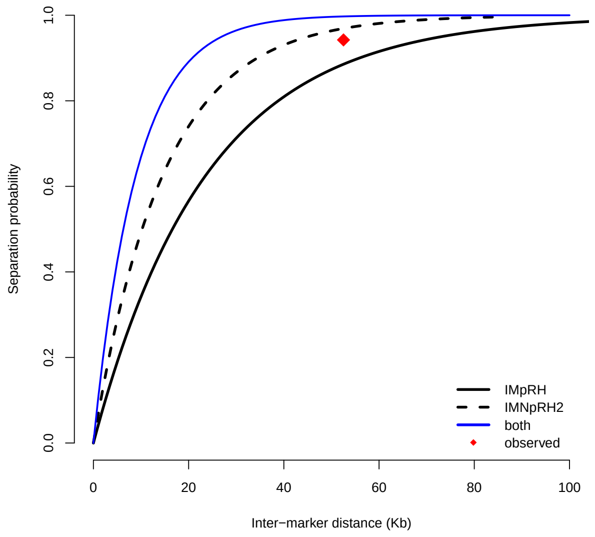

If we assume (i) that the true marker ordering is known and (ii) that markers are evenly spaced on the genome, then the separation probability is also the expected proportion of markers separated in an RH experiment. The red dot on the figure above corresponds to the results obtained on the pig dataset. This shows that our results match reasonably with the expectation, albeit we separate less markers than predicted by our theoretical calculations. However, this is easily explained as both hypotheses above do not hold precisely in our case, some of the real data are missing and most likely contain genotyping errors, which are not taken into account in the theoretical calculations.
Finally, using equation 2, we can predict how many markers could theoretically be separated using these two panels. The autosomal genome size on our map is $S \sim 2$ Giga-bases. If we assume N markers evenly spread on the genome, the average distance between markers $\delta \sim S/N$. We can use the above derviations to study how the number of separated markers varies with N. This is represented on the figure below, on a log-log scale.

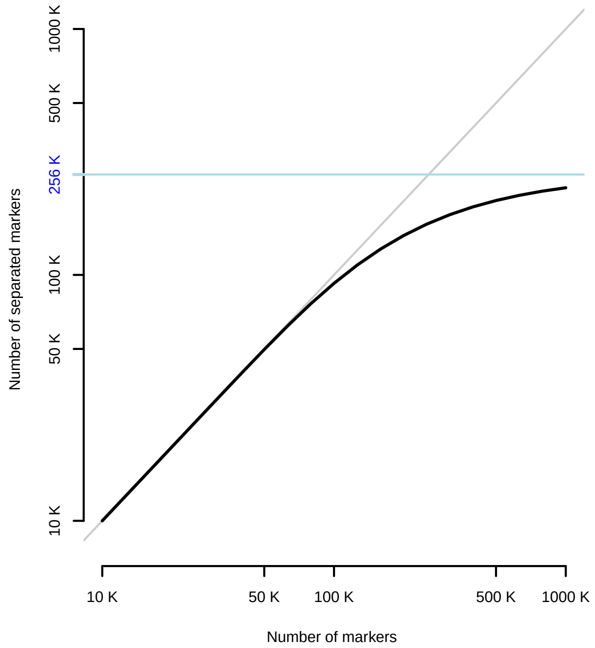

Number of separated markers as a function of the total number of markers on RH maps. Note that the plot uses a log-log scale.

The figure clearly shows a diminishing return above 100 K markers, *i.e.* the proportion of markers separated diminishes greatly above that point. For example, a map with one million markers will consist of only 250 K distinct positions. There is also an upper limit on the number of markers that can be separated by these two panels, about 256 K markers. These results must be taken as quite optimistic estimates. Indeed, to produce maps with around 36,000 markers on autosomes, we had to use an array of about 64,000 SNPs. Moreover, the observed proportion of unique positions was less than expected for reasons explained above. Our conclusion is that using arrays larger than 100 K SNPs is most likely not going to be cost-effective for producing high-density RH maps.

References

1. Marques E, de Givry S, Stothard P, Murdoch B, Wang Z, Womack J, Moore SS: **A high resolution radiation hybrid map of bovine chromosome 14 identifies scaffold rearrangement in the latest bovine assembly.** *BMC genomics* 2007, **8**:254, [http://www.pubmedcentral.nih.gov/articlerender.fcgi?artid=1959194\&tool=pmcentrez\&rendertype=abstract].

2. Prasad A, Schiex T, McKay S, Murdoch B, Wang Z, Womack JE, Stothard P, Moore SS: **High resolution radiation hybrid maps of bovine chromosomes 19 and 29: comparison with the bovine genome sequence assembly.** *BMC genomics* 2007, **8**:310, [http://www.pubmedcentral.nih.gov/articlerender.fcgi?artid=2064936\&tool=pmcentrez\&rendertype=abstract].
